# Integrated Expression Profiling and Genome-Wide Analysis of ChREBP Targets Reveals the Dual Role for ChREBP in Glucose-Regulated Gene Expression

**DOI:** 10.1371/journal.pone.0022544

**Published:** 2011-07-21

**Authors:** Yun-Seung Jeong, Deokhoon Kim, Yong Seok Lee, Ha-Jung Kim, Jung-Youn Han, Seung-Soon Im, Hansook Kim Chong, Je-Keun Kwon, Yun-Ho Cho, Woo Kyung Kim, Timothy F. Osborne, Jay D. Horton, Hee-Sook Jun, Yong-Ho Ahn, Sung-Min Ahn, Ji-Young Cha

**Affiliations:** 1 Department of Molecular Medicine, Lee Gil Ya Cancer and Diabetes Institute, Gachon University of Medicine and Science, Incheon, Korea; 2 Korean BioInformation Center (KOBIC), KRIBB, Daejeon, Korea; 3 Healthcare Service Group, Samsung SDS, Seoul, Korea; 4 Sanford-Burnham Medical Research Institute, Orlando, Florida, United States of America; 5 Department of Molecular Biology and Biochemistry, University of California Irvine, Irvine, California, United States of America; 6 Department of Molecular Genetics, University of Texas Southwestern Medical Center at Dallas, Dallas, Texas, United States of America; 7 Department of Biochemistry and Molecular Biology, Yonsei University College of Medicine, Seoul, Korea; 8 Department of Translational Medicine, Gachon University Gil Hospital, Incheon, Korea; South Texas Veterans Health Care System, United States of America

## Abstract

The carbohydrate response element binding protein (ChREBP), a basic helix-loop-helix/leucine zipper transcription factor, plays a critical role in the control of lipogenesis in the liver. To identify the direct targets of ChREBP on a genome-wide scale and provide more insight into the mechanism by which ChREBP regulates glucose-responsive gene expression, we performed chromatin immunoprecipitation-sequencing and gene expression analysis. We identified 1153 ChREBP binding sites and 783 target genes using the chromatin from HepG2, a human hepatocellular carcinoma cell line. A motif search revealed a refined consensus sequence (CABGTG-nnCnG-nGnSTG) to better represent critical elements of a functional ChREBP binding sequence. Gene ontology analysis shows that ChREBP target genes are particularly associated with lipid, fatty acid and steroid metabolism. In addition, other functional gene clusters related to transport, development and cell motility are significantly enriched. Gene set enrichment analysis reveals that ChREBP target genes are highly correlated with genes regulated by high glucose, providing a functional relevance to the genome-wide binding study. Furthermore, we have demonstrated that ChREBP may function as a transcriptional repressor as well as an activator.

## Introduction

Glucose is a vital energy nutrient that provides carbon for biosynthetic reactions and ATP for energy. Most organisms have evolved diverse and sophisticated mechanisms for sensing glucose and using it efficiently, including mechanisms for glucose-dependent regulation of gene transcription. In mammals, the molecular basis of glucose-regulated gene transcription was revealed by the discovery and characterization of the carbohydrate response element binding protein (ChREBP, MondoB and WBSCR14), which is a glucose-responsive transcription factor [1], [2].

ChREBP is a basic helix-loop-helix leucine zipper transcription factor, mediating glucose-regulated gene transcription. Upon activation by glucose, ChREBP, whose expression is most prominent in the liver, translocates from the cytosol into the nucleus [3]. In the nucleus, ChREBP forms a heterodimer with Max-like protein X (Mlx) to bind to the carbohydrate response element (ChoRE) for transcriptional regulation of its target genes [4], [5], [6]. ChREBP plays a critical role in hepatic lipogenesis in response to high carbohydrate diet, converting excess glucose to storage lipid. In ChREBP null mice, glycolytic and lipogenic genes are not induced by high carbohydrate diet, leading to reduced lipogenesis [7]. Loss of ChREBP in leptin-null *ob/ob* mice alleviates obesity and corrects hepatic steatosis [8], [9].

To fully understand the molecular basis of glucose-regulated gene transcription mediated by ChREBP, it is essential to identify ChREBP target genes and their roles. It is known that ChREBP regulates various enzymes involved in glycolysis and lipogenesis such as pyruvate kinase, liver and RBC (PKLR), acetyl-CoA carboxylase and fatty acid synthase (FASN) [10], [11]. The full transcriptional regulatory circuitry of ChREBP, however, is yet to be understood. In the present study, we aimed to identify ChREBP target genes and their roles on a genome-wide scale using chromatin immunoprecipitation combined with massively parallel sequencing technology (ChIP-seq) and gene expression analysis [12], [13].

## Results

### Identification and characterization of ChREBP binding sites using ChIP-seq analysis

To identify the complete repertoire of ChREBP target genes, we treated HepG2 cells with 25 mM glucose for 8 h. This experimental condition was determined by time course experiments that measured the expression level changes of known ChREBP target genes in response to 25 mM glucose (Figure S1). Following initial testing of chromatin quality and ChIP efficiency by ChIP-quantitative real-time PCR (ChIP-qPCR, data not shown), ChIPed DNA and its control DNA were subjected to deep sequencing. We obtained a total of 6,679,066 36-nucleotide sequence tags from ChIPed DNA, and 5,992,478 sequence tags from the control, which were uniquely mapped to the reference genome, respectively.

**Figure 1:**
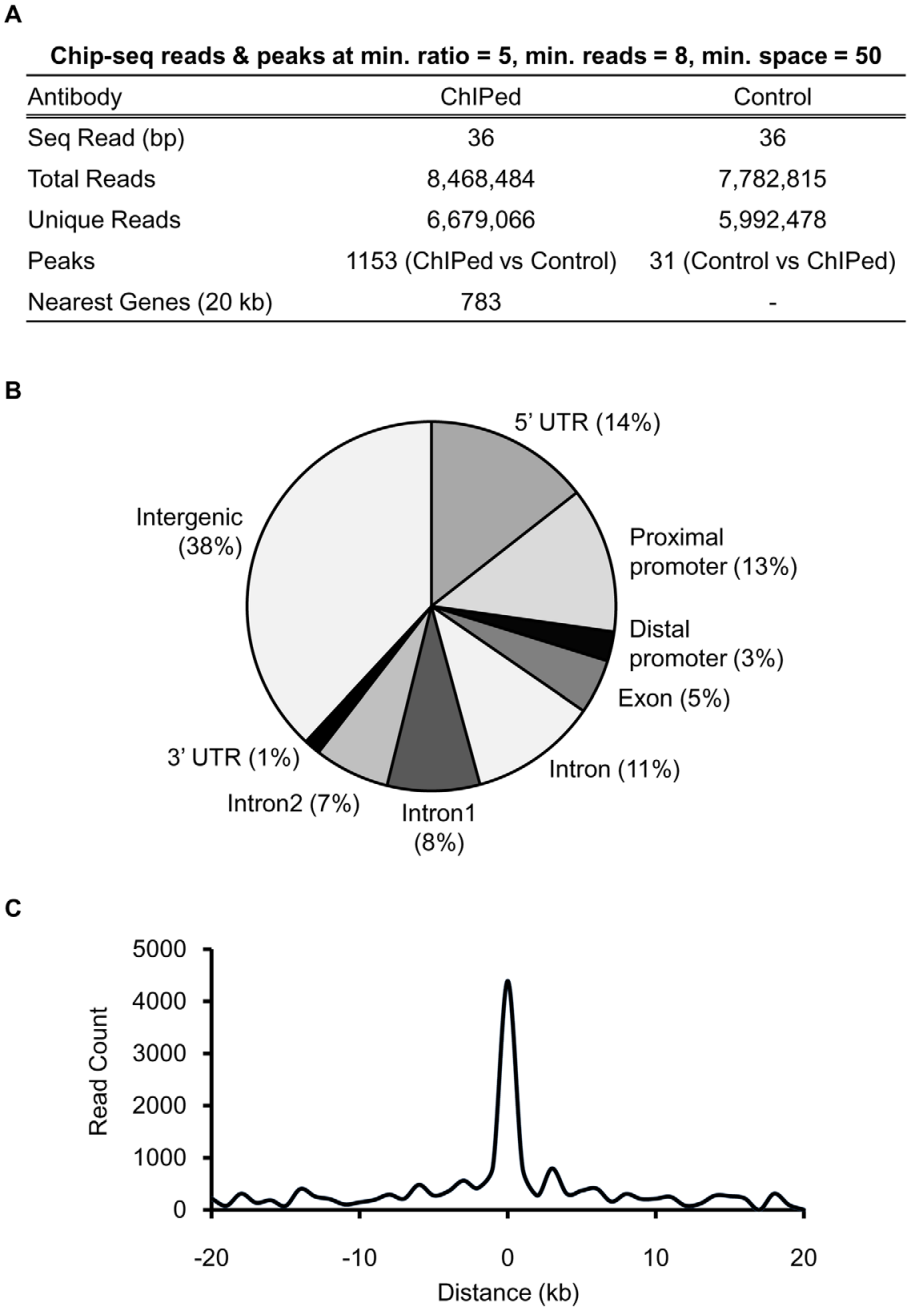
ChIP-seq analysis for ChREBP–DNA binding in human liver cells, HepG2. (A) Summary of peak analysis. (B) Location of ChREBP binding peaks relative to known genes. The proximal and distal promoters are defined as 2 kb and 5 kb of 5′-flanking DNA, respectively. The majority of sites (38%) are located within an intergenic region; 16% are located in promoter regions. (C) Peak distance relative to TSS of the closest gene. Negative distances indicate regions upstream of TSSs; positive distances indicate regions downstream of TSSs. Note that only the region around the TSS is shown.

ChREBP binding sites and their associated genes were identified using PeakFinder [14] and CisGenome [15]. We identified a total of 1153 ChREBP binding sites with an average width of 398 bp (Figure 1A). We searched for the peak proximal genes (<20 kb on both sides) and identified total 783 nearest genes. Additional information on the peak locations and their nearest genes is provided in Table S1. We categorized ChREBP binding sites based on their relative positions to the nearest genes using the University of California at Santa Cruz genome annotation database for the human genome (hg18, Build 36.1) [16]. According to our analysis, 16% of ChREBP binding sites were located within promoters and 13% of ChREBP binding sites were located within 2 kb upstream region of the transcription start site (TSS) of annotated genes (Figure 1B). A large proportion (38%) of ChREBP binding sites was located within intergenic regions. Approximately 26% of peaks were localized within introns; 15% of peaks were localized to the first or second introns. The remaining sites were distributed mostly within 5′ UTRs (14%) with very few located in 3′ UTRs (1%) and exons (5%). A similar genome-wide distribution pattern was found in ChIP-Seq data for the transcription factors STAT1, CTCF and NRSF [17]. A histogram of ChREBP binding sites located within the genomic regions 20 kb downstream or upstream relative to annotated TSSs shows clearly that most of the ChREBP binding sites are enriched around the TSSs (Figure 1C).

### Confirmation of identified ChREBP binding sites

**Figure 2:**
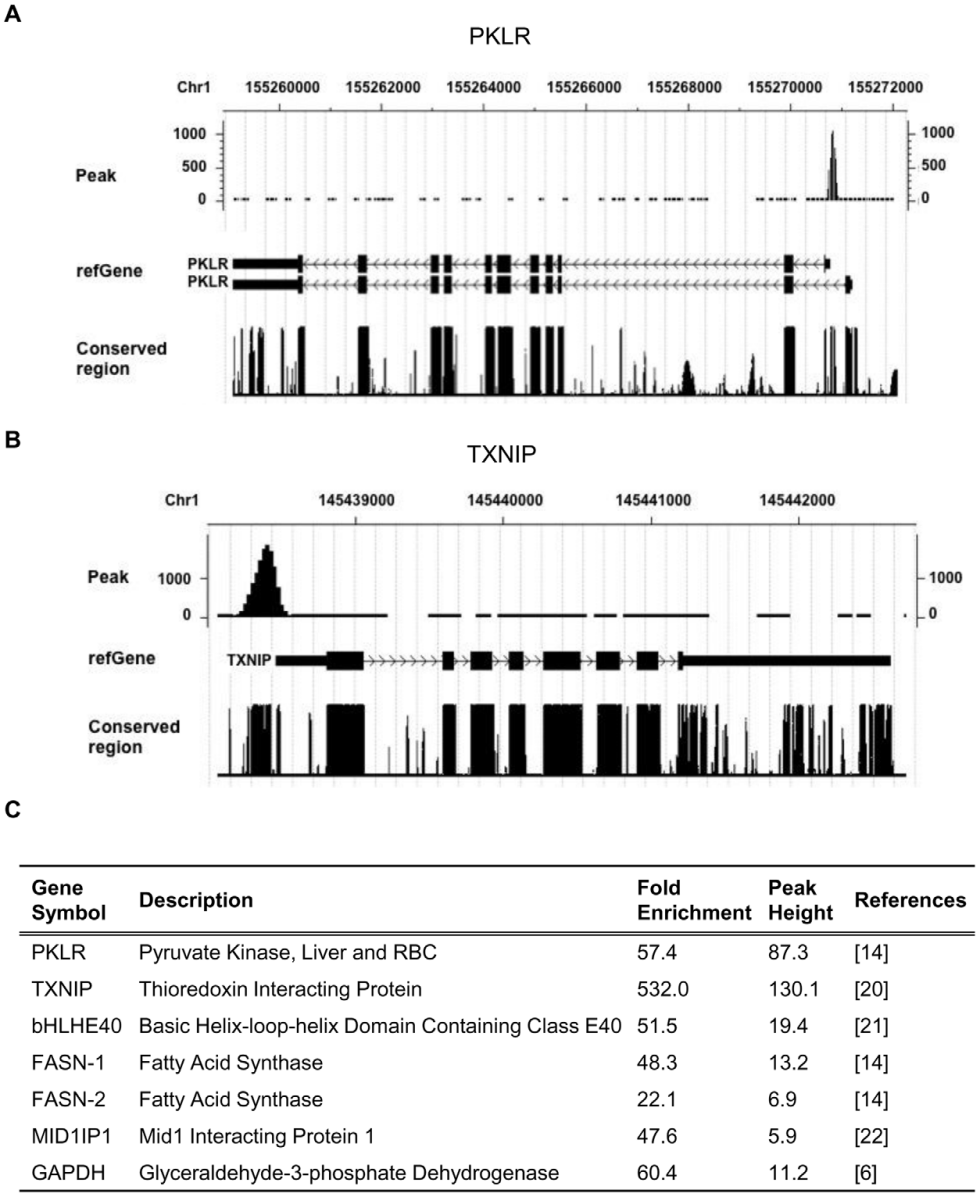
Characterization of ChREBP binding sites at selected gene loci. (A, B) CisGenome Browser screenshots of peaks associated with the PKLR and TXNIP genes. The *y*-axis shows the number of mapped tags. Annotations are from the UCSC Genome Browser. (C) Seven ChREBP binding sites in six target genes showing peak height and fold enrichment. ChIP-qPCR was performed to confirm the identified ChREBP binding sites. The fold enrichment is the fold increase for the signal from ChREBP ChIPed DNA relative to control IgG. Cyclophilin (Cyclo) and PKLR-4 kb were used as negative controls (0.98- and 1.2-fold enrichment, respectively).

**Figure 3:**
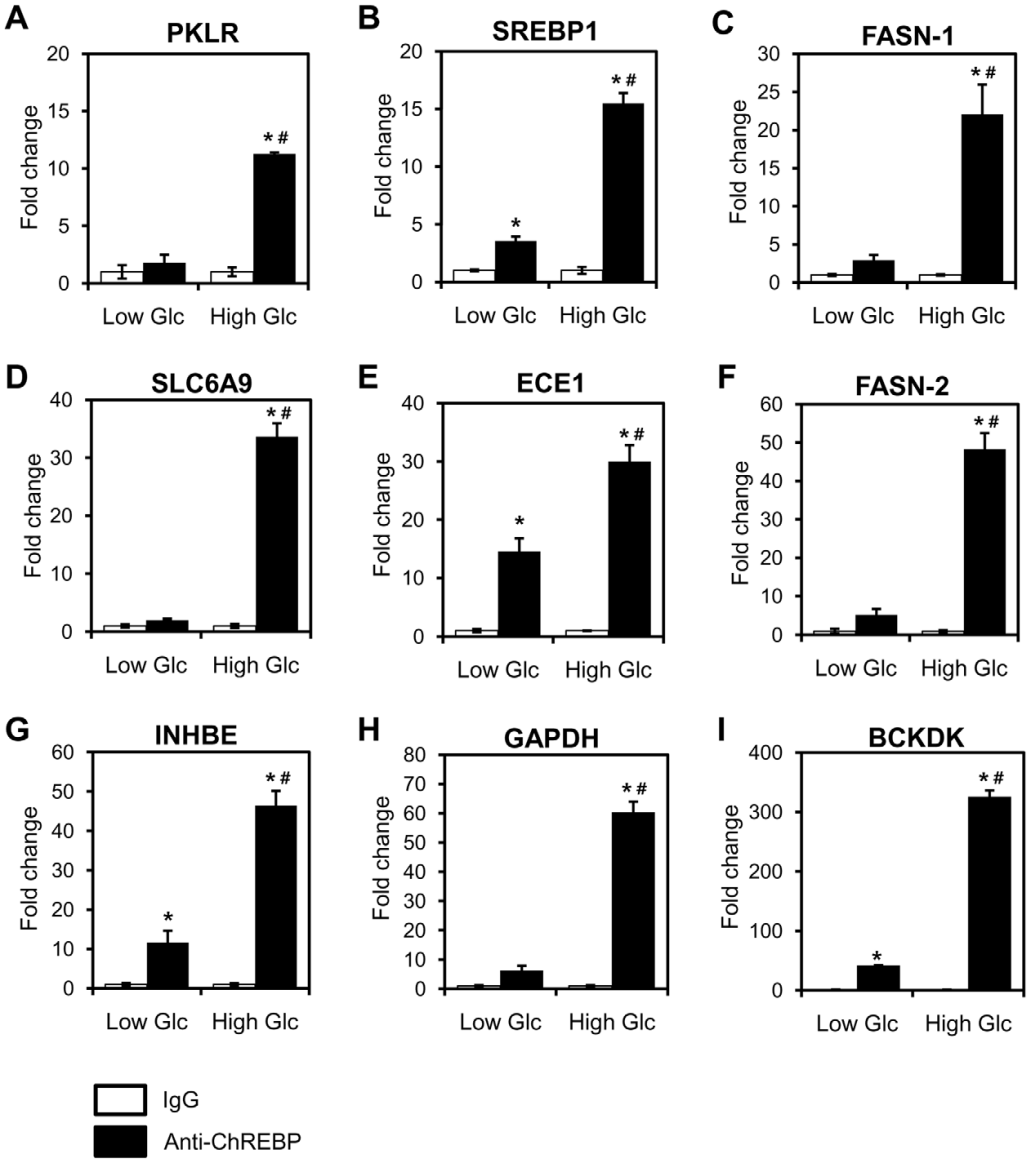
Effects of glucose on ChREBP binding. HepG2 cells were treated with low (2.7 mM) and high (25 mM) glucose for 8 h. Chromatin was isolated and fragmented, and ChIP was performed with control IgG or anti-ChREBP antibody. Validated primers for each gene were used for quantitative real-time PCR. The data presented as fold increase for the signal from anti-ChREBP relative to control IgG. The negative control, Cyclo, showed no enrichment (data not shown). Values represent the mean ± S.D. of three independent samples. *p<0.005 vs. IgG, #*p*<0.0001 vs. 2.7 mM glucose with anti-ChREBP.

ChREBP binding sites of representative ChREBP target genes, PKLR [7], and thioredoxin interacting protein (TXNIP) [18] were presented in Figure 2A and 2B. The vertical axis is the tag count of overlapping mapped sequence reads at each nucleotide position while peaks correspond to regions of DNA putatively bound by ChREBP in HepG2 cells. To validate ChREBP binding sites identified from ChIP-seq analysis, we performed ChIP-qPCR using primers designed specifically for the ChREBP binding sites. We first validated the enrichment of seven peak regions located in six known ChREBP target genes: PKLR, TXNIP, basic helix-loop-helix containing class E40 (bHLHE40) [19], FASN [7], Mid1 interacting protein 1 [20], and glyceraldehyde-3-phosphate dehydrogenase (Figure 2C and S2). ChIP-qPCR analysis confirmed that the ChREBP binding sites are enriched in ChIPed DNA with the greatest enrichment observed at the TXNIP promoter (532-fold). To further validate novel ChREBP binding sites, we randomly selected 37 binding regions of variable peak intensity for ChIP-qPCR (Figure S2). All of the sites showed at least 6-fold enrichment with ChREBP antibodies relative to normal IgG, regardless of their relative locations to genes (promoter, intron, exon, or intergenic region). In addition, the enrichment of ChREBP binding sites in ChIPed DNA was significantly enhanced in high glucose conditions, indicating glucose affects the binding of ChREBP to DNA (Figure 3). Unexpectedly, ChREBP binding sites associated with endothelin converting enzyme 1 (ECE1), inhibin, beta E (INHBE) and branched chain ketoacid dehydrogenase kinase (BCKDK) genes showed more than 10-fold enrichment with ChREBP antibodies relative to normal IgG even under low glucose conditions.

### Identification of *cis*-regulatory motifs in the ChREBP binding sites

**Figure 4:**
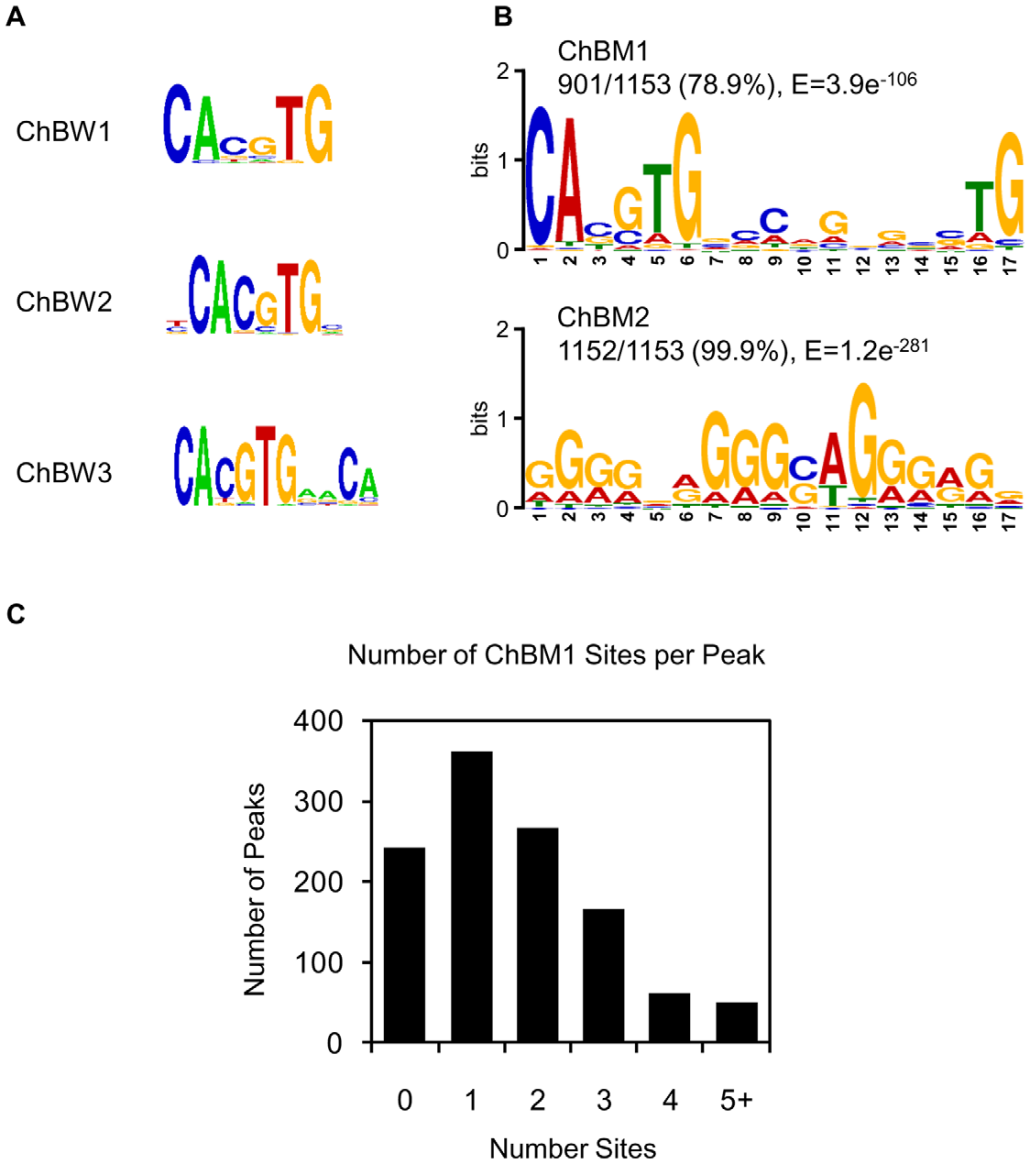
Enriched motifs in ChREBP binding sites in human liver DNA. The 1153 peak regions were analyzed for overrepresented motifs using W-ChIPMotifs (A) and MEME (B). The three and two top-scoring motifs from each analysis are shown. (C) Number of ChBM1 motifs in a peak identified by ChIP-seq (*p*<0.001).

To search for enriched motifs in the peaks from the ChIP-seq dataset, we used the motif finding program W-ChIPMotifs [21] and MEME [22]. Perfect E-boxes (CACGTG, PWM score = 1) and the extended E-boxes (tCACGTGc, PWM score = 1, CACGTGaaCA, PWM score = 0.956), were identified in the ChREBP binding sites with strong enrichment (Figure 4A). Two novel ChREBP binding motifs were identified using MEME (Figure 4B). The first motif, termed as ChREBP binding motif 1 (ChBM1), is present in 901 peaks (78.9%, E = 3.9e^−106^, *p*<0.001). ChBM1 consists of a perfect E-box and an imperfect E-box separated by 5 nt (CABGTG-nnCnG-nGnSTG), resembling the ChoRE consensus sequence (CAYGNG-n5-CNCRTG) [6]. Figure 4C represents the number of ChBM1 sites per peak and approximately 54.6% of the ChREBP binding sites contain one or two ChBM1 motifs. When ChREBP binding sites were scanned with ChBM1 using a more stringent cutoff (*p<*0.0001), ChBM1-derived sequence (CACGTG GCCGG CGCGTG) was found to be present in 39.3% (453/1153, *p<*0.0001) of the 1153 peaks. The second motif, termed as ChREBP binding motif 2 (ChBM2, GGGGNRGGGSAGGGRGN) is present in 1152 peaks (99.9%, E = 1.2e^−281^). Sequence-wise, ChBM2 significantly differs from known ChREBP binding sites. Therefore, rather than being a direct binding site for ChREBP, ChBM2 may represent binding sites for ChREBP–associated proteins. TOMTOM analysis, which compares a novel motif with known DNA-binding motifs in TRANSFAC motif database [22], revealed that ChBM2 is matched to Sp1-binding motif with the highest probability [*p* = 1.2*e* ^−04^, false-discovery rate (FDR) = 0.042].

**Figure 5:**
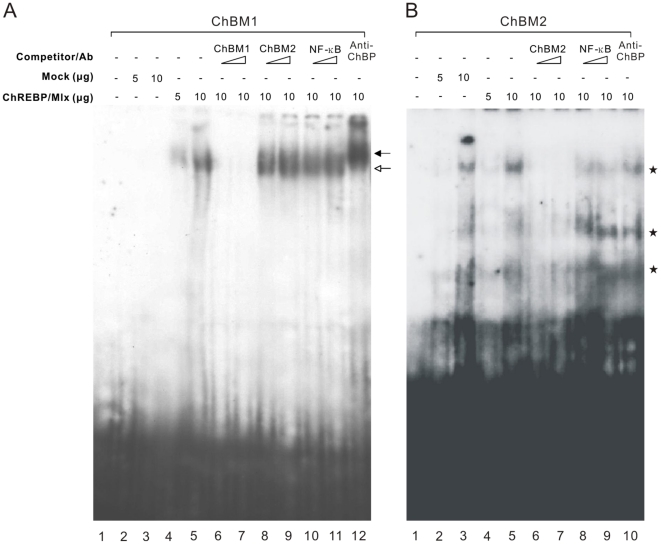
Validation of ChREBP/Mlx binding to two enriched motifs, ChBM1 and ChBM2. Electrophoretic mobility shift assays were performed with an oligonucleotide containing the ChBM1 (A) or ChBM2 (B) probe. All lanes contain the labeled probe, and lanes 2–12 contain 5 or 10 µg of HEK293 nuclear extract. Lanes 2 and 3 are HEK293 mock-transfected nuclear extract. The other lanes contain extract from HEK293 cells transfected with the ChREBP and Mlx expression plasmids. For competition assays, a 10- or 50-molar excess of various unlabeled competitor DNAs was added to the reaction mixture. Anti-ChREBP (Anti-ChBP, 0.6 µg) was added as indicated. The *white arrow* indicates the position of the ChREBP/Mlx complex. The *black arrow* indicates the position of the antibody-supershifted complexes. The *asterisks* indicate the position of background bands present in the control HEK293 cell nuclear extract.

To evaluate whether these two motifs, ChBM1 and ChBM2, were *bona fide* binding sites of ChREBP, we performed electrophoretic mobility shift assay (EMSA). HEK293 cells were transfected with expression vectors of ChREBP and heterodimer partner Mlx and nuclear extracts were prepared. Nuclear extracts were incubated with a radiolabeled oligonucleotide containing ChBM1 or ChBM2 sequence (Figure 5). The results showed that ChREBP/Mlx specifically binds to the ChBM1, but not to ChBM2. The binding of ChREBP/Mlx to ChBM1 was competitively inhibited by an excess of the corresponding unlabeled wild type ChBM1 oligonucleotide (Figure 5A, lane 6 and 7), but not by adding the ChBM2 and nuclear factor kappa B (NF-κB) (Figure 5A, lanes 8–11). The specificity of the ChREBP binding to ChBM1 was further confirmed by performing super-shift assay using antibodies that recognize ChREBP, as shown in Figure 5A, lane 12. As for ChBM2, no significant band shift was observed in comparison to that with vector nuclear lysates (Figure 5B, lanes 2–5). In addition, adding ChREBP-specific antibody had no effect on the migration of the band (Figure 5B, lane 10). Collectively, these findings indicate that ChREBP/Mlx binds to ChBM1, not to ChBM2, which reconciles with the predictions from motif analysis.

### Pathway analysis of ChREBP targets genes

**Figure 6:**
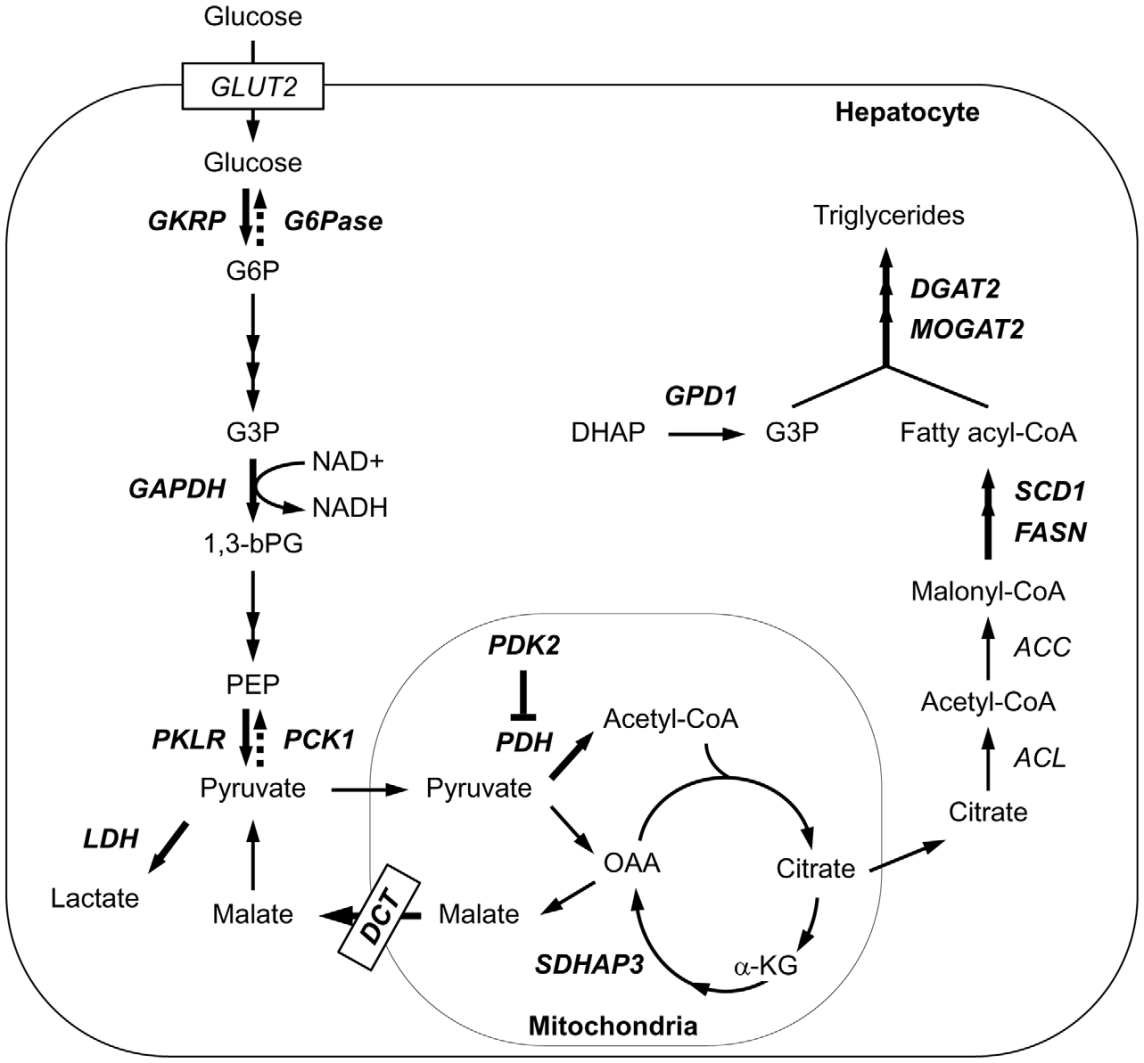
Genes identified as ChREBP targets in *De novo* lipogenesis pathway. A schematic of the *de novo* lipogenesis pathway is shown. Direct targets of ChREBP identified by ChIP-seq are indicated in *boldface* type. GKRP, glucokinase regulatory protein; G6Pase, glucose-6-phosphatase, catalytic subunit; GAPDH, glyceraldehyde-3-phosphate dehydrogenase; PKLR, pyruvate kinase, liver and RBC; PCK1, phosphoenolpyruvate carboxykinase1; LDH, lactate dehydrogenase A; DCT, dicarboxylate transporter; PDK2, pyruvate dehydrogenase kinase isozyme2; PDH, pyruvate dehydrogenase; SDHAP3, succinate dehydrogenase complex, subunit A; FASN, fatty acid synthase; SCD1, stearoyl-CoA desaturase 1; GPD1, glycerol-3-phosphate dehydrogenase 1 (soluble); MOGAT2, monoacylglycerol O-acyltransferase 2; DGAT2, diacylglycerol O-acyltransferase homolog 2.

To investigate the functional relationship of 783 ChREBP target genes, the database for Annotation and Visualization, and the Integrated Discovery was used [23]. The pathways significantly enriched [*p*-value<0.05] for ChREBP target genes were summarized in Table 1. The pathway for lipid, fatty acid and steroid metabolism is most significantly enriched by 48 novel ChREBP target genes (*p* = 1.03*e* ^−4^), supporting the central role of ChREBP in lipid metabolism. Further investigation of ChREBP target genes involved in this pathway identified the key enzymes of gluconeogenic pathway, such as phosphoenolpyruvate carboxykinase (PEPCK) and glucose-6-phosphatase catalytic subunit (G6Pase). This result indicates that ChREBP may work as a dual regulator of lipogenesis and gluconeogenesis, which have a reciprocal relationship in response to glucose (Figure 6).

**Table 1:** Analysis of the nearest gene dataset by the DAVID gene ontology program.

Panther Category (Biological Processes)	Count	*p*-Value	FDR
BP00019:Lipid, fatty acid and steroid metabolism	53	1.03E-04	0.128
BP00141:Transport	73	2.59E-03	3.175
BP00287:Cell motility	26	3.00E-03	3.666
BP00063:Protein modification	60	2.61E-02	28.050
BP00027:Regulation of lipid, fatty acid and steroid metabolism	5	2.69E-02	28.788
BP00099:Phosphate transport	4	2.91E-02	30.689
BP00028:Lipid and fatty acid transport	11	3.04E-02	31.909
BP00285:Cell structure and motility	58	3.30E-02	34.070
BP00167:Synaptic transmission	19	3.73E-02	37.657
BP00067:Protein glycosylation	14	3.86E-02	38.671
BP00248:Mesoderm development	32	4.57E-02	44.112
BP00013:Amino acid metabolism	16	4.58E-02	44.183
BP00272:Phospholipid metabolism	12	4.59E-02	44.226

In addition, ChREBP target genes are associated with five functional annotation clusters with enrichment scores above 2, including protein dimerization, enzyme regulator activities, embryonic development, carbohydrate metabolic process, and lipid metabolism, suggesting broader roles of ChREBP in other biologically important processes (Table S2).

### Expression profiling of glucose-responsive genes and their relationship with ChREBP target genes

To determine whether ChREBP binding to its target genes change their expression levels, we performed gene expression profiling analysis. Since the activity of ChREBP is regulated by glucose, we compared gene expression profiles of RNA isolated from HepG2 cells cultured under either no- or high-glucose conditions (in the same manner as cells used for ChIP-seq analysis). The comparison of expression profiles between no- and high-glucose conditions revealed that expression levels of 1822 genes were significantly changed by high glucose.

**Figure 7:**
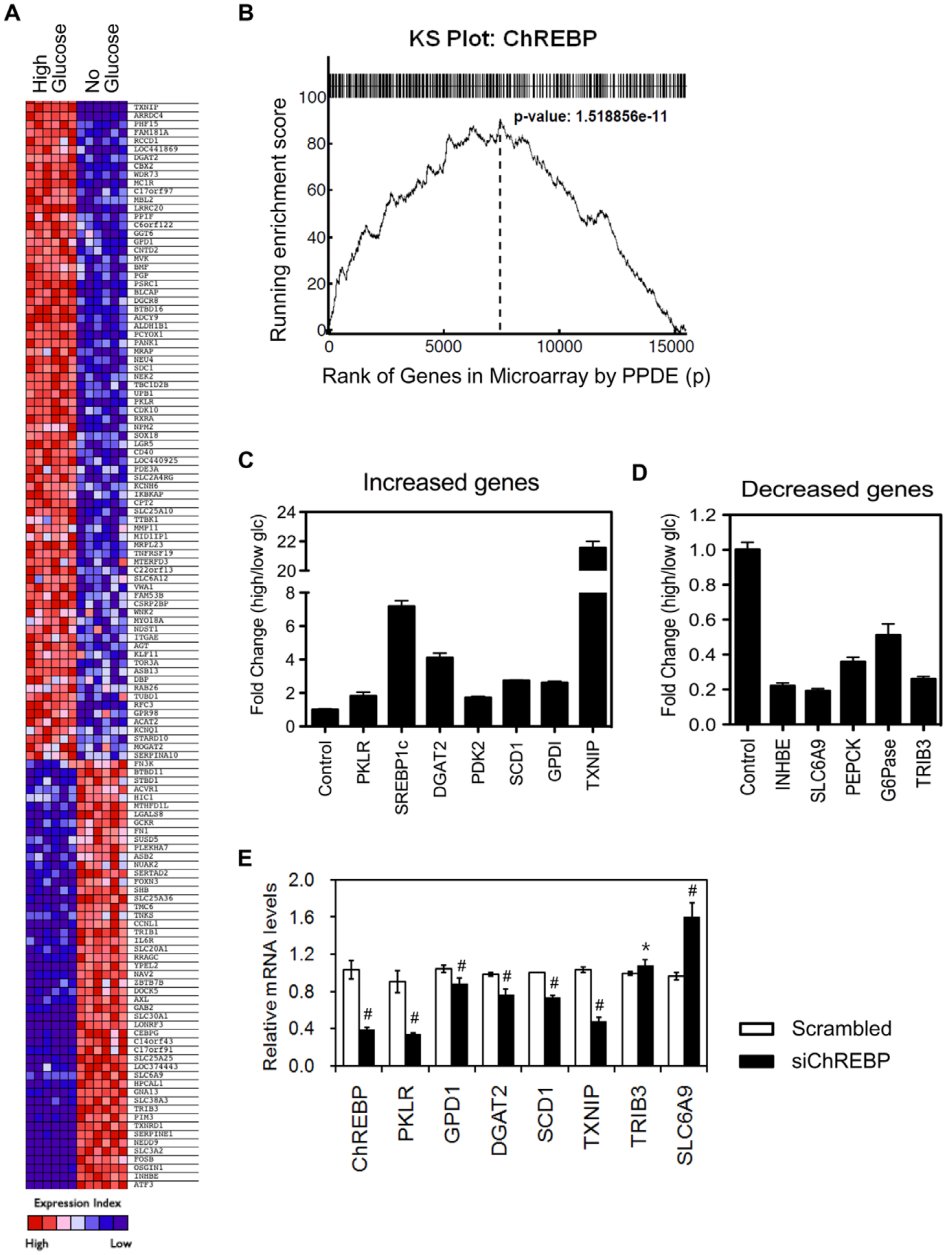
Correlation of ChREBP binding with gene expression. (A) Heat map view of a sample of ChREBP target genes exhibiting greater than two-fold expression changes in the high glucose state. (B) KS plot. The ChIP-seq peaks were analyzed for their representation within an expression array dataset from no vs. high glucose-treated HepG2 cells as described in the text. All genes in the microarray were ranked by posterior probability of differential expression (PPDE) on the *x*-axis and the graph plots by the running enrichment score. (C, D) Experimental validation of microarray results of 12 selected ChREBP target genes. HepG2 cells were incubated under 2.7 mM glucose conditions for 16 h. Cells were then either kept in 0 or 25 mM glucose medium for 8 h and harvested for RNA preparation. The levels of target genes were determined by qRT-PCR. Expression levels were normalized to expression of cyclophilin and mRNA levels in no glucose treated cells were set to 1. Values represent the mean of triplicate samples ± S.D. (E) Effects of ChREBP gene silencing on the expression of ChREBP target genes. HepG2 cells were transfected with 20 nmol of either ChREBP siRNA or scrambled siRNA and incubated for 40 h in 2.7 mM DMEM. Then the cells were cultured in 25 mM glucose. After 8 h, total RNA was extracted and analyzed for the expression of ChREBP target genes by qRT-PCR. Data represent the mean ± S.D. of three independent transfections.

To compare the list of ChREBP target genes from the ChIP-seq dataset with the gene expression profiling dataset, the expression profiling dataset was rank-ordered by fold change such that the most highly up-regulated genes under the high glucose condition were at the top of the ranked list (Figure 7A). Interestingly, out of 109 ChREBP target genes are differentially expressed under the high-glucose condition, 47% (51 genes) are up-regulated, and the other 53% (58 genes) down-regulated. These observations indicate that ChREBP functions as a transcriptional repressor as well as an activator. Next, we ranked the list of genes by significance of differential expression under the high glucose conditions and then determined how the genes containing ChREBP binding sites were distributed over the expression list using the modified Kolmogorov–Smirnov (KS) test [24]. The analysis showed a highly significant running enrichment score because the genes identified by ChIP-seq were preferentially located toward the top of the differentially expressed gene list (Figure 7B; *p* = 1.5*e* ^−11^). Thus, the distinct possibility exists that the ChIP-seq identified sites correspond to functional sites of ChREBP action.

To further validate the relationship between ChREBP binding and glucose-dependent gene regulation, the expression levels of several genes including PKLR, TXNIP, sterol regulatory element binding protein-1c (SREBP-1c), diacylglycerol O-acyltransferase homolog 2 (DGAT2), pyruvate dehydrogenase kinase isozyme 2, INHBE, PEPCK, and solute carrier family 6 member 9 (SLC6A9) were determined by reverse transcription/quantitative real-time PCR (qRT-PCR). The binding of ChREBP to these genes was already confirmed by ChIP-qPCR (Figure 3 and S2). Despite ChREBP binding to all these genes, some genes are up-regulated by glucose and others repressed (Figure 7C and 7D). Interestingly, insulin responsive transcription factor SREBP-1c mRNA was dramatically increased by high glucose (7-fold), suggesting that ChREBP binds to SREBP-1c gene, transcriptionally activating its expression in HepG2 cells. Moreover, PEPCK and G6Pase, two rate-limiting enzymes of gluconeogenesis, were decreased in response to high glucose (0.4-fold and 0.5-fold, respectively). To determine the direct implication of ChREBP in glucose action on expression of ChREBP target genes, we next silenced ChREBP in HepG2 cells using short interfering RNAs (siRNAs). As shown in Figure 7E, transfection of scrambled siRNAs did not affect the expression of ChREBP, while ChREBP-specific siRNAs suppressed ChREBP expression. Silencing of ChREBP in HepG2 cells reduced the expression of PKLR, DGAT2, stearoyl-CoA desaturase 1 (SCD1), and TXNIP, but induced that of tribbles homolog 3 (TRIB3) and SLC6A9. Our results show that the majority of the ChREBP binding sites identified by the ChIP-seq analysis are functionally important in mediating glucose-responsive gene expression. In addition, our results indicate that ChREBP may act as either an activator or a repressor within the same biological context (e.g., as an activator of lipogenesis and as a repressor of gluconeogenesis).

## Discussion

The aim of the present study was to identify the direct targets of ChREBP on a genome-wide scale and to understand the underlying mechanism of glucose-responsive gene regulation by ChREBP. ChIP-seq analysis revealed that ChREBP binds to 1153 sites across the genome, 45% of which are located in potential gene regulatory regions including proximal/distal promoters, 5′ UTRs, and the first/second introns of known genes. In addition, the present study showed that the majority of the ChREBP binding sites identified by the ChIP-seq analysis are functionally important in mediating glucose-responsive gene expression with ChREBP acting as either an activator or a repressor.

### ChREBP binding sites and motifs

As mentioned above, 45% of ChREBP binding sites are closely associated with TSSs, yet the rest are remotely associated with TSSs. Previous genome-wide localization analysis of other transcription factors such as Foxa2, ERα, Myc, and PPARγ/RXR also revealed that transcription factor binding sites can be localized to regions other than the 5′-proximal promoter regions, such as far upstream sequences, introns, and 3′ region of the gene [25], [26], [27], [28]. Whether these remotely associated sites may function as enhancers or locus control regions (LCRs) is yet to be discovered.

ChREBP forms a heterodimer with Mlx to bind to ChoRE. Ma and colleagues [6] identified the consensus sequence of the ChoRE as CAYGNG-n5-CNCRTG by analyzing functional ChoRE sequences from seven different genes. According to the authors, the heterotetramer formation between ChREBP/Mlx heterodimers is essential to stabilize their binding to the tandem E box-like sites (two imperfect CACGTG motifs) in ChoRE. In addition, the stringent spacing between E boxes within ChoRE is critical for the transcriptional response to glucose [29]. Consistent with these previous observations, a refined sequence motif (ChBM1, CABGTG-nnCnG-nGnSTG), similar to the ChoRE consensus sequence, was predicted from ChREBP binding sites identified in the present study. When ChREBP binding sites were scanned with ChBM1 for further refinement of the consensus sequence using a more stringent cut-off (*p<*0.0001), a new ChBM1-derived sequence (CACGTG GCCGG CGCGTG) was acquired as a consensus sequence, which is present in 39.3% (453/1153, *p<*0.0001) of the 1153 peaks. The overall structure of this ChBM1-derived sequence is precisely matched to that of ChoRE consensus sequence, but with more details.

ChBM2 (GGGGNRGGGSAGGGRGN), one of the major binding motifs, significantly differs from known ChREBP binding sites and no direct ChREBP binding to ChBM2 was observed in EMSA (Figure 5B). This may result from the increased cross-links between ChREBP and other proteins by relatively long fixation time, although we optimized the cross-linking condition using two parameters to judge the best cross-linking condition: average size of DNA after shearing and analysis of the immunoprecipitation efficiency by qPCR (PLKR was used as a positive control). It is also possible that ChREBP and additional transcription factor may function together to regulate gene expression. In fact, the regulatory effect of ChREBP seems to depend on transcriptional modules made up of ChREBP binding elements and other transcription factor binding sites. Recently, a study showed that ChREBP can cooperate with nuclear factor Y (NF-Y) to mediate induction of TXNIP expression by glucose or adenosine-containing molecules [30]. Our motif analysis revealed that ChBM2 is matched to the Sp1 binding motif. Although no link has been reported between ChREBP and Sp1, several reports have suggested that Sp1, a ubiquitously expressed protein, may be involved in glucose responsiveness. Members of the Sp1 family have been proposed as being required for glucose-dependent induction of the plasminogen activator inhibitor 1 [31], transforming growth factor β [32] in vascular muscle cells and expression of neuronal vesicular glutamate transporter isoform2 in pancreatic β-cells [33]. Further mutagenesis and functional studies are necessary to demonstrate clearly that ChBM2, a ChREBP-associated motif, is a co-regulatory motif.

### Correlation between ChREBP target genes and their expression

Gene set enrichment analysis using the modified KS test revealed a high degree of correlation between ChREBP binding and ChREBP-dependent gene expression when HepG2 cells are subjected to high glucose conditions (*p* = 1.5*e* ^−11^). Although many ChREBP target genes were upregulated as expected given the known role of ChREBP in activating glycolytic and lipogenic genes, especially in the liver [1], [5], [7], [34], some ChREBP target genes were downregulated. The repressive effects of ChREBP on glucose-mediated gene expression was further validated by qRT-PCR experiments showing decreased expression levels of genes such as PEPCK, G6Pase, INHBE, SLC6A9, and TRIB3. Interestingly, PEPCK and G6Pase are rate-limiting enzymes of gluconeogenesis, indicating that ChREBP may acts as either an activator or a repressor within the same biological context (e.g., as an activator of lipogenesis and as a repressor of gluconeogenesis). This observation reconciles with the study of Cairo and colleagues [2] that ChREBP may repress transcription of target genes, depending on the context and/or organization of E-box target sites in cell culture systems. In addition, ChREBP was recently shown to repress ARNT/HIF-1β in pancreatic β-cells [35], which is the first *in vivo* evidence that ChREBP may play a repressive role in glucose responsive transcription.

### Functional clustering of ChREBP target genes

According to GO analysis, the functional group for lipid, fatty acid and steroid metabolism was enriched by ChREBP target genes in human liver cells. This observation is consistent with the previous gene expression profiling using rodent hepatocytes and liver. ChREBP null mice displayed lower mRNA levels of several lipogenic enzymes, such as PKLR, FASN and SCD1, compared with wild type mice [36]. In addition, Ma and colleagues [6] showed that ChREBP activates the entire program of *de novo* lipogenesis by gene profiling in the presence or absence of a dominant-negative Mlx in rat hepatocytes cultured in high glucose conditions. One of the identified ChREBP target genes, SREBP-1c, is a key transcription factor in lipid metabolism [37]. We have previously demonstrated that the expression of SREBP-1c gene is increased by glucose as well as insulin in mouse hepatocytes [38]. Interestingly, our ChIP-seq analysis and subsequent validation studies revealed that ChREBP binds to the human SREBP-1c gene. These results indicate that ChREBP is a mediator of glucose action on SREBP-1c gene expression.

We also found that ChREBP target genes are functionally related to cell motility. Although there is not known experimental evidence regarding the role of ChREBP in cell migration, it is shown that MondoA, a ChREBP paralog, regulates a number of genes such as collagen type IV and fibulin 2, which are components of ECM [39]. HepG2 cells are proliferative and transformed cells whereas hepatocytes in the liver are largely arrested in the G0 phase of the cell cycle. Therefore, enrichment of ChREBP target genes in cell motility may be related to the proliferative status of HepG2 cells. In this regard, Tong and colleagues [40] have reported that the induction of ChREBP in response to mitogenic signals is required for cell proliferation. Our characterization of ChREBP binding profiles across the entire human genome in HepG2 cells provides novel and important insight into the regulation of ChREBP target gene network. Although HepG2 cells have shown to express a wide range of liver-specific functions as a reproducible human system, it is known that some genes such as liver-specific transcription factors (hepatocyte nuclear factors and C/EBPα) and drug metabolizing enzymes are differently expressed in HepG2 cells compared to hepatocytes in the liver [41]. Due to the limitation of the immortalized cell system, further *in vivo* studies are required to fully elucidate ChREBP regulated transcription in hepatocytes in the liver.

## Materials and Methods

### Cell culture

Human hepatocellular carcinoma HepG2 and human embryonic kidney (HEK) 293 cells were obtained from the American Type Culture Collection (ATCC, Manassas, VA, USA) and maintained in 25 mM glucose Dulbecco's modified Eagle's medium (DMEM) supplemented with 10% fetal bovine serum (FBS) and 100 U/ml penicillin/streptomycin. For ChIP-seq and gene expression profiling, HepG2 cells (passage 8–10) were cultured in 2.7 mM glucose DMEM with 10% FBS for 16 h and then cultured in 0, 2.7, or 25 mM glucose medium for an additional 8 h.

To obtain nuclear extracts for gel shift assays, HEK293 cells were transfected in 100 mm plates using Lipofectamine (Invitrogen) according to the manufacturer's directions. Ten µg each of expression plasmids of ChREBP and Mlx were cotransfected. Nuclear extracts were prepared from cells 48 h post-transfection using the ‘nuclear cell extract’ protocol in the Nuclear Extract kit from ActiveMotif (Carlsbad. CA).

### Chromatin immunoprecipitation-quantitative real-time PCR (ChIP-qPCR)

ChIP assays were performed as previously described [42] with minor modification. Briefly, HepG2 cells (1×10^7^ cells) previously cultured in 2.7 mM or 25 mM glucose for 8 h were cross-linked with 1% formaldehyde for 30 min at 37°C. The cross-linking time was optimized using two parameters: average size of DNA after shearing and analysis of the immunoprecipitation efficiency by PCR. Cross-linking was stopped by the addition of glycine to a final concentration of 0.125 M. The cells were washed three times with ice-cold phosphate-buffered saline and kept on ice for 10 min in 25 mM HEPES (pH 7.8), 1.5 mM MgCl_2_, 10 mM KCl, 0.1% Nonidet P-40, 1 mM dithiothreitol, 0.5 mM phenylmethylsulfonyl fluoride, and protease inhibitor cocktail (Roche, Basel, Switzerland). Nuclei were collected and resuspended in sonication buffer for 30 min on ice and sonicated on ice to an average length of 200 bp using a tissue lyser set at 50% amplitude. After sonication, the chromatin solution (500 µg) was incubated with Dynabeads protein A (100.01D; Invitrogen, Carlsbad, CA, USA) and 5 µg of rabbit anti-ChREBP (NB400-135; Novus, St. Louis, MO, USA) or 5 µg of rabbit normal IgG (sc-2027; Santa Cruz Biotechnology, Santa Cruz, CA, USA) at 4°C overnight. Antibody-bound complexes were obtained and DNA fragments extricated from these complexes were purified using a QIAquick PCR Purification Kit (Qiagen, Shanghai, China). The purified ChIPed DNA samples were analyzed by qPCR using an Applied Biosystems Prism 7900HT sequence detection system (Applied Biosystems, Carlsbad, CA, USA) and SYBR-Green chemistry as described [43]. The qPCR oligonucleotide pairs for the human promoters were validated by analysis of template titration and dissociation curves and provided in Table S3.

### Sequencing and data analysis

For ChIP-seq library construction, ChIPed DNA was end-repaired, ligated to adaptors, and amplified. Amplified fragments of about 300 bp (with adaptors) were gel purified from 2% agarose and used for sequencing on GAII (Illumina Inc., San Diego, CA, USA) according to the manufacturer's instructions. Sequence reads were mapped to the human reference genome using MAQ [44] and bowtie [45], and sequence reads that mapped to multiple sites in the genome were removed. To identify ChREBP binding regions, PeakFinder [14] and CisGenome [15] were used for data analysis. In defining ChREBP binding peaks, we used stringent settings, with the minimum sequence reads set to 8 and the minimum read ratio comparing ChREBP with input set to 5.

### Annotation of genes and GO analysis

All ChREBP binding sites were assigned to nearest genes based on the Homo sapiens NCBI Build 36.1 genome assembly (NCBI36/hg18; March 2006). GO analysis for ChREBP target genes was conducted using the NIH Database for Annotation, Visualization, and Integrated Discovery (DAVID; http://david.abcc.ncifcrf.gov/) [16]. The ChREBP target genes were classified into functionally related gene groups using ‘PANTHER Biological Process’ term.

### Motif analysis

We applied the motif-finding program W-ChIPMotifs [21] and MEME [22] to ChREBP target peak regions to search for statistically overrepresented consensus ChREBP binding sites in the peak regions. Motifs were presented as position-dependent letter-probability matrices.

### Electrophoretic mobility shift assays (EMSA)

Probes for the EMSA were labeled with [γ-^32^P]ATP using T4 polynucleotide kinase. Labeled double stranded oligonucleotides were prepared by mixing a 5-fold molar excess of the complementary single-stranded DNAs in 50 mM NaCl, heating to 95°C for 5 min, and then cooling to room temperature. The oligonucleotides used in these assays were as follows: ChBM1, 5′-TCG AGG CAC GTG GCC AGG GCC TGA TCG A-3′; ChBM2, 5′-TCG ACC GGG GAA GGG CAG GGA GGG GTC GA-3′; NF-κB, 5′-AGT TGA GGG GAC TTT CCC AGG C-3′.

The labeled probe (100,000 cpm) was combined with 5–10 µg of nuclear extract from HEK293 cells in 75 mM of KCl, 7.5% (v/v) of glycerol, 20 mM of HEPES (pH 7.4), 0.1% (v/v) of Nonidet P-40, and 2 mM of dithiothreitol. The nonspecific competitors, 2 µg of poly dI-dC (Amersham Biosciences) and 20 pM of nonspecific antisense oligonucleotide were added in each binding reaction. Following incubation with the labeled probe for 30 min on ice, samples were subjected to electrophoresis on a 4.5% nondenaturing polyacrylamide gel. For reactions with antibodies, nuclear extract was preincubated with antibody for 30 min on ice before addition of the probe. For competition assays, a 10- or 50-molar excess of various unlabeled competitor DNAs was added to the reaction mixture prior to the addition of the probe. Results were visualized by autoradiography.

### Expression profiling (microarray) and data analysis

Total RNA from HepG2 cells was prepared using a Qiagen RNeasy Kit. Total RNA from six biological replicates under 0 or 25 mM glucose conditions were then amplified into cRNA and biotinylated by *in vitro* transcription using the Illumina total prep RNA amplification kit (Ambion, Foster City, CA, USA) according to the manufacturer's instructions. Biotinylated cRNAs were then hybridized to an Illumina HumanHT-12 BeadChip. Microarray data were normalized and assessed for differential expression using the LIMMA component (Linear Models for Microarray Data) of the Bioconductor package [46] and the CyberT analysis program [47]. The array data were submitted to Gene Expression Omnibus (GEO, http://www.ncbi.nlm.nih.gov/geo/query/acc.cgi) under accession number GSE22074.

### Kolmogorov–Smirnov (KS) analysis

The ChIP-seq data were compared with expression microarray data using a KS plot, which is a modified method of gene set enrichment analysis (GSEA) [24]. A KS plot can determine whether our gene list from ChREBP ChIP-seq tends to occur toward the top or bottom of the ranked gene list of ChREBP expression microarray data. The genes in gene expression microarray were rank-ordered by the posterior probability of differential expression (PPDE) and tested for matches between genes in the expression microarray and the ChREBP ChIP-seq dataset. A KS plot was obtained by calculating enrichment score, which was determined by increasing a running sum if a match occurred, or by decreasing it if no match was found.

### Silencing of endogenous human ChREBP

HepG2 cells were transfected with siRNAs using RNAiMAX according to the manufacturer's directions (Invitrogen). A scrambled siRNA (Stealth RNAi Negative Control Med GC) was purchased from invitrogen and a siRNA for human ChREBP (sense, 5′-GGUAU AUCCA GUAUG UGAAU U-3′; antisense, 5-UUCAC AUACU GGAUA UACCU U-3′) was purchased from Genolution (Seoul, Republic of Korea). Briefly, 1×10^5^ cells, 20 nM of RNAi medium, and 20 nmol of each ChREBP siRNA or scrambled RNA incubated in serum reduced OPTI-MEM for 10 min and were plated in 12-well plates. The cells were cultured in 2.7 mM glucose DMEM for 40 h and then cultured in 25 mM glucose medium for an additional 8 h. The cells were lysed and total RNA was extracted.

### RNA measurement (Reverse transcription/quantitative real-time PCR, qRT-PCR)

Total RNA was isolated from cultured cells using TRIzol reagent (Invitrogen, Carlsbad, CA, USA). Two micrograms of total RNA was treated with RNase-free DNase (Roche) and reverse transcribed using SuperScript II (Invitrogen) and random hexamers. Gene-specific primers were designed using Primer Express Software (PerkinElmer Life Sciences, Waltham, MA, USA) and validated by analysis of template titration and dissociation curves. Primer sequences are provided in Table S3. qRT-PCR was performed using an Applied Biosystems Prism 7900HT sequence detection system and SYBR-Green chemistry as described [43]. Ten-microliter qRT-PCR reactions contained 25 ng of reverse-transcribed RNA, each primer (150 nM), and 5 µl of 2× SYBR Green PCR master mix (Applied Biosystems). Results of qRT-PCR were evaluated by the comparative Ct method (User Bulletin No. 2; PerkinElmer Life Sciences) using cyclophilin as the invariant control gene. All samples were analyzed in triplicate and expressed as the mean ± S.D.

## Supporting Information

Figure S1 Determination of the optimal condition for ChREBP activation in HepG2 cells. HepG2 cells were cultured in 2.7 mM glucose DMEM for 16 h and then cultured in high (25 mM) glucose medium for an indicated time. Total RNA was extracted and the PKLR and FAS gene expression was determined by qRT-PCR. Expression levels were normalized to expression of cyclophilin. Values represent the mean of triplicate samples ± S.D.
(TIF)

Figure S2 Validation of ChIP-seq binding sites by single gene ChIP-qPCR. ChIP-qPCR was performed on ChREBP-enriched chromatin prepared from high glucose-treated HepG2 cells. All randomly selected sites were validated by single gene ChIP-qPCR. Positive control region (PKLR) and negative controls (PK-up, 4 kb upstream region of PKLR and Cyclo, cyclophilin exon) were included.
(TIF)

Table S1 The peak location and the nearest gene list. The nearest genes to the ChREBP binding peaks are listed.
(PDF)

Table S2 Functional annotation clustering of the ChREBP ChIP-seq dataset. To reduce redundancy, the newly developed functional annotation clustering report groups and displays similar annotations together to make the biology clearer and more focused for reading when compared to a traditional chart report. For annotation clustering, we used GOTERM_BP_ALL, GOTERM_MF_ALL, PANTHER_BP_ALL, and PANTHER_MF_ALL.
(PDF)

Table S3 Primer sequences for the measurement of human DNA or RNA using an ABI Prism 7900HT system. All primers are listed in the 5′ to 3′ orientation.
(PDF)
