# Supplementary figures and images for: Integrated Expression Profiling and Genome-Wide Analysis of ChREBP Targets Reveals the Dual Role for ChREBP in Glucose-Regulated Gene Expression
Source: PLoS One. 2011 Jul 21;6(7):e22544. doi: 10.1371/journal.pone.0022544 (PMC3141076; doi:10.1371/journal.pone.0022544)

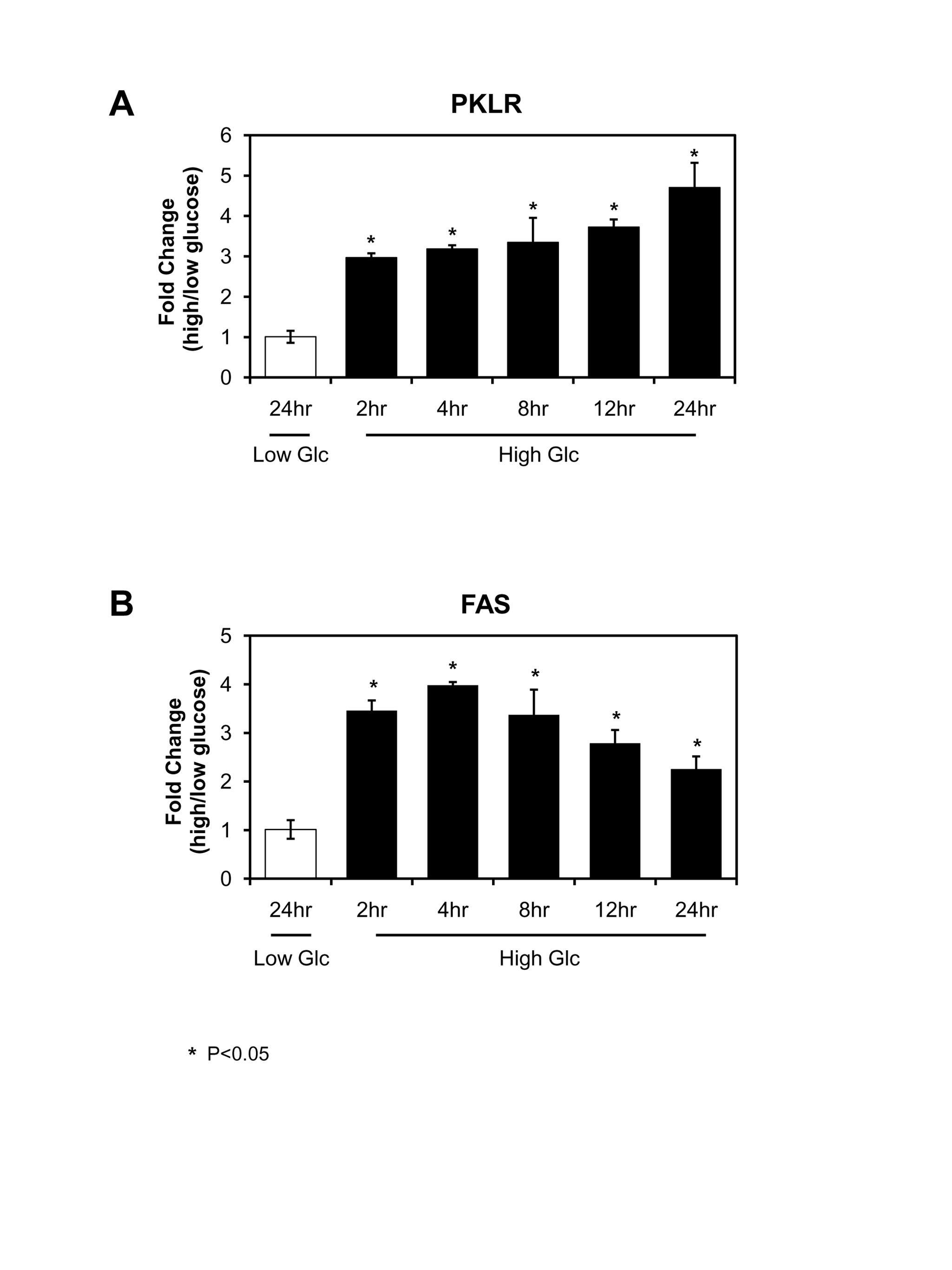

Supplement: Figure S1 — Determination of the optimal condition for ChREBP activation in HepG2 cells. HepG2 cells were cultured in 2.7 mM glucose DMEM for 16 h and then cultured in high (25 mM) glucose medium for an indicated time. Total RNA was extracted and the PKLR and FAS gene expression was determined by qRT-PCR. Expression levels were normalized to expression of cyclophilin. Values represent the mean of triplicate samples ± S.D. (TIF) [file pone.0022544.s001.tif]

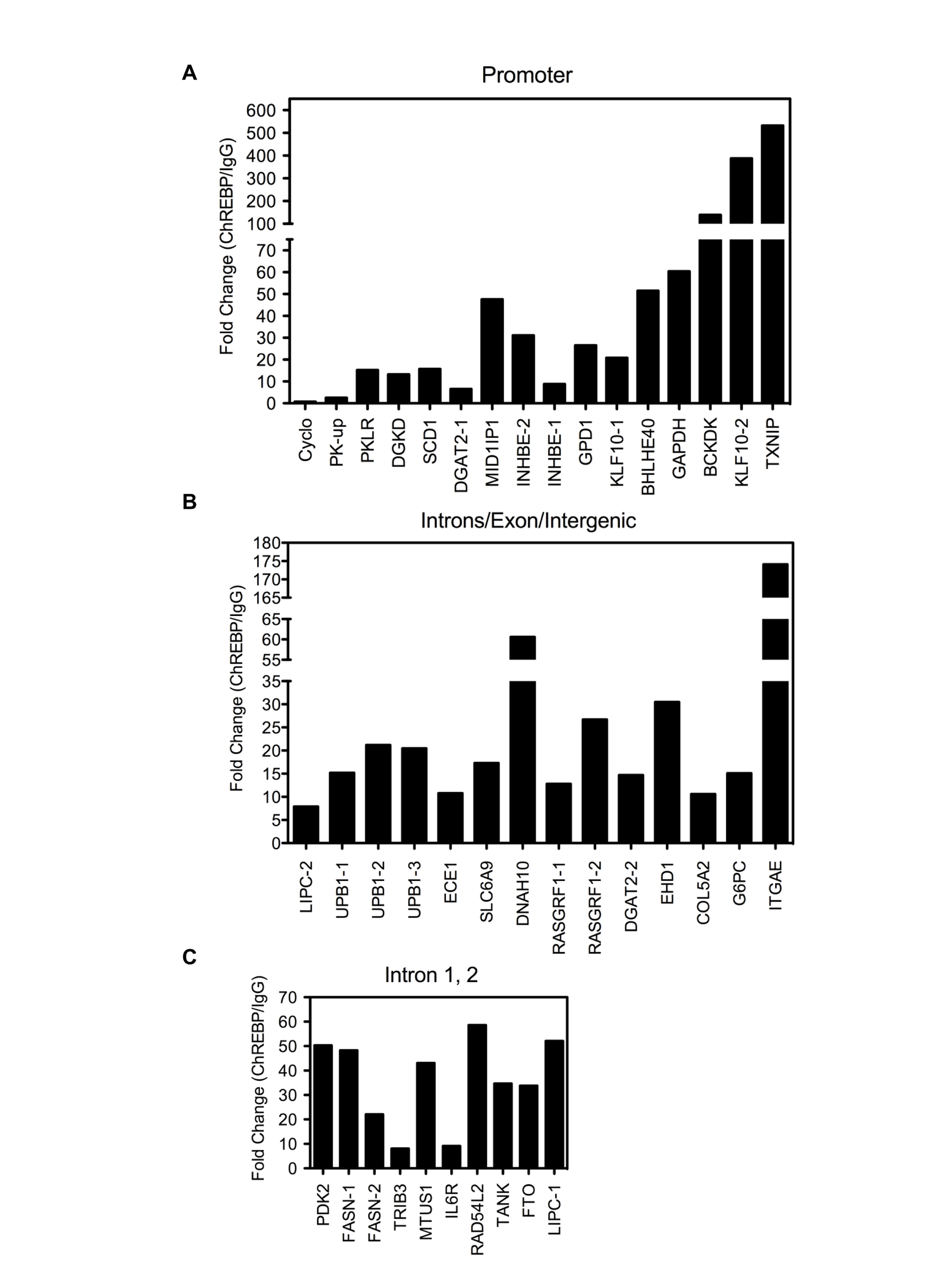

Supplement: Figure S2 — Validation of ChIP-seq binding sites by single gene ChIP-qPCR. ChIP-qPCR was performed on ChREBP-enriched chromatin prepared from high glucose-treated HepG2 cells. All randomly selected sites were validated by single gene ChIP-qPCR. Positive control region (PKLR) and negative controls (PK-up, 4 kb upstream region of PKLR and Cyclo, cyclophilin exon) were included. (TIF) [file pone.0022544.s002.tif]
