# Supplementary material for: Integrated Expression Profiling and Genome-Wide Analysis of ChREBP Targets Reveals the Dual Role for ChREBP in Glucose-Regulated Gene Expression
Source: PLoS One. 2011 Jul 21;6(7):e22544. doi: 10.1371/journal.pone.0022544 (PMC3141076; doi:10.1371/journal.pone.0022544)
Supplement: Table S1 — The peak location and the nearest gene list. The nearest genes to the ChREBP binding peaks are listed. (PDF) [file pone.0022544.s003.pdf]

Table S1. The peak location and the nearest gene list

#ID	chrom	start	stop	nearGeneID	distance	nearGene Symbol	Description
lys1	chr1	1326113	1326409	NR_015434	1342	LOC148413	-
lys2	chr1	1346399	1346907	NM_001145210	0	LOC441869	-
lys3	chr1	1366205	1366704	NM_199121	5435	VWA1	von Willebrand factor A domain containing 1
lys4	chr1	1863284	1863806	NM_001003808	14057	C1orf222	chromosome 1 open reading frame 222
lys5	chr1	2155750	2156349	NM_003036	5758	SKI	v-ski sarcoma viral oncogene homolog (avian)
lys6	chr1	2252267	2252475	NR_024489	-21485	LOC100129534	-
lys7	chr1	3360703	3361019	NM_014448	0	ARHGEF16	Rho guanine nucleotide exchange factor (GEF) 16
lys8	chr1	5931971	5932340	NM_015102	-42778	NPHP4	nephronophthisis 4
lys9	chr1	6008658	6009073	NM_003636	0	KCNAB2	potassium voltage-gated channel, shaker-related subfamily, beta member 2
lys10	chr1	6397047	6397287	NM_019089	-5279	HES2	hairy and enhancer of split 2 (Drosophila)
lys11	chr1	8152381	8152723				
lys12	chr1	8180416	8180914				
lys13	chr1	8194485	8195071				
lys14	chr1	9532832	9533193	NM_032315	10719	SLC25A33	solute carrier family 25, member 33
lys15	chr1	9536428	9536814	NM_032315	14315	SLC25A33	solute carrier family 25, member 33
lys16	chr1	10919980	10920450	NM_173507	-26395	C1orf127	chromosome 1 open reading frame 127
lys17	chr1	11910488	11910918	NM_138346	1422	KIAA2013	KIAA2013
lys18	chr1	12507994	12508296	NR_003022	18109	SNORA59B	small nucleolar RNA, H/ACA box 59B
lys19	chr1	12511000	12511457	NM_015378	298302	VPS13D	vacuolar protein sorting 13 homolog D (S. cerevisiae)
lys20	chr1	15608784	15609163	NM_024329	0	EFHD2	EF-hand domain family, member D2
lys21	chr1	15784022	15784349	NM_024758	0	AGMAT	agmatine ureohydrolase (agmatinase)
lys22	chr1	15817903	15818098	NM_032341	1248	DDI2	DNA-damage inducible 1 homolog 2 (S. cerevisiae)
lys23	chr1	17763501	17764065	NM_001011722	-15569	ARHGEF10L	Rho guanine nucleotide exchange factor (GEF) 10-like
lys24	chr1	17791068	17791437	NM_001011722	11435	ARHGEF10L	Rho guanine nucleotide exchange factor (GEF) 10-like
lys25	chr1	19136139	19136426	NM_001136265	-18987	IFFO2	intermediate filament family orphan 2
lys26	chr1	21468804	21469126	NM_001113347	-9644	ECE1	endothelin converting enzyme 1
lys27	chr1	22101900	22102130	NM_005529	-34207	HSPG2	heparan sulfate proteoglycan 2
lys28	chr1	24024452	24024694	NM_000191	0	HMGCL	3-hydroxymethyl-3-methylglutaryl-CoA lyase
lys29	chr1	24318851	24319159	NM_152372	7600	MYOM3	myomesin family, member 3
lys30	chr1	26217782	26218030	NM_004455	-2827	EXTL1	exostoses (multiple)-like 1
lys31	chr1	26699376	26699862				
lys32	chr1	27189641	27190208	NM_001013642	-2573	TRNP1	TMF1-regulated nuclear protein 1
lys33	chr1	27741478	27741863	NM_001029882	-60867	AHDC1	AT hook, DNA binding motif, containing 1
lys34	chr1	28717957	28718184	NM_001048195	627	RCC1	regulator of chromosome condensation 1
lys35	chr1	36702450	36702966	NM_031280	0	MRPS15	mitochondrial ribosomal protein S15
lys36	chr1	38269746	38269995	NM_002699	-15042	POU3F1	POU class 3 homeobox 1
lys37	chr1	39098037	39098291	NM_022157	111	RRAGC	Ras-related GTP binding C
lys38	chr1	40137410	40137590	NM_001033082	-2684	MYCL1	v-myc myelocytomatosis viral oncogene homolog 1, lung carcinoma derived (avian)
lys39	chr1	43277587	43277920				
lys40	chr1	44247880	44248294	NM_006934	-7290	SLC6A9	solute carrier family 6 (neurotransmitter transporter, glycine), member 9
lys41	chr1	44870059	44870388	NM_018150	226514	RNF220	ring finger protein 220
lys42	chr1	44939349	44939796	NM_001145636	26370	C1orf228	chromosome 1 open reading frame 228
lys43	chr1	44946530	44946979	NM_001145636	33551	C1orf228	chromosome 1 open reading frame 228
lys44	chr1	52941520	52941907	NM_023077	4895	C1orf163	chromosome 1 open reading frame 163
lys45	chr1	53359861	53360348	NM_006671	-20529	SLC1A7	solute carrier family 1 (glutamate transporter), member 7
lys46	chr1	53399166	53399598	NM_006671	18290	SLC1A7	solute carrier family 1 (glutamate transporter), member 7
lys47	chr1	53421658	53422100	NM_000098	-12588	CPT2	carnitine palmitoyltransferase 2
lys48	chr1	56651983	56652494				
lys49	chr1	56746641	56747136	NM_003713	-70709	PPAP2B	phosphatidic acid phosphatase type 2B
lys50	chr1	56748329	56748639	NM_003713	-69206	PPAP2B	phosphatidic acid phosphatase type 2B
lys51	chr1	59452952	59453427				
lys52	chr1	65386033	65386259	NM_001005353	0	AK3L1	adenylate kinase 3-like 1
lys53	chr1	67772762	67773296				
lys54	chr1	67790701	67790991				
lys55	chr1	77921083	77921466	NM_015534	153	ZZZ3	zinc finger, ZZ-type containing 3
lys56	chr1	85567314	85567636	NM_012137	-135841	DDAH1	dimethylarginine dimethylaminohydrolase 1
lys57	chr1	87031036	87031725				
lys58	chr1	90232963	90233298	NR_002830	0	LOC492303	-
lys59	chr1	95360483	95360834	NM_152487	5003	TMEM56	transmembrane protein 56
lys60	chr1	109297674	109297893	NM_001048210	-9741	CLCC1	chloride channel CLIC-like 1
lys61	chr1	109618856	109619326	NM_032636	-7968	PSRC1	proline/serine-rich coiled-coil 1
lys62	chr1	109642826	109643109	NM_001010985	-8077	MYBPHL	myosin binding protein H-like
lys63	chr1	111389452	111389768				
lys64	chr1	112099592	112099969	NM_198926	0	C1orf183	chromosome 1 open reading frame 183
lys65	chr1	113561622	113561932				
lys66	chr1	114273050	114273322	NM_198268	-196	HIPK1	homeodomain interacting protein kinase 1
lys67	chr1	116833501	116833851				
lys68	chr1	120068981	120069306	NM_006623	13041	PHGDH	phosphoglycerate dehydrogenase
lys69	chr1	142720950	142721698				
lys70	chr1	144111080	144111283	NM_213653	-13264	HFE2	hemochromatosis type 2 (juvenile)
lys71	chr1	144149531	144149950	NM_006472	0	TXNIP	thioredoxin interacting protein
lys72	chr1	150071741	150072057	NM_005060	770	RORC	RAR-related orphan receptor C
lys73	chr1	152658677	152658999	NM_000565	14386	IL6R	interleukin 6 receptor
lys74	chr1	153239957	153240601	NM_015872	-1134	ZBTB7B	zinc finger and BTB domain containing 7B
lys75	chr1	153242920	153243371	NM_015872	1186	ZBTB7B	zinc finger and BTB domain containing 7B
lys76	chr1	153346480	153346697	NM_004952	28510	EFNA3	ephrin-A3
lys77	chr1	153365004	153365520	NM_182685	-1452	EFNA1	ephrin-A1
lys78	chr1	153537355	153537619	NM_181871	0	PKLR	pyruvate kinase, liver and RBC
lys79	chr1	154452855	154453282	NM_007221	3449	PMF1	polyamine-modulated factor 1
lys80	chr1	158207285	158207836	NM_001146172	16618	SLAMF9	SLAM family member 9
lys81	chr1	159459919	159460381	NM_001643	0	APOA2	apolipoprotein A-II
lys82	chr1	167070362	167070658				
lys83	chr1	169721078	169721342	NM_015172	0	BAT2D1	HLA-B associated transcript 2-like 2
lys84	chr1	177340639	177341094	NM_022371	22906	TOR3A	torsin family 3, member A
lys85	chr1	180524558	180525005				
lys86	chr1	180529085	180529432				
lys87	chr1	181705972	181706220	NM_173156	-2036	SMG7	Smg-7 homolog, nonsense mediated mRNA decay factor (C. elegans)
lys88	chr1	203537109	203537473	NM_030952	-20033	NUAK2	NUAK family, SNF1-like kinase, 2
lys89	chr1	205481555	205481909				
lys90	chr1	209759466	209759988				
lys91	chr1	209819349	209820023	NM_021194	628	SLC30A1	solute carrier family 30 (zinc transporter), member 1

lys92	chr1	209843412	209843680				
lys93	chr1	209883244	209883620	NM_002497	-31970	NEK2	NIMA (never in mitosis gene a)-related kinase 2
lys94	chr1	209907173	209907605	NM_002497	-7985	NEK2	NIMA (never in mitosis gene a)-related kinase 2
lys95	chr1	210848218	210848898	NM_001040619	0	ATF3	activating transcription factor 3
lys96	chr1	211612068	211612550				
lys97	chr1	212196245	212196755				
lys98	chr1	212290405	212290961	NM_002763	61924	PROX1	prospero homeobox 1
lys99	chr1	222019577	222020052	NM_001748	52837	CAPN2	calpain 2, (m/II) large subunit
lys100	chr1	224067749	224068206	NM_001136018	3331	EPHX1	epoxide hydrolase 1, microsomal (xenobiotic)
lys101	chr1	224079408	224079715	NM_000120	0	EPHX1	epoxide hydrolase 1, microsomal (xenobiotic)
lys102	chr1	225194425	225194699	NM_020247	0	CABC1	chaperone, ABC1 activity of bc1 complex homolog (S. pombe)
lys103	chr1	227063439	227063870				
lys104	chr1	228910964	228911494	NM_000029	-5465	AGT	angiotensinogen (serpin peptidase inhibitor, clade A, member 8)
lys105	chr1	228916422	228917086	NM_000029	0	AGT	angiotensinogen (serpin peptidase inhibitor, clade A, member 8)
lys106	chr1	228931223	228931695	NM_000029	14265	AGT	angiotensinogen (serpin peptidase inhibitor, clade A, member 8)
lys107	chr1	232808624	232808932	NM_182972	-2962	IRF2BP2	interferon regulatory factor 2 binding protein 2
lys108	chr1	232823545	232823833	NM_182972	11652	IRF2BP2	interferon regulatory factor 2 binding protein 2
lys109	chr1	233076922	233077214				
lys110	chr1	233159368	233159632				
lys111	chr1	233159744	233160244				
lys112	chr1	233176099	233176416				
lys113	chr1	233177316	233178313				
lys114	chr1	233178610	233178903				
lys115	chr1	233181154	233181488				
lys116	chr1	234269677	234269917	NM_002508	-25187	NID1	nidogen 1
lys117	chr1	234629506	234629870	NM_080738	4169	EDARADD	EDAR-associated death domain
lys118	chr1	234745452	234745686	NM_201545	-2501	LGALS8	lectin, galactoside-binding, soluble, 8
lys119	chr1	236094312	236094517	NR_027247	2216	LOC100130331	-
lys120	chr1	241735845	241736239	NM_006642	249904	SDCCAG8	serologically defined colon cancer antigen 8
lys121	chr10	5748890	5749232	NM_024701	327	ASB13	ankyrin repeat and SOCS box-containing 13
lys122	chr10	5895278	5896190	NM_001494	0	GDI2	GDP dissociation inhibitor 2
lys123	chr10	11983563	11983750	NM_015542	-141064	UPF2	UPF2 regulator of nonsense transcripts homolog (yeast)
lys124	chr10	14614770	14615083	NM_031453	-241819	FAM107B	family with sequence similarity 107, member B
lys125	chr10	22649758	22650132	NM_005180	0	BMI1	BMI1 polycomb ring finger oncogene
lys126	chr10	24870112	24870532	NM_001098501	332388	KIAA1217	KIAA1217
lys127	chr10	30882887	30883342				
lys128	chr10	44811833	44812047	NM_006963	-4231	ZNF22	zinc finger protein 22 (KOX 15)
lys129	chr10	45410174	45410577	NM_145021	0	MARCH8	membrane-associated ring finger (C3HC4) 8
lys130	chr10	54206296	54206626	NM_000242	4831	MBL2	mannose-binding lectin (protein C) 2, soluble (opsonic defect)
lys131	chr10	70855672	70855909	NM_001057	8993	TACR2	tachykinin receptor 2
lys132	chr10	71539166	71539554	NM_032797	-23142	AIFM2	apoptosis-inducing factor, mitochondrion-associated, 2
lys133	chr10	71782170	71782451	NM_207119	-28969	LRRC20	leucine rich repeat containing 20
lys134	chr10	75021694	75021981	NM_152586	16256	USP54	ubiquitin specific peptidase 54
lys135	chr10	77206373	77207159	NM_032024	-5365	C10orf11	chromosome 10 open reading frame 11
lys136	chr10	77454351	77455073	NM_032024	241828	C10orf11	chromosome 10 open reading frame 11
lys137	chr10	79278776	79279387	NM_004747	-76967	DLG5	discs, large homolog 5 (Drosophila)
lys138	chr10	80489016	80489307	NR_024429	-7904	LOC283050	-
lys139	chr10	80614025	80614302	NM_020338	115229	ZMIZ1	zinc finger, MIZ-type containing 1
lys140	chr10	80765202	80765855	NM_005729	-11370	PPIF	peptidylprolyl isomerase F
lys141	chr10	80777613	80777989	NM_005729	389	PPIF	peptidylprolyl isomerase F
lys142	chr10	82155995	82156370	NM_032333	-1851	C10orf58	chromosome 10 open reading frame 58
lys143	chr10	82248548	82248824	NM_001128309	44532	TSPAN14	tetraspanin 14
lys144	chr10	91395472	91395928	NM_148977	278	PANK1	pantothenate kinase 1
lys145	chr10	93382779	93383081	NM_005398	0	PPP1R3C	protein phosphatase 1, regulatory (inhibitor) subunit 3C
lys146	chr10	95982535	95982778	NM_016341	238801	PLCE1	phospholipase C, epsilon 1
lys147	chr10	98427198	98427515	NM_152309	-42754	PIK3AP1	phosphoinositide-3-kinase adaptor protein 1
lys148	chr10	100196596	100196788	NM_000195	0	HPS1	Hermansky-Pudlak syndrome 1
lys149	chr10	102096112	102096496	NM_005063	-265	SCD	stearoyl-CoA desaturase (delta-9-desaturase)
lys150	chr10	102720834	102721212	NM_017893	-1063	SEMA4G	sema domain, immunoglobulin domain (Ig), transmembrane domain (TM) and short cytoplasmic domain, (semaphorin) 4G
lys151	chr10	102862582	102863169	NM_001085398	-17724	TD1	TLX1 neighbor
lys152	chr10	104564683	104565003	NM_000102	-22277	CYP17A1	cytochrome P450, family 17, subfamily A, polypeptide 1
lys153	chr10	105533956	105534171	NM_014631	-70983	SH3PXD2A	SH3 and PX domains 2A
lys154	chr10	114011009	114011585				
lys155	chr10	114015526	114015838	NM_058222	-17644	TECTB	tectorin beta
lys156	chr10	114025622	114026001	NM_058222	-7481	TECTB	tectorin beta
lys157	chr10	114428329	114428702	NM_145206	231585	VTI1A	vesicle transport through interaction with t-SNAREs homolog 1A (yeast)
lys158	chr10	114753978	114754277	NM_030756	53981	TCF7L2	transcription factor 7-like 2 (T-cell specific, HMG-box)
lys159	chr10	114769449	114769885	NM_030756	69452	TCF7L2	transcription factor 7-like 2 (T-cell specific, HMG-box)
lys160	chr10	119141474	119141683	NM_173791	16548	PDZD8	PDZ domain containing 8
lys161	chr10	121020568	121020875	NM_005308	63383	GRK5	G protein-coupled receptor kinase 5
lys162	chr10	121066273	121066524	NM_005308	109088	GRK5	G protein-coupled receptor kinase 5
lys163	chr10	124060248	124060596	NM_144587	39439	BTBD16	BTB (POZ) domain containing 16
lys164	chr10	126403347	126403566	NM_014661	-19354	FAM53B	family with sequence similarity 53, member B
lys165	chr10	128721861	128722412	NM_001380	137850	DOCK1	dedicator of cytokinesis 1
lys166	chr10	135023111	135023896	NM_198472	1593	C10orf125	chromosome 10 open reading frame 125
lys167	chr11	1632421	1632735	NR_026643	-10669	FAM99A	family with sequence similarity 99, member A
lys168	chr11	1667806	1667909	NR_026642	4372	FAM99B	family with sequence similarity 99, member B
lys169	chr11	1668855	1669582	NM_001012416	-5418	KRTAP5-6	keratin associated protein 5-6
lys170	chr11	1930545	1931170	NM_021134	5469	MRPL23	mitochondrial ribosomal protein L23
lys171	chr11	1965433	1965671	NR_024471	-2055	LOC100133545	-
lys172	chr11	1965750	1966887	NR_024471	-839	LOC100133545	-
lys173	chr11	2180242	2180654				
lys174	chr11	2191324	2191679				
lys175	chr11	2710305	2710772	NM_181798	271047	KCNQ1	potassium voltage-gated channel, KQT-like subfamily, member 1
lys176	chr11	3910832	3911202	NM_003156	77325	STIM1	stromal interaction molecule 1
lys177	chr11	6632374	6632852	NM_003737	-798	DCHS1	dachsous 1 (Drosophila)
lys178	chr11	7583369	7583773	NM_003621	91794	PPFIBP2	PTPRF interacting protein, binding protein 2 (liprin beta 2)
lys179	chr11	12045220	12045543				
lys180	chr11	12670837	12671099	NM_021961	18294	TEAD1	TEA domain family member 1 (SV40 transcriptional enhancer factor)
lys181	chr11	12823897	12824440	NM_021961	171354	TEAD1	TEA domain family member 1 (SV40 transcriptional enhancer factor)
lys182	chr11	13118327	13118977				
lys183	chr11	16963518	16963786	NM_175058	-28753	PLEKHA7	pleckstrin homology domain containing, family A member 7
lys184	chr11	17329660	17329962	NR_026750	0	DKFZp686O24166	-
lys185	chr11	17748133	17748619	NM_004976	34064	KCNC1	potassium voltage-gated channel, Shaw-related subfamily, member 1
lys186	chr11	18374053	18374317	NM_001135239	1543	LDHA	lactate dehydrogenase A

lys187	chr11	20014310	20014765	NM_001111019	13100	NAV2	neuron navigator 2
lys188	chr11	44221855	44222139	NM_000401	148182	EXT2	exostosin 2
lys189	chr11	60379971	60380680	NM_004778	0	GPR44	G protein-coupled receptor 44
lys190	chr11	60858404	60858929	NM_001923	1163	DDB1	damage-specific DNA binding protein 1, 127kDa
lys191	chr11	61133393	61133765	NR_002775	-5318	RPLP0P2	ribosomal protein, large, P0 pseudogene 2
lys192	chr11	61497004	61497696	NM_002032	5297	FTH1	ferritin, heavy polypeptide 1
lys193	chr11	62414748	62414914	NM_001013251	9887	SLC3A2	solute carrier family 3 (activators of dibasic and neutral amino acid transport), member 2
lys194	chr11	63443119	63443807	NM_173587	2228	RCOR2	REST corepressor 2
lys195	chr11	63882956	63883329	NM_001006944	0	RPS6KA4	ribosomal protein S6 kinase, 90kDa, polypeptide 4
lys196	chr11	64409564	64409999	NM_006795	6798	EHD1	EH-domain containing 1
lys197	chr11	66967542	66968005	NM_001018070	0	CORO1B	coronin, actin binding protein, 1B
lys198	chr11	67540896	67541156	NM_000694	6532	ALDH3B1	aldehyde dehydrogenase 3 family, member B1
lys199	chr11	67669306	67669787	NM_017635	-67573	SUV420H1	suppressor of variegation 4-20 homolog 1 (Drosophila)
lys200	chr11	67822198	67822542	NM_002335	-14141	LRP5	low density lipoprotein receptor-related protein 5
lys201	chr11	69494023	69494470				
lys202	chr11	71786679	71787545	NM_030813	-35671	CLPB	ClpB caseinolytic peptidase B homolog (E. coli)
lys203	chr11	72166450	72167003	NM_006645	-15395	STARD10	StAR-related lipid transfer (START) domain containing 10
lys204	chr11	73474300	73474626	NM_015531	-85086	C2CD3	C2 calcium-dependent domain containing 3
lys205	chr11	75132536	75132851	NM_025098	25956	MOGAT2	monoacylglycerol O-acyltransferase 2
lys206	chr11	75151544	75151840	NM_032564	-5585	DGAT2	diacylglycerol O-acyltransferase homolog 2 (mouse)
lys207	chr11	75157035	75157238	NM_032564	-187	DGAT2	diacylglycerol O-acyltransferase homolog 2 (mouse)
lys208	chr11	76158247	76158681	NM_015516	-13251	TSKU	tsukushi small leucine rich proteoglycan homolog (Xenopus laevis)
lys209	chr11	77425795	77426067	NM_023930	13828	KCTD14	potassium channel tetramerisation domain containing 14
lys210	chr11	77545094	77545578	NM_024079	16748	ALG8	asparagine-linked glycosylation 8, alpha-1,3-glucosyltransferase homolog (S. cerevisiae)
lys211	chr11	77678986	77679231	NM_012296	-51343	GAB2	GRB2-associated binding protein 2
lys212	chr11	92859930	92860336	NM_020179	-55858	C11orf75	chromosome 11 open reading frame 75
lys213	chr11	95762680	95762962	NM_003772	0	JRKL	jerky homolog-like (mouse)
lys214	chr11	101998246	101998534	NM_004771	-2739	MMP20	matrix metallopeptidase 20
lys215	chr11	113251389	113251905	NM_020886	0	USP28	ubiquitin specific peptidase 28
lys216	chr11	115788235	115788831				
lys217	chr11	116202716	116203189	NM_000040	-2644	APOC3	apolipoprotein C-III
lys218	chr11	116204867	116205381	NM_000040	-452	APOC3	apolipoprotein C-III
lys219	chr11	116209669	116210034	NM_000039	-3514	APOA1	apolipoprotein A-I
lys220	chr11	116213293	116214119	NM_000039	0	APOA1	apolipoprotein A-I
lys221	chr11	116216759	116217161	NM_000039	3212	APOA1	apolipoprotein A-I
lys222	chr11	116397096	116397476	NM_025164	-76727	QSK	SIK family kinase 3
lys223	chr11	117472001	117472319	NM_019894	19066	TMPRSS4	transmembrane protease, serine 4
lys224	chr11	120184455	120184881	NM_014619	148219	GRIK4	glutamate receptor, ionotropic, kainate 4
lys225	chr11	125568188	125568400	NM_001144827	-18397	RPUSD4	RNA pseudouridylate synthase domain containing 4
lys226	chr11	134118085	134118365				
lys227	chr12	189736	190038	NM_003044	-2715	SLC6A12	solute carrier family 6 (neurotransmitter transporter, betaine/GABA), member 12
lys228	chr12	625995	626411	NM_016533	-16605	NINJ2	ninjurin 2
lys229	chr12	1639739	1639991	NM_032642	31068	WNT5B	wingless-type MMTV integration site family, member 5B
lys230	chr12	2227530	2227860	NM_001129835	194855	CACNA1C	calcium channel, voltage-dependent, L type, alpha 1C subunit
lys231	chr12	6513283	6513963	NM_002046	0	GAPDH	glyceraldehyde-3-phosphate dehydrogenase
lys232	chr12	9166012	9166434	NM_000014	6188	A2M	alpha-2-macroglobulin
lys233	chr12	9694674	9694906	NR_002814	2766	LOC374443	-
lys234	chr12	10935845	10936218	NM_023920	-17210	TAS2R13	taste receptor, type 2, member 13
lys235	chr12	20595645	20595773	NM_000921	182183	PDE3A	phosphodiesterase 3A, cGMP-inhibited
lys236	chr12	24798952	24799796				
lys237	chr12	26897847	26898204				
lys238	chr12	46707809	46708063	NM_014554	-77845	SENP1	SUMO1/sentrin specific peptidase 1
lys239	chr12	47490934	47491181	NM_000725	-7597	CACNB3	calcium channel, voltage-dependent, beta 3 subunit
lys240	chr12	48782743	48783010	NM_005276	-1057	GPD1	glycerol-3-phosphate dehydrogenase 1 (soluble)
lys241	chr12	51729653	51730216	NM_170754	0	TENC1	tensin like C1 domain containing phosphatase (tensin 2)
lys242	chr12	51731230	51731545	NM_170754	1130	TENC1	tensin like C1 domain containing phosphatase (tensin 2)
lys243	chr12	51731582	51731896	NM_170754	1482	TENC1	tensin like C1 domain containing phosphatase (tensin 2)
lys244	chr12	52306407	52306841	NM_006856	0	ATF7	activating transcription factor 7
lys245	chr12	54396006	54396228	NM_001487	0	BLOC1S1	biogenesis of lysosomal organelles complex-1, subunit 1
lys246	chr12	54761833	54762347	NM_001005915	1676	ERBB3	v-erb-b2 erythroblastic leukemia viral oncogene homolog 3 (avian)
lys247	chr12	56131386	56131864	NM_031479	-3498	INHBE	inhibin, beta E
lys248	chr12	56134549	56134883	NM_031479	-479	INHBE	inhibin, beta E
lys249	chr12	70120955	70121378	NM_003667	877	LGR5	leucine-rich repeat-containing G protein-coupled receptor 5
lys250	chr12	88275253	88275698	NM_022652	4827	DUSP6	dual specificity phosphatase 6
lys251	chr12	92005240	92005632				
lys252	chr12	92020606	92021011				
lys253	chr12	92191718	92192306				
lys254	chr12	93489476	93489805	NM_020698	-78650	TMCC3	transmembrane and coiled-coil domain family 3
lys255	chr12	96193255	96193502				
lys256	chr12	97616829	97617064	NM_181861	53622	APAF1	apoptotic peptidase activating factor 1
lys257	chr12	99355364	99355637	NM_139319	80378	SLC17A8	solute carrier family 17 (sodium-dependent inorganic phosphate cotransporter), member 8
lys258	chr12	100536126	100536524	NM_206821	23250	MYBPC1	myosin binding protein C, slow type
lys259	chr12	103206652	103207163	NM_182742	1797	TXNRD1	thioredoxin reductase 1
lys260	chr12	105892552	105892748	NM_001033050	-12311	MTERFD3	MTERF domain containing 3
lys261	chr12	106270197	106270532	NM_001018072	33872	BTBD11	BTB (POZ) domain containing 11
lys262	chr12	108511076	108511331	NM_000431	15195	MVK	mevalonate kinase
lys263	chr12	108871241	108871595	NM_014776	-46982	GIT2	G protein-coupled receptor kinase interacting ArfGAP 2
lys264	chr12	115481656	115481982	NM_001085481	89	MAP1LC3B2	microtubule-associated protein 1 light chain 3 beta 2
lys265	chr12	116906235	116906465	NM_173598	15825	KSR2	kinase suppressor of ras 2
lys266	chr12	119188031	119188385	NM_001080855	86	PXN	paxillin
lys267	chr12	119826323	119826683	NM_139015	0	SPPL3	-
lys268	chr12	120008059	120008266				
lys269	chr12	120459195	120459718	NM_001005366	-43029	KDM2B	lysine (K)-specific demethylase 2B
lys270	chr12	121916878	121917070	NM_024667	-29595	VPS37B	vacuolar protein sorting 37 homolog B (S. cerevisiae)
lys271	chr12	122343848	122344390	NM_018183	-56551	SBNO1	strawberry notch homolog 1 (Drosophila)
lys272	chr12	122880132	122880396	NM_207437	67139	DNAH10	dynein, axonemal, heavy chain 10
lys273	chr12	126216822	126216975				
lys274	chr12	129876853	129877115	NM_001980	-12649	STX2	syntaxin 2
lys275	chr12	130911289	130911648	NM_016155	32397	MMP17	matrix metallopeptidase 17 (membrane-inserted)
lys276	chr12	131135410	131135696	NR_003290	631	EP400NL	EP400 N-terminal like

lys277	chr12	131694868	131695220	NM_174873	-10255	P2RX2	purinergic receptor P2X, ligand-gated ion channel, 2
lys278	chr13	19069090	19069312				
lys279	chr13	23042403	23043055	NM_148957	0	TNFRSF19	tumor necrosis factor receptor superfamily, member 19
lys280	chr13	23689799	23690092	NM_153023	56914	SPATA13	spermatogenesis associated 13
lys281	chr13	29396136	29396547				
lys282	chr13	30277297	30277636				
lys283	chr13	33440974	33441493	NM_181558	150770	RFC3	replication factor C (activator 1) 3, 38kDa
lys284	chr13	48775711	48776120	NM_030911	55609	CDADC1	cytidine and dCMP deaminase domain containing 1
lys285	chr13	97758429	97758732	NM_005766	164996	FARP1	FERM, RhoGEF (ARHGEF) and pleckstrin domain protein 1 (chondrocyte-derived)
lys286	chr13	97956989	97957437	NM_003576	-14905	STK24	serine/threonine kinase 24 (STE20 homolog, yeast)
lys287	chr13	112459425	112459840	NM_032189	66783	ATP11A	ATPase, class VI, type 11A
lys288	chr13	112595883	112596278	NM_032189	203241	ATP11A	ATPase, class VI, type 11A
lys289	chr13	112712029	112712369	NM_024979	30375	MCF2L	MCF.2 cell line derived transforming sequence-like
lys290	chr13	112999267	112999605	NM_005561	0	LAMP1	lysosomal-associated membrane protein 1
lys291	chr14	20224442	20224790	NM_001097577	-1981	ANG	angiogenin, ribonuclease, RNase A family, 5
lys292	chr14	43855957	43856199				
lys293	chr14	49178275	49178644	NM_001083908	6578	C14orf104	chromosome 14 open reading frame 104
lys294	chr14	54639650	54640049				
lys295	chr14	61073961	61074536	NM_006255	215695	PRKCH	protein kinase C, eta
lys296	chr14	69226477	69227004	NM_014734	78416	KIAA0247	KIAA0247
lys297	chr14	73308325	73308717	NM_194278	11572	C14orf43	chromosome 14 open reading frame 43
lys298	chr14	87580218	87580693				
lys299	chr14	88576823	88577250				
lys300	chr14	88768446	88768889	NM_005197	-184318	FOXN3	forkhead box N3
lys301	chr14	88887606	88888005	NM_005197	-65202	FOXN3	forkhead box N3
lys302	chr14	90038233	90038569				
lys303	chr14	91493987	91494285	NM_006329	10189	FBLN5	fibulin 5
lys304	chr14	92441950	92442181	NM_001275	-17016	CHGA	chromogranin A (parathyroid secretory protein 1)
lys305	chr14	93438775	93439405	NM_138344	-15605	FAM181A	family with sequence similarity 181, member A
lys306	chr14	93495463	93495779	NM_016150	1944	ASB2	ankyrin repeat and SOCS box-containing 2
lys307	chr14	93813381	93813624	NM_016186	-15490	SERPINA10	serpin peptidase inhibitor, clade A (alpha-1 antiproteinase, antitrypsin), member 10
lys308	chr14	94134906	94136095	NM_001085	-12371	SERPINA3	serpin peptidase inhibitor, clade A (alpha-1 antiproteinase, antitrypsin), member 3
lys309	chr14	95059348	95059870	NR_003002	-9849	SCARNA13	small Cajal body-specific RNA 13
lys310	chr14	95060879	95061250	NR_003002	-8469	SCARNA13	small Cajal body-specific RNA 13
lys311	chr14	98852332	98852959				
lys312	chr14	99088604	99089083	NM_001144995	-51397	CCDC85C	coiled-coil domain containing 85C
lys313	chr14	99102549	99102895	NM_001144995	-37585	CCDC85C	coiled-coil domain containing 85C
lys314	chr14	99138319	99138548	NM_001144995	-1932	CCDC85C	coiled-coil domain containing 85C
lys315	chr14	100353305	100353910	NR_003531	-8287	MEG3	maternally expressed 3 (non-protein coding)
lys316	chr14	100825278	100825683				
lys317	chr14	103417429	103417767				
lys318	chr15	38177941	38178592	NM_001003943	-6987	BMF	Bcl2 modifying factor
lys319	chr15	39081026	39081283	NM_017553	-114349	INO80	INO80 homolog (S. cerevisiae)
lys320	chr15	43469638	43469793	NM_001482	11367	GATM	glycine amidinotransferase (L-arginine:glycine amidinotransferase)
lys321	chr15	43666621	43667038	NM_012388	0	PLDN	pallidin homolog (mouse)
lys322	chr15	48766158	48766500	NM_017672	0	TRPM7	transient receptor potential cation channel, subfamily M, member 7
lys323	chr15	48798198	48798589	NM_032802	-46613	SPPL2A	-
lys324	chr15	51534685	51535305				
lys325	chr15	51910562	51910694				
lys326	chr15	54120001	54120307				
lys327	chr15	56361899	56362224				
lys328	chr15	56548303	56548524	NM_000236	36838	LIPC	lipase, hepatic
lys329	chr15	56626151	56626731	NM_000236	114686	LIPC	lipase, hepatic
lys330	chr15	60891500	60891767	NM_015059	164700	TLN2	talin 2
lys331	chr15	61545633	61546013				
lys332	chr15	63069061	63069405	NM_016630	0	SPG21	spastic paraplegia 21 (autosomal recessive, Mast syndrome)
lys333	chr15	63124143	63124459	NM_178859	-4906	OSTbeta	-
lys334	chr15	65963945	65964465				
lys335	chr15	68158146	68158467	NM_005078	-18843	TLE3	transducin-like enhancer of split 3 (E(sp1) homolog, Drosophila)
lys336	chr15	68696442	68696933				
lys337	chr15	70455314	70455567	NR_027262	0	C15orf34	chromosome 15 open reading frame 34
lys338	chr15	76144696	76145118	NM_015079	-11931	TBC1D2B	TBC1 domain family, member 2B
lys339	chr15	77067945	77068273	NM_153815	-16784	RASGRF1	Ras protein-specific guanine nucleotide-releasing factor 1
lys340	chr15	77073245	77073990	NM_153815	-11067	RASGRF1	Ras protein-specific guanine nucleotide-releasing factor 1
lys341	chr15	78884250	78884936	NM_018689	25485	KIAA1199	KIAA1199
lys342	chr15	82995649	82995913	NM_032856	-2612	WDR73	WD repeat domain 73
lys343	chr15	88034772	88035128	NM_020212	0	WDR93	WD repeat domain 93
lys344	chr15	88347517	88347972	NM_198526	1763	ZNF710	zinc finger protein 710
lys345	chr15	88470971	88471247				
lys346	chr15	89298862	89299276	NM_001017919	0	RCCD1	RCC1 domain containing 1
lys347	chr15	94605881	94606302				
lys348	chr15	96304516	96304998	NM_183376	0	ARRDC4	arrestin domain containing 4
lys349	chr15	98521752	98521989	NM_139057	-177717	ADAMTS17	ADAM metallopeptidase with thrombospondin type 1 motif, 17
lys350	chr15	99609563	99609956	NM_014918	0	CHSY1	chondroitin sulfate synthase 1
lys351	chr15	99797123	99797643	NM_002570	-50067	PCSK6	proprotein convertase subtilisin/kexin type 6
lys352	chr15	99974237	99974591				
lys353	chr16	395725	396072	NM_020664	3868	DECR2	2,4-dienoyl CoA reductase 2, peroxisomal
lys354	chr16	729791	730162	NM_022493	-836	NARFL	nuclear prelamin A recognition factor-like
lys355	chr16	1072214	1072538	NM_001053	3434	SSTR5	somatostatin receptor 5
lys356	chr16	1081248	1081660	NM_207419	-4585	C1QTNF8	C1q and tumor necrosis factor related protein 8
lys357	chr16	1464956	1465261	NM_001114331	0	CLCN7	chloride channel 7
lys358	chr16	1817099	1817319	NM_005326	0	HAGH	hydroxyacylglutathione hydrolase
lys359	chr16	1823074	1823514	NM_031208	5850	FAHD1	fumarylacetoacetate hydrolase domain containing 1
lys360	chr16	2138376	2138617	NM_014353	-34	RAB26	RAB26, member RAS oncogene family
lys361	chr16	2195682	2196057	NM_022372	233	MLST8	MTOR associated protein, LST8 homolog (S. cerevisiae)
lys362	chr16	2205215	2205467	NM_001042371	393	PGP	phosphoglycolate phosphatase
lys363	chr16	2510151	2510534	NM_015944	0	AMDHD2	amidohydrolase domain containing 2
lys364	chr16	4099302	4099711	NM_001116	-6476	ADCY9	adenylate cyclase 9
lys365	chr16	4653681	4654400	NM_015246	38856	MGRN1	mahogunin, ring finger 1
lys366	chr16	8988817	8989094				
lys367	chr16	10649180	10649626	NM_144674	-46677	TEKT5	tektin 5
lys368	chr16	11613739	11614157				
lys369	chr16	19460963	19461341	NM_020314	-13199	C16orf62	chromosome 16 open reading frame 62
lys370	chr16	20517650	20517926				

lys371	chr16	22124772	22125210	NM_013302	0	EEF2K	eukaryotic elongation factor-2 kinase
lys372	chr16	29999741	30000159	NM_002720	4858	PPP4C	protein phosphatase 4, catalytic subunit
lys373	chr16	31024578	31024997	NM_001122957	-2165	BCKDK	branched chain ketoacid dehydrogenase kinase
lys374	chr16	33870004	33870183				
lys375	chr16	33870280	33870497				
lys376	chr16	33870566	33870896				
lys377	chr16	33871402	33871616				
lys378	chr16	33872325	33872493				
lys379	chr16	33873058	33873144				
lys380	chr16	45473666	45474604	NM_133443	-1204	GPT2	glutamic pyruvate transaminase (alanine aminotransferase) 2
lys381	chr16	47033240	47033498				
lys382	chr16	49037133	49037382				
lys383	chr16	50989956	50990372				
lys384	chr16	51647938	51648198	NM_025134	1494	CHD9	chromodomain helicase DNA binding protein 9
lys385	chr16	52357915	52358176	NM_001080432	62541	FTO	fat mass and obesity associated
lys386	chr16	52859848	52860120	NM_024336	-17759	IRX3	iroquois homeobox 3
lys387	chr16	54214505	54214898				
lys388	chr16	57312366	57312902	NM_002080	-12845	GOT2	glutamic-oxaloacetic transaminase 2, mitochondrial (aspartate aminotransferase 2)
lys389	chr16	65756002	65756271	NM_001538	1215	HSF4	heat shock transcription factor 4
lys390	chr16	67383925	67384390	NM_004360	55231	CDH1	cadherin 1, type 1, E-cadherin (epithelial)
lys391	chr16	67384426	67384716	NM_004360	55732	CDH1	cadherin 1, type 1, E-cadherin (epithelial)
lys392	chr16	67995777	67996230	NM_005652	18404	TERF2	telomeric repeat binding factor 2
lys393	chr16	69337461	69337833	NM_018052	-54729	VAC14	Vac14 homolog (S. cerevisiae)
lys394	chr16	69338049	69338421	NM_018052	-54141	VAC14	Vac14 homolog (S. cerevisiae)
lys395	chr16	70608403	70608864	NM_001361	8261	DHODH	dihydroorotate dehydrogenase
lys396	chr16	73582860	73583376	NM_030581	6343	WDR59	WD repeat domain 59
lys397	chr16	73896618	73897128	NM_006324	-127760	CFDP1	craniofacial development protein 1
lys398	chr16	73901249	73901696	NM_006324	-123192	CFDP1	craniofacial development protein 1
lys399	chr16	76888463	76888745	NM_016373	197413	WWOX	WW domain containing oxidoreductase
lys400	chr16	80035704	80036325	NM_198390	0	CMIP	-
lys401	chr16	82534030	82534280	NM_182980	-5892	OSGIN1	oxidative stress induced growth inhibitor 1
lys402	chr16	82544082	82544504	NM_182981	0	OSGIN1	oxidative stress induced growth inhibitor 1
lys403	chr16	83006178	83006444	NM_014861	46546	ATP2C2	ATPase, Ca++ transporting, type 2C, member 2
lys404	chr16	83442376	83442772	NM_031476	31290	CRISPLD2	cysteine-rich secretory protein LCCL domain containing 2
lys405	chr16	83882605	83882955				
lys406	chr16	84144159	84144695				
lys407	chr16	86434036	86434452	NM_003486	-26149	SLC7A5	solute carrier family 7 (cationic amino acid transporter, y+ system), member 5
lys408	chr16	86438545	86439344	NM_003486	-21257	SLC7A5	solute carrier family 7 (cationic amino acid transporter, y+ system), member 5
lys409	chr16	87081840	87082345	NM_153813	34327	ZFPM1	zinc finger protein, multitype 1
lys410	chr16	87810362	87810656	NM_182531	-955	ZNF778	zinc finger protein 778
lys411	chr16	87989106	87989555	NM_013275	-94915	ANKRD11	ankyrin repeat domain 11
lys412	chr16	88281057	88281549	NM_001098533	482	CDK10	cyclin-dependent kinase 10
lys413	chr16	88431527	88431936	NM_032451	9121	SPIRE2	spire homolog 2 (Drosophila)
lys414	chr16	88512384	88512845	NM_002386	598	MC1R	melanocortin 1 receptor (alpha melanocyte stimulating hormone receptor)
lys415	chr17	198434	198677	NM_006987	-3899	RPH3AL	rabphilin 3A-like (without C2 domains)
lys416	chr17	243631	243949	NM_001013672	-16484	C17orf97	chromosome 17 open reading frame 97
lys417	chr17	632159	632505	NM_016080	0	GLOD4	glyoxalase domain containing 4
lys418	chr17	785455	785813	NM_022463	-43947	NXN	nucleoredoxin
lys419	chr17	1574543	1574825	NM_001001870	8289	C17orf91	chromosome 17 open reading frame 91
lys420	chr17	1585926	1586935	NM_000934	-5944	SERPINF2	serpin peptidase inhibitor, clade F (alpha-2 antiplasmin, pigment epithelium derived factor), member 2
lys421	chr17	1586980	1587287	NM_000934	-5592	SERPINF2	serpin peptidase inhibitor, clade F (alpha-2 antiplasmin, pigment epithelium derived factor), member 2
lys422	chr17	1796193	1796483	NM_178568	-78445	RTN4RL1	reticulon 4 receptor-like 1
lys423	chr17	1916625	1917118	NM_001098202	10273	HIC1	hypermethylated in cancer 1
lys424	chr17	3652921	3653392	NM_002208	1636	ITGAE	integrin, alpha E (antigen CD103, human mucosal lymphocyte antigen 1; alpha polypeptide)
lys425	chr17	4331376	4331752	NM_001124758	-17125	SPNS2	spinster homolog 2 (Drosophila)
lys426	chr17	4410861	4411167	NM_153338	237	GGT6	gamma-glutamyltransferase 6
lys427	chr17	7078457	7078801	NM_004422	0	DVL2	dishevelled, dsh homolog 2 (Drosophila)
lys428	chr17	7680796	7681197	NM_001080424	-2762	KDM6B	lysine (K)-specific demethylase 6B
lys429	chr17	14217153	14217700				
lys430	chr17	16269757	16270113	NM_016113	10146	TRPV2	transient receptor potential cation channel, subfamily V, member 2
lys431	chr17	16922291	16922753	NM_015134	35461	MPRIP	myosin phosphatase Rho interacting protein
lys432	chr17	17050275	17050869	NM_178836	0	PLD6	phospholipase D family, member 6
lys433	chr17	17188826	17189182	NM_020201	41423	NT5M	5',3'-nucleotidase, mitochondrial
lys434	chr17	17382737	17383035	NM_148173	-38469	PEMT	phosphatidylethanolamine N-methyltransferase
lys435	chr17	17674684	17675092	NM_001005291	-5958	SREBF1	sterol regulatory element binding transcription factor 1
lys436	chr17	17758077	17758333	NM_001082968	-58176	TOM1L2	target of myb1-like 2 (chicken)
lys437	chr17	19382421	19382729	NM_018242	4664	SLC47A1	solute carrier family 47, member 1
lys438	chr17	19592258	19592739	NM_000691	0	ALDH3A1	aldehyde dehydrogenase 3 family, member A1
lys439	chr17	19821703	19822058	NM_007202	0	AKAP10	A kinase (PRKA) anchor protein 10
lys440	chr17	22683617	22683983	NM_134265	38386	WSB1	WD repeat and SOCS box-containing 1
lys441	chr17	23719389	23719647	NM_001083896	276	SEBOX	SEBOX homeobox
lys442	chr17	24527761	24528295	NM_203318	-3238	MYO18A	myosin XVIIIA
lys443	chr17	28173707	28174003	NM_015194	-54012	MYO1D	myosin ID
lys444	chr17	30502242	30502473	NM_001033576	3295	UNC45B	unc-45 homolog B (C. elegans)
lys445	chr17	33110271	33110539	NM_000458	-68670	HNF1B	HNF1 homeobox B
lys446	chr17	33117520	33117998	NM_000458	-61211	HNF1B	HNF1 homeobox B
lys447	chr17	34113445	34114254	NM_005937	-1144	MLLT6	myeloid/lymphoid or mixed-lineage leukemia (trithorax homolog, Drosophila); translocated to, 6
lys448	chr17	35149262	35149606	NM_001030002	1148	GRB7	growth factor receptor-bound protein 7
lys449	chr17	35482740	35483193	NM_003250	10056	THRA	thyroid hormone receptor, alpha (erythroblastic leukemia viral (v-erb-a) oncogene homolog, avian)
lys450	chr17	35510391	35511317	NM_021724	0	NR1D1	nuclear receptor subfamily 1, group D, member 1
lys451	chr17	35517975	35518299	NM_021724	7477	NR1D1	nuclear receptor subfamily 1, group D, member 1
lys452	chr17	35858431	35858893	NM_001552	5231	IGFBP4	insulin-like growth factor binding protein 4
lys453	chr17	35961569	35962130	NM_001838	-13120	CCR7	chemokine (C-C motif) receptor 7
lys454	chr17	38073700	38073909	NM_024927	-8665	PLEKHH3	pleckstrin homology domain containing, family H (with MyTH4 domain) member 3
lys455	chr17	38083811	38084322	NM_024927	1238	PLEKHH3	pleckstrin homology domain containing, family H (with MyTH4 domain) member 3

lys456	chr17	38283788	38284028	NR_002773	11102	LOC90586	-
lys457	chr17	38291980	38292313	NR_027254	-11964	LOC388387	-
lys458	chr17	38299814	38300185	NR_027254	-4092	LOC388387	-
lys459	chr17	38326398	38326721	NM_000151	20059	G6PC	glucose-6-phosphatase, catalytic subunit
lys460	chr17	38899048	38899240	NM_004941	-17619	DHX8	DEAH (Asp-Glu-Ala-His) box polypeptide 8
lys461	chr17	39211219	39211416	NM_004090	-478	DUSP3	dual specificity phosphatase 3
lys462	chr17	39759748	39759992	NM_001143780	2006	SLC25A39	solute carrier family 25, member 39
lys463	chr17	41369027	41369525	NM_005910	41485	MAPT	microtubule-associated protein tau
lys464	chr17	42204464	42204831	NM_030753	-46250	WNT3	wingless-type MMTV integration site family, member 3
lys465	chr17	42215749	42216081	NM_030753	-35000	WNT3	wingless-type MMTV integration site family, member 3
lys466	chr17	45533822	45534176	NM_002611	6129	PDK2	pyruvate dehydrogenase kinase, isozyme 2
lys467	chr17	45975217	45975712	NM_022827	-3848	SPATA20	spermatogenesis associated 20
lys468	chr17	46068508	46069070	NM_001144070	1293	ABCC3	ATP-binding cassette, sub-family C (CFTR/MRP), member 3
lys469	chr17	46326010	46326296				
lys470	chr17	46341547	46342017				
lys471	chr17	46342320	46342598				
lys472	chr17	46692696	46693035	NM_016001	0	UTP18	UTP18, small subunit (SSU) processome component, homolog (yeast)
lys473	chr17	50718203	50718811	NM_002126	20885	HLF	hepatic leukemia factor
lys474	chr17	50719218	50720050	NM_002126	21900	HLF	hepatic leukemia factor
lys475	chr17	53682052	53682382	NM_001160102	11268	LPO	lactoperoxidase
lys476	chr17	54798978	54799295	NM_001005404	35145	YPEL2	yippee-like 2 (Drosophila)
lys477	chr17	55285309	55285836	NM_016261	-39242	TUBD1	tubulin, delta 1
lys478	chr17	56893058	56893297	NM_018488	4471	TBX4	T-box 4
lys479	chr17	58948565	58948865	NM_030779	-5561	KCNH6	potassium voltage-gated channel, subfamily H (eag-related), member 6
lys480	chr17	59812075	59812374	NM_000442	-5369	PECAM1	platelet/endothelial cell adhesion molecule
lys481	chr17	60468159	60468454	NM_006572	-14928	GNA13	guanine nucleotide binding protein (G protein), alpha 13
lys482	chr17	60988571	60988955	NM_004655	370	AXIN2	axin 2
lys483	chr17	61011738	61012070				
lys484	chr17	63891023	63892111	NM_014960	124107	ARSG	arylsulfatase G
lys485	chr17	63894009	63894519	NM_014960	127093	ARSG	arylsulfatase G
lys486	chr17	64267136	64267562				
lys487	chr17	64301716	64301996				
lys488	chr17	64328386	64329015				
lys489	chr17	64329630	64330023				
lys490	chr17	67907023	67907830				
lys491	chr17	67920862	67921709				
lys492	chr17	68026535	68026903				
lys493	chr17	68139963	68140396	NM_001159770	-460052	SLC39A11	solute carrier family 39 (metal ion transporter), member 11
lys494	chr17	68169077	68169518	NM_001159770	-430930	SLC39A11	solute carrier family 39 (metal ion transporter), member 11
lys495	chr17	68476557	68476836	NM_001159770	-123612	SLC39A11	solute carrier family 39 (metal ion transporter), member 11
lys496	chr17	69938510	69938739	NM_022036	-522	GPRC5C	G protein-coupled receptor, family C, group 5, member C
lys497	chr17	69950318	69950938	NM_018653	8889	GPRC5C	G protein-coupled receptor, family C, group 5, member C
lys498	chr17	71118099	71118435	NR_003587	22367	MYO15B	myosin XVB pseudogene
lys499	chr17	71245903	71246166	NM_001005619	13534	ITGB4	integrin, beta 4
lys500	chr17	71262171	71262586	NM_000154	-10289	GALK1	galactokinase 1
lys501	chr17	72178750	72179204	NM_001008528	-39447	MXRA7	matrix-remodelling associated 7
lys502	chr17	72606695	72606896	NR_003013	9713	SCARNA16	small Cajal body-specific RNA 16
lys503	chr17	72884948	72885415	NM_001113494	1190	SEPT9	septin 9
lys504	chr17	72900045	72900739	NM_001113494	16287	SEPT9	septin 9
lys505	chr17	73653742	73654075	NM_007267	13660	TMC6	transmembrane channel-like 6
lys506	chr17	75348565	75349127	NM_032647	-17460	CBX2	chromobox homolog 2 (Pc class homolog, Drosophila)
lys507	chr17	75578553	75578942	NM_019020	-45300	TBC1D16	TBC1 domain family, member 16
lys508	chr17	75581769	75582416	NM_019020	-41826	TBC1D16	TBC1 domain family, member 16
lys509	chr17	76671592	76672059	NM_017450	48052	BAIAP2	BAI1-associated protein 2
lys510	chr17	76686341	76686699	NM_017450	62801	BAIAP2	BAI1-associated protein 2
lys511	chr17	77289254	77289734	NM_012140	-41	SLC25A10	solute carrier family 25 (mitochondrial carrier; dicarboxylate transporter), member 10
lys512	chr17	77461499	77462078	NM_002861	-508	PCYT2	phosphate cytidylyltransferase 2, ethanolamine
lys513	chr17	77518856	77519484	NM_178493	6510	NOTUM	notum pectinacetylesterase homolog (Drosophila)
lys514	chr17	77542806	77544380	NM_024083	14093	ASPSCR1	alveolar soft part sarcoma chromosome region, candidate 1
lys515	chr17	77545189	77545779	NM_024083	16476	ASPSCR1	alveolar soft part sarcoma chromosome region, candidate 1
lys516	chr17	77551344	77551780	NM_144998	-22282	STRA13	stimulated by retinoic acid 13 homolog (mouse)
lys517	chr17	77551813	77552414	NM_144998	-21648	STRA13	stimulated by retinoic acid 13 homolog (mouse)
lys518	chr17	77654019	77654407	NM_004104	4625	FASN	fatty acid synthase
lys519	chr17	77654792	77655606	NM_004104	5398	FASN	fatty acid synthase
lys520	chr17	77839226	77839556	NM_001893	14365	CSNK1D	casein kinase 1, delta
lys521	chr17	77879672	77880037	NM_003004	-5173	SECTM1	secreted and transmembrane 1
lys522	chr17	78289351	78289663	NM_022158	2612	FN3K	fructosamine 3 kinase
lys523	chr18	693205	693529	NM_202758	-8988	ENOSF1	enolase superfamily member 1
lys524	chr18	838326	838647				
lys525	chr18	9978713	9978988				
lys526	chr18	11998293	11998847	NM_014214	26840	IMPA2	inositol(myo)-1(or 4)-monophosphatase 2
lys527	chr18	13303164	13303370	NM_181481	94380	C18orf1	chromosome 18 open reading frame 1
lys528	chr18	27346140	27346518	NM_001943	14117	DSG2	desmoglein 2
lys529	chr18	27853384	27853645	NM_017831	943	RNF125	ring finger protein 125
lys530	chr18	44561845	44562198	NM_001142397	242422	KIAA0427	KIAA0427
lys531	chr18	44673928	44674314				
lys532	chr18	53062016	53062390				
lys533	chr18	53240540	53240920	NM_004852	-12994	ONECUT2	one cut homeobox 2
lys534	chr18	53590669	53591012				
lys535	chr18	53615404	53615751				
lys536	chr18	54231821	54232745	NM_001144966	192076	NEDD4L	neural precursor cell expressed, developmentally down-regulated 4-like
lys537	chr18	58270536	58271067				
lys538	chr18	58344530	58345236	NM_017742	2894	ZCCHC2	zinc finger, CCHC domain containing 2
lys539	chr18	70067480	70067784	NM_148923	-42417	CYB5A	cytochrome b5 type A (microsomal)
lys540	chr18	70110128	70110842	NM_148923	0	CYB5A	cytochrome b5 type A (microsomal)
lys541	chr18	70510405	70510747	NM_017757	38500	ZNF407	zinc finger protein 407
lys542	chr18	75808876	75809205	NM_001146343	-3436	PQLC1	PQ loop repeat containing 1
lys543	chr19	689544	689954	NM_173481	-12191	C19orf21	chromosome 19 open reading frame 21
lys544	chr19	885895	886199	NM_005224	8860	ARID3A	AT rich interactive domain 3A (BRIGHT-like)
lys545	chr19	903578	904024	NM_005224	26543	ARID3A	AT rich interactive domain 3A (BRIGHT-like)
lys546	chr19	997232	997674	NM_019112	6132	ABCA7	ATP-binding cassette, sub-family A (ABC1), member 7
lys547	chr19	1049337	1049699	NM_002695	2947	POLR2E	polymerase (RNA) II (DNA directed) polypeptide E, 25kDa

lys548	chr19	1324267	1325053	NM_024407	-9829	NDUFS7	NADH dehydrogenase (ubiquinone) Fe-S protein 7, 20kDa (NADH-coenzyme Q reductase)
lys549	chr19	1375353	1375829	NM_001018	-13533	RPS15	ribosomal protein S15
lys550	chr19	3013659	3013881	NM_198969	-83	AES	amino-terminal enhancer of split
lys551	chr19	3017552	3017791	NM_198969	3589	AES	amino-terminal enhancer of split
lys552	chr19	3348315	3348905	NM_005597	30744	NFIC	nuclear factor I/C (CCAAT-binding transcription factor)
lys553	chr19	3364879	3365291	NM_005597	47308	NFIC	nuclear factor I/C (CCAAT-binding transcription factor)
lys554	chr19	4341795	4342392	NM_003025	-9079	SH3GL1	SH3-domain GRB2-like 1
lys555	chr19	4482633	4482889	NM_001013706	-3319	LSDP5	perilipin 5
lys556	chr19	4793627	4793793	NM_182919	10891	TICAM1	toll-like receptor adaptor molecule 1
lys557	chr19	5831305	5832079	NM_002034	9755	FUT5	fucosyltransferase 5 (alpha (1,3) fucosyltransferase)
lys558	chr19	6460374	6460708	NM_006087	7045	TUBB4	tubulin, beta 4
lys559	chr19	7165158	7165724	NM_000208	-79287	INSR	insulin receptor
lys560	chr19	8228953	8229326	NM_024552	48738	LASS4	LAG1 homolog, ceramide synthase 4
lys561	chr19	10062611	10063020	NM_018381	4807	C19orf66	chromosome 19 open reading frame 66
lys562	chr19	10625595	10626091	NM_012218	0	ILF3	interleukin enhancer binding factor 3, 90kDa
lys563	chr19	11210821	11211393	NM_018687	1006	LOC55908	-
lys564	chr19	11237490	11237931	NM_020812	3334	DOCK6	dedicator of cytokinesis 6
lys565	chr19	12303004	12303330	NM_145276	-2204	ZNF563	zinc finger protein 563
lys566	chr19	12694425	12694717	NM_001136196	0	TNPO2	transportin 2
lys567	chr19	13023395	13023752	NM_002501	55813	NFIX	nuclear factor I/X (CCAAT-binding transcription factor)
lys568	chr19	16418826	16419141	NM_021235	-24621	EPS15L1	epidermal growth factor receptor pathway substrate 15-like 1
lys569	chr19	16464816	16465056	NM_145046	-2947	CALR3	calreticulin 3
lys570	chr19	18107838	18108252	NM_005027	-16763	PIK3R2	phosphoinositide-3-kinase, regulatory subunit 2 (beta)
lys571	chr19	18479660	18479997	NM_006532	-13940	ELL	elongation factor RNA polymerase II
lys572	chr19	18811286	18811725	NM_002911	7544	UPF1	UPF1 regulator of nonsense transcripts homolog (yeast)
lys573	chr19	19068813	19069313	NM_178526	33007	SLC25A42	solute carrier family 25, member 42
lys574	chr19	38457101	38457500				
lys575	chr19	38458758	38459297				
lys576	chr19	38473803	38474223	NM_004364	-11047	CEBPA	CCAAT/enhancer binding protein (C/EBP), alpha
lys577	chr19	38575016	38575650	NM_001806	18569	CEBPG	CCAAT/enhancer binding protein (C/EBP), gamma
lys578	chr19	38582831	38583133	NM_001806	26384	CEBPG	CCAAT/enhancer binding protein (C/EBP), gamma
lys579	chr19	38586142	38586730	NM_000285	-117911	PEPD	peptidase D
lys580	chr19	39488951	39489340	NM_014686	51657	KIAA0355	KIAA0355
lys581	chr19	39686451	39686788	NM_001080436	21733	WTIP	Wilms tumor 1 interacting protein
lys582	chr19	42838782	42839026	NM_014898	630	ZFP30	zinc finger protein 30 homolog (mouse)
lys583	chr19	43883792	43884253	NM_004924	53627	ACTN4	actinin, alpha 4
lys584	chr19	43942041	43942363	NM_002307	-13634	LGALS7	lectin, galactoside-binding, soluble, 7
lys585	chr19	45429326	45429647	NM_001144029	4890	CNTD2	cyclin N-terminal domain containing 2
lys586	chr19	46029587	46029846	NM_000762	-18346	CYP2A6	cytochrome P450, family 2, subfamily A, polypeptide 6
lys587	chr19	46423800	46424174	NM_021913	6854	AXL	AXL receptor tyrosine kinase
lys588	chr19	47467049	47467310	NM_015125	-13346	CIC	capicua homolog (Drosophila)
lys589	chr19	49960263	49960476	NM_001130852	-12489	CBLC	Cas-Br-M (murine) ecotropic retroviral transforming sequence c
lys590	chr19	50002245	50002559	NM_005581	-1618	BCAM	basal cell adhesion molecule (Lutheran blood group)
lys591	chr19	50119356	50119711	NM_001645	9597	APOC1	apolipoprotein C-I
lys592	chr19	50120622	50120811	NM_001645	10863	APOC1	apolipoprotein C-I
lys593	chr19	50650819	50651188	NM_001114171	-11904	FOSB	FBJ murine osteosarcoma viral oncogene homolog B
lys594	chr19	53466844	53467177	NM_153608	380	ZNF114	zinc finger protein 114
lys595	chr19	53829441	53829876	NM_001352	-2575	DBP	D site of albumin promoter (albumin D-box) binding protein
lys596	chr19	56551284	56551533	NM_001014763	1377	ETFB	electron-transfer-flavoprotein, beta polypeptide
lys597	chr19	56557746	56558420	NM_001985	-3064	ETFB	electron-transfer-flavoprotein, beta polypeptide
lys598	chr19	61314371	61314617	NM_001002836	-9844	ZNF787	zinc finger protein 787
lys599	chr2	3611042	3611617	NM_024027	-8894	COLEC11	collectin sub-family member 11
lys600	chr2	3630917	3631287	NM_024027	10407	COLEC11	collectin sub-family member 11
lys601	chr2	8667068	8667382				
lys602	chr2	8685306	8685631				
lys603	chr2	10100840	10101140	NM_003597	0	KLF11	Kruppel-like factor 11
lys604	chr2	10421633	10421822	NM_134421	60358	HPCAL1	hippocalcin-like 1
lys605	chr2	10505487	10505722	NM_002539	-182	ODC1	ornithine decarboxylase 1
lys606	chr2	11411971	11412427	NM_004850	9810	ROCK2	Rho-associated, coiled-coil containing protein kinase 2
lys607	chr2	18845074	18845869				
lys608	chr2	20135191	20135591	NM_014713	19922	LAPTM4A	lysosomal protein transmembrane 4 alpha
lys609	chr2	20256012	20256288	NM_002997	-32120	SDC1	syndecan 1
lys610	chr2	20644029	20644387				
lys611	chr2	20656127	20657158				
lys612	chr2	20791384	20791677	NM_021925	-94631	C2orf43	chromosome 2 open reading frame 43
lys613	chr2	21118480	21118938	NM_000384	-1512	APOB	apolipoprotein B (including Ag(x) antigen)
lys614	chr2	21119339	21119867	NM_000384	-583	APOB	apolipoprotein B (including Ag(x) antigen)
lys615	chr2	21123542	21123891	NM_000384	3093	APOB	apolipoprotein B (including Ag(x) antigen)
lys616	chr2	21132649	21133100	NM_000384	12200	APOB	apolipoprotein B (including Ag(x) antigen)
lys617	chr2	26088604	26088843				
lys618	chr2	27286082	27286348	NM_016085	-2054	C2orf28	chromosome 2 open reading frame 28
lys619	chr2	27572729	27573356	NM_001486	0	GCKR	glucokinase (hexokinase 4) regulator
lys620	chr2	36582728	36583114	NM_016441	145829	CRIM1	cysteine rich transmembrane BMP regulator 1 (chordin-like)
lys621	chr2	43007161	43007457				
lys622	chr2	43291012	43291610	NM_006887	-15639	ZFP36L2	zinc finger protein 36, C3H type-like 2
lys623	chr2	43307725	43308113	NR_027251	0	LOC100129726	-
lys624	chr2	46406969	46407237	NM_001430	28904	EPAS1	endothelial PAS domain protein 1
lys625	chr2	46429621	46430050	NM_001430	51556	EPAS1	endothelial PAS domain protein 1
lys626	chr2	47390442	47391225				
lys627	chr2	54655178	54655436	NM_178313	16145	SPTBN1	spectrin, beta, non-erythrocytic 1
lys628	chr2	54710592	54710871	NM_178313	71559	SPTBN1	spectrin, beta, non-erythrocytic 1
lys629	chr2	61617265	61617540	NM_003400	-1382	XPO1	exportin 1 (CRM1 homolog, yeast)
lys630	chr2	62275134	62275614	NM_006577	-1151	B3GNT2	UDP-GlcNAc:betaGal beta-1,3-N-acetylglucosaminyltransferase 2
lys631	chr2	62275948	62276464	NM_006577	-301	B3GNT2	UDP-GlcNAc:betaGal beta-1,3-N-acetylglucosaminyltransferase 2
lys632	chr2	62531294	62531621				
lys633	chr2	63900971	63901466				
lys634	chr2	64734849	64735154	NM_014755	300	SERTAD2	SERTA domain containing 2
lys635	chr2	64811199	64811632				
lys636	chr2	69360572	69361024				
lys637	chr2	69862066	69862408	NM_001153	39437	ANXA4	annexin A4
lys638	chr2	70170275	70170481	NM_006196	2188	PCBP1	poly(rC) binding protein 1

lys639	chr2	70350277	70350592	NM_016297	11544	PCYOX1	prenylcysteine oxidase 1
lys640	chr2	71963574	71964002				
lys641	chr2	73081631	73082118	NM_144579	-70355	SFXN5	sideroflexin 5
lys642	chr2	74228340	74228684	NM_212552	0	BOLA3	bolA homolog 3 (E. coli)
lys643	chr2	74998379	74998650				
lys644	chr2	84374517	84374868	NR_003663	3202	LOC388965	-
lys645	chr2	85387677	85387955	NM_006464	-20930	TGOLN2	trans-golgi network protein 2
lys646	chr2	85501994	85502306	NM_001747	10808	CAPG	capping protein (actin filament), gelsolin-like
lys647	chr2	85665047	85665395	NM_006634	7	VAMP5	vesicle-associated membrane protein 5 (myobrevin)
lys648	chr2	95419984	95420416	NM_016044	-11758	FAHD2A	fumarylacetoacetate hydrolase domain containing 2A
lys649	chr2	99472787	99473446	NM_016316	0	REV1	REV1 homolog (S. cerevisiae)
lys650	chr2	101089472	101089813	NM_001102426	-44465	TBC1D8	TBC1 domain family, member 8 (with GRAM domain)
lys651	chr2	101716104	101716485	NM_145686	35186	MAP4K4	mitogen-activated protein kinase kinase kinase kinase 4
lys652	chr2	101734772	101735038	NM_145686	53854	MAP4K4	mitogen-activated protein kinase kinase kinase kinase 4
lys653	chr2	105784177	105784513	NM_003581	56393	NCK2	NCK adaptor protein 2
lys654	chr2	113119285	113119591	NM_005415	-406	SLC20A1	solute carrier family 20 (phosphate transporter), member 1
lys655	chr2	113601003	113601680	NM_173842	0	IL1RN	interleukin 1 receptor antagonist
lys656	chr2	114451715	114452633	NM_005721	87710	ACTR3	ARP3 actin-related protein 3 homolog (yeast)
lys657	chr2	119899866	119900438	NM_183240	-5477	TMEM37	transmembrane protein 37
lys658	chr2	119900874	119901316	NM_183240	-4599	TMEM37	transmembrane protein 37
lys659	chr2	120940014	120940737	NR_027181	0	LOC84931	-
lys660	chr2	120944297	120944712	NR_027181	3903	LOC84931	-
lys661	chr2	121002310	121002694				
lys662	chr2	127470815	127471091				
lys663	chr2	127471327	127471489				
lys664	chr2	127912171	127912686	NM_000312	19686	PROC	protein C (inactivator of coagulation factors Va and VIIIa)
lys665	chr2	127929021	127929546				
lys666	chr2	128783409	128784052	NM_004807	-8589	HS6ST1	heparan sulfate 6-O-sulfotransferase 1
lys667	chr2	129069181	129069473				
lys668	chr2	130811504	130811820	NM_032357	-4572	CCDC115	coiled-coil domain containing 115
lys669	chr2	132728390	132728501	NR_027020	-3511	NCRNA00164	non-protein coding RNA 164
lys670	chr2	132729291	132729373	NR_027020	-2639	NCRNA00164	non-protein coding RNA 164
lys671	chr2	132731657	132731840	NR_027020	-172	NCRNA00164	non-protein coding RNA 164
lys672	chr2	132754589	132754694				
lys673	chr2	152417510	152418056	NM_001005747	-120675	CACNB4	calcium channel, voltage-dependent, beta 4 subunit
lys674	chr2	152974800	152975166	NM_052905	74805	FMNL2	formin-like 2
lys675	chr2	153050838	153051201	NM_052905	150843	FMNL2	formin-like 2
lys676	chr2	158405430	158405842	NM_001105	-34027	ACVR1	activin A receptor, type I
lys677	chr2	161680365	161680895				
lys678	chr2	161725662	161725968	NM_004180	23952	TANK	TRAF family member-associated NFKB activator
lys679	chr2	162592478	162592755	NM_001935	-46543	DPP4	dipeptidyl-peptidase 4
lys680	chr2	168392234	168392868	NM_020981	8808	B3GALT1	UDP-Gal:betaGlcNAc beta 1,3-galactosyltransferase, polypeptide 1
lys681	chr2	171276572	171277308	NR_027433	-2015	LOC440925	-
lys682	chr2	189623466	189623684	NM_000393	-129166	COL5A2	collagen, type V, alpha 2
lys683	chr2	191382083	191382746				
lys684	chr2	191950276	191950592	NM_001161819	131779	MYO1B	myosin IB
lys685	chr2	192210713	192211085				
lys686	chr2	198186493	198186925	NM_144629	-61904	RFTN2	raftlin family member 2
lys687	chr2	201630609	201631151	NM_173822	-13486	FAM126B	family with sequence similarity 126, member B
lys688	chr2	208420064	208420662	NM_001080475	-177867	PLEKHM3	pleckstrin homology domain containing, family M, member 3
lys689	chr2	211121968	211122366	NM_001875	-7204	CPS1	carbamoyl-phosphate synthase 1, mitochondrial
lys690	chr2	215993486	215993757	NM_054034	-15279	FN1	fibronectin 1
lys691	chr2	216322233	216322638				
lys692	chr2	216860262	216860578	NM_020814	-84417	MARCH4	membrane-associated ring finger (C3HC4) 4
lys693	chr2	219304527	219304921	NM_014640	20717	TTLL4	tubulin tyrosine ligase-like family, member 4
lys694	chr2	219306637	219306941	NM_014640	22827	TTLL4	tubulin tyrosine ligase-like family, member 4
lys695	chr2	219684640	219684951	NM_024782	-48880	NHEJ1	nonhomologous end-joining factor 1
lys696	chr2	220118860	220119256	NM_001005209	1873	TMEM198	transmembrane protein 198
lys697	chr2	224718845	224719143				
lys698	chr2	228386597	228387129	NM_001130046	0	CCL20	chemokine (C-C motif) ligand 20
lys699	chr2	229753731	229753865	NM_017933	-90436	PID1	phosphotyrosine interaction domain containing 1
lys700	chr2	231951274	231951576	NM_145236	-17002	B3GNT7	UDP-GlcNAc:betaGal beta-1,3-N-acetylglucosaminyltransferase 7
lys701	chr2	233123485	233124093	NM_004846	0	EIF4E2	eukaryotic translation initiation factor 4E family member 2
lys702	chr2	233636170	233636803	NM_001017915	2892	INPP5D	inositol polyphosphate-5-phosphatase, 145kDa
lys703	chr2	233961345	233961559	NM_003648	0	DGKD	diacylglycerol kinase, delta 130kDa
lys704	chr2	234862422	234862718				
lys705	chr2	235262398	235262891				
lys706	chr2	235419250	235419693				
lys707	chr2	235571735	235572033	NM_014521	46370	SH3BP4	SH3-domain binding protein 4
lys708	chr2	238307153	238307541	NM_001137552	41609	LRRFIP1	leucine rich repeat (in FLII) interacting protein 1
lys709	chr2	241382386	241382685	NM_004321	-25612	KIF1A	kinesin family member 1A
lys710	chr2	242020849	242021347	NM_014808	76467	FARP2	FERM, RhoGEF and pleckstrin domain protein 2
lys711	chr2	242241491	242241793	NM_178326	15793	ATG4B	ATG4 autophagy related 4 homolog B (S. cerevisiae)
lys712	chr2	242408907	242409263	NM_080741	8163	NEU4	sialidase 4
lys713	chr20	318986	319304	NM_021158	9680	TRIB3	tribbles homolog 3 (Drosophila)
lys714	chr20	1256794	1257101	NM_080489	-737	SDCBP2	syndecan binding protein (syntenin) 2
lys715	chr20	2404673	2404970	NM_003091	5175	SNRPB	small nuclear ribonucleoprotein polypeptides B and B1
lys716	chr20	4890480	4890765	NM_005116	-39380	SLC23A2	solute carrier family 23 (nucleobase transporters), member 2
lys717	chr20	10440115	10440494	NM_001009608	76166	C20orf94	chromosome 20 open reading frame 94
lys718	chr20	10533143	10533561	NM_001009608	169194	C20orf94	chromosome 20 open reading frame 94
lys719	chr20	17609013	17609351	NM_001042576	-1577	RRBP1	ribosome binding protein 1 homolog 180kDa (dog)
lys720	chr20	18066350	18066850	NM_020536	0	CSRP2BP	CSRP2 binding protein
lys721	chr20	19893182	19893557	NM_018993	74974	RIN2	Ras and Rab interactor 2
lys722	chr20	22359390	22359718	NR_027090	10110	LOC284788	-
lys723	chr20	22462695	22463008				
lys724	chr20	23515523	23515684	NM_080610	18138	CST9L	cystatin 9-like
lys725	chr20	23557597	23558066	NM_000099	-8508	CST3	cystatin C
lys726	chr20	23589068	23589406				
lys727	chr20	25467739	25467917	NM_025176	-46236	NINL	ninein-like
lys728	chr20	26136666	26136790				
lys729	chr20	26136832	26136933				
lys730	chr20	26137009	26137105				

lys731	chr20	26137291	26137382				
lys732	chr20	26137787	26138297				
lys733	chr20	29715391	29716209	NM_032609	26041	COX4I2	cytochrome c oxidase subunit IV isoform 2 (lung)
lys734	chr20	29756037	29756386	NM_001191	-17931	BCL2L1	BCL2-like 1
lys735	chr20	30233497	30234149	NR_002781	-7675	TSPYL3	TSPY-like 3 (pseudogene)
lys736	chr20	30591469	30591791				
lys737	chr20	30595262	30595673				
lys738	chr20	31762764	31762950	NM_007238	-8847	PXMP4	peroxisomal membrane protein 4, 24kDa
lys739	chr20	34234107	34234433	NM_012156	28033	EPB41L1	erythrocyte membrane protein band 4.1-like 1
lys740	chr20	35345650	35346063	NM_001003897	-5401	MANBAL	mannosidase, beta A, lysosomal-like
lys741	chr20	35347385	35347870	NM_001003897	-3594	MANBAL	mannosidase, beta A, lysosomal-like
lys742	chr20	35397792	35398207	NM_005417	-8294	SRC	v-src sarcoma (Schmidt-Ruppin A-2) viral oncogene homolog (avian)
lys743	chr20	35405425	35405724	NM_005417	-777	SRC	v-src sarcoma (Schmidt-Ruppin A-2) viral oncogene homolog (avian)
lys744	chr20	35588101	35588426	NM_006698	-1291	BLCAP	bladder cancer associated protein
lys745	chr20	36229920	36230107	NM_004613	2807	TGM2	transglutaminase 2 (C polypeptide, protein-glutamine-gamma-glutamyltransferase)
lys746	chr20	36729559	36729963				
lys747	chr20	39065056	39065584				
lys748	chr20	42456458	42457432	NM_178850	-5905	HNF4A	hepatocyte nuclear factor 4, alpha
lys749	chr20	43028051	43028636	NM_006282	0	STK4	serine/threonine kinase 4
lys750	chr20	43952999	43953526	NM_080749	0	NEURL2	neuralized homolog 2 (Drosophila)
lys751	chr20	43971329	43971609	NM_182676	-2584	PLTP	phospholipid transfer protein
lys752	chr20	44008353	44008779	NM_022104	11631	PCIF1	PDX1 C-terminal inhibiting factor 1
lys753	chr20	44188443	44188876	NM_001250	8132	CD40	CD40 molecule, TNF receptor superfamily member 5
lys754	chr20	44728916	44729533	NM_022829	15412	SLC13A3	solute carrier family 13 (sodium-dependent dicarboxylate transporter), member 3
lys755	chr20	45295636	45295885	NM_012408	-122996	ZMYND8	zinc finger, MYND-type containing 8
lys756	chr20	45773295	45773765	NM_018837	-74450	SULF2	sulfatase 2
lys757	chr20	45787493	45787770	NM_018837	-60445	SULF2	sulfatase 2
lys758	chr20	47633510	47634280	NM_000961	15397	PTGIS	prostaglandin I2 (prostacyclin) synthase
lys759	chr20	48324732	48325289				
lys760	chr20	48355201	48356716				
lys761	chr20	48490417	48490701				
lys762	chr20	49792972	49793347	NM_006045	-24968	ATP9A	ATPase, class II, type 9A
lys763	chr20	49831195	49831509	NM_006045	12881	ATP9A	ATPase, class II, type 9A
lys764	chr20	51836468	51836733				
lys765	chr20	55555696	55556068	NM_002591	-13474	PCK1	phosphoenolpyruvate carboxykinase 1 (soluble)
lys766	chr20	57024347	57025129	NM_030773	-2574	TUBB1	tubulin, beta 1
lys767	chr20	60361478	60361786	NM_005560	-13977	LAMA5	laminin, alpha 5
lys768	chr20	60753859	60754159	NM_016354	9619	SLCO4A1	solute carrier organic anion transporter family, member 4A1
lys769	chr20	61018643	61018953	NM_080796	-9337	DIDO1	death inducer-obliterator 1
lys770	chr20	61679180	61679414	NM_001037335	3145	PRIC285	-
lys771	chr20	61848131	61848566	NM_020062	6478	SLC2A4RG	SLC2A4 regulator
lys772	chr20	62154972	62155205	NM_018419	3550	SOX18	SRY (sex determining region Y)-box 18
lys773	chr21	32625180	32625524	NM_178817	39187	MRAP	melanocortin 2 receptor accessory protein
lys774	chr21	32658510	32658960	NR_002996	-12542	SNORA80	small nucleolar RNA, H/ACA box 80
lys775	chr21	35130247	35130632	NM_001001890	-52225	RUNX1	runt-related transcription factor 1
lys776	chr21	36278843	36279190				
lys777	chr21	37024858	37025372	NM_009586	30999	SIM2	single-minded homolog 2 (Drosophila)
lys778	chr21	44614323	44614551	NM_003307	16413	TRPM2	transient receptor potential cation channel, subfamily M, member 2
lys779	chr21	45671903	45672275	NR_027498	2491	NCRNA00175	non-protein coding RNA 175
lys780	chr21	45693065	45693661	NM_030582	-6190	COL18A1	collagen, type XVIII, alpha 1
lys781	chr22	17911356	17911787	NM_003277	18497	CLDN5	claudin 5
lys782	chr22	18452460	18452857	NM_022720	4628	DGCR8	DiGeorge syndrome critical region gene 8
lys783	chr22	18496421	18496782	NM_013373	-2582	ZDHHC8	zinc finger, DHHC-type containing 8
lys784	chr22	20361980	20362322	NM_148176	11709	PPIL2	peptidylprolyl isomerase (cyclophilin)-like 2
lys785	chr22	22446385	22446776	NM_005940	1351	MMP11	matrix metallopeptidase 11 (stromelysin 3)
lys786	chr22	23231356	23231650	NM_016327	10107	UPB1	ureidopropionase, beta
lys787	chr22	23232450	23232954	NM_016327	11201	UPB1	ureidopropionase, beta
lys788	chr22	23240197	23240598	NM_016327	18948	UPB1	ureidopropionase, beta
lys789	chr22	23275940	23276861	NM_031444	-4414	C22orf13	chromosome 22 open reading frame 13
lys790	chr22	23620479	23620757	NM_001098497	88345	SGSM1	small G protein signaling modulator 1
lys791	chr22	27528194	27528502	NM_001079539	1635	XBP1	X-box binding protein 1
lys792	chr22	27649803	27650258	NM_032173	39915	ZNRF3	zinc and ring finger 3
lys793	chr22	28934900	28935246				
lys794	chr22	29231129	29231492	NM_001161368	-206	SEC14L4	SEC14-like 4 (S. cerevisiae)
lys795	chr22	30975303	30975558	NM_014227	-5760	SLC5A4	solute carrier family 5 (low affinity glucose cotransporter), member 4
lys796	chr22	31506371	31506717	NM_133633	-226089	SYN3	synapsin III
lys797	chr22	31996749	31996977	NM_133642	-649439	LARGE	like-glycosyltransferase
lys798	chr22	35145241	35145739				
lys799	chr22	36211057	36211341	NM_002405	-990	MFNG	MFNG O-fucosylpeptide 3-beta-N-acetylglucosaminyltransferase
lys800	chr22	36227655	36228171	NM_002405	15325	MFNG	MFNG O-fucosylpeptide 3-beta-N-acetylglucosaminyltransferase
lys801	chr22	36903847	36904271	NM_003560	-3436	PLA2G6	phospholipase A2, group VI (cytosolic, calcium-independent)
lys802	chr22	37009719	37009956	NM_012264	10758	TMEM184B	transmembrane protein 184B
lys803	chr22	39949417	39949786	NM_138481	-17095	CHADL	chondroadherin-like
lys804	chr22	40034540	40034844	NM_017590	7029	ZC3H7B	zinc finger CCCH-type containing 7B
lys805	chr22	40140450	40140728	NM_016272	-32245	TOB2	transducer of ERBB2, 2
lys806	chr22	41639803	41640110	NM_007229	-32881	PACSIN2	protein kinase C and casein kinase substrate in neurons 2
lys807	chr22	42104278	42104672				
lys808	chr22	44047084	44047357	NM_006953	-12195	UPK3A	uroplakin 3A
lys809	chr22	44055229	44055542	NM_006953	-4010	UPK3A	uroplakin 3A
lys810	chr22	44634814	44635054	NM_013236	188465	ATXN10	ataxin 10
lys811	chr22	45227694	45228355	NM_014246	-83376	CELSR1	cadherin, EGF LAG seven-pass G-type receptor 1 (flamingo homolog, Drosophila)
lys812	chr22	45506117	45506486	NM_022766	-6330	CERK	ceramide kinase
lys813	chr22	45573201	45573580	NM_014346	35990	TBC1D22A	TBC1 domain family, member 22A
lys814	chr22	48732135	48732384	NM_001001852	-7762	PIM3	pim-3 oncogene
lys815	chr22	48919603	48919887	NM_018995	48984	MOV10L1	Mov10l1, Moloney leukemia virus 10-like 1, homolog (mouse)
lys816	chr22	49270673	49271004	NM_017584	-1074	MIOX	myo-inositol oxygenase
lys817	chr22	49354035	49354414	NM_001123225	5842	C22orf41	chromosome 22 open reading frame 41
lys818	chr22	49426966	49427607	NM_001085426	13494	ARSA	arylsulfatase A
lys819	chr3	4994607	4996291	NM_003670	0	BHLHE40	basic helix-loop-helix family, member e40

lys820	chr3	5003437	5003934	NM_003670	7342	BHLHE40	basic helix-loop-helix family, member e40
lys821	chr3	14694511	14694968	NM_032137	2855	C3orf20	chromosome 3 open reading frame 20
lys822	chr3	15222387	15222808	NR_027927	0	DVWA	collagen, type VI, alpha 4 pseudogene 1
lys823	chr3	28131477	28131846				
lys824	chr3	31985504	31985860	NM_017784	-12382	OSBPL10	oxysterol binding protein-like 10
lys825	chr3	33197870	33198076	NM_015551	-37635	SUSD5	sushi domain containing 5
lys826	chr3	42096265	42096519	NM_001042646	-11230	TRAK1	trafficking protein, kinesin binding 1
lys827	chr3	50183967	50184508	NM_004186	16117	SEMA3F	sema domain, immunoglobulin domain (Ig), short basic domain, secreted, (semaphorin) 3F
lys828	chr3	50221170	50221759	NM_006841	3476	SLC38A3	solute carrier family 38, member 3
lys829	chr3	50600606	50601186	NM_016173	18695	HEMK1	HemK methyltransferase family member 1
lys830	chr3	51629641	51629901	NM_015106	79007	RAD54L2	RAD54-like 2 (S. cerevisiae)
lys831	chr3	52464359	52464680	NM_007184	0	NISCH	nischarin
lys832	chr3	52713597	52714185	NM_014041	-711	SPCS1	signal peptidase complex subunit 1 homolog (S. cerevisiae)
lys833	chr3	58394562	58394989	NM_000925	0	PDHB	pyruvate dehydrogenase (lipoamide) beta
lys834	chr3	67429207	67429528				
lys835	chr3	81848805	81849232	NM_000158	-44408	GBE1	glucan (1,4-alpha-), branching enzyme 1
lys836	chr3	81893455	81893762	NM_000158	0	GBE1	glucan (1,4-alpha-), branching enzyme 1
lys837	chr3	121027406	121027678	NM_022002	43161	NR1I2	nuclear receptor subfamily 1, group I, member 2
lys838	chr3	121476292	121476653				
lys839	chr3	123995218	123995495	NM_024610	0	HSPBAP1	HSPB (heat shock 27kDa) associated protein 1
lys840	chr3	124095769	124096120	NR_024618	7721	LOC100129550	-
lys841	chr3	126036238	126036821	NM_002213	-52013	ITGB5	integrin, beta 5
lys842	chr3	128294927	128295262				
lys843	chr3	129851640	129851977	NM_002950	-432	RPN1	ribophorin I
lys844	chr3	132704281	132704613	NM_007208	0	MRPL3	mitochondrial ribosomal protein L3
lys845	chr3	142143060	142143416	NM_018155	0	SLC25A36	solute carrier family 25, member 36
lys846	chr3	142349259	142349489	NM_080862	95828	SPSB4	splA/ryanodine receptor domain and SOCS box containing 4
lys847	chr3	142569585	142570072	NM_001080412	43842	ZBTB38	zinc finger and BTB domain containing 38
lys848	chr3	158320923	158321396				
lys849	chr3	158335163	158335440	NM_020307	-25736	CCNL1	cyclin L1
lys850	chr3	158337147	158337724	NM_020307	-23452	CCNL1	cyclin L1
lys851	chr3	159568849	159569123	NM_016625	258265	RSRC1	arginine/serine-rich coiled-coil 1
lys852	chr3	173330374	173330719	NM_001135095	89338	FNDC3B	fibronectin type III domain containing 3B
lys853	chr3	178312035	178312372	NM_024665	-85370	TBL1XR1	transducin (beta)-like 1 X-linked receptor 1
lys854	chr3	178398279	178398644	NM_024665	538	TBL1XR1	transducin (beta)-like 1 X-linked receptor 1
lys855	chr3	178539142	178539664				
lys856	chr3	178559428	178559730				
lys857	chr3	179024629	179024874				
lys858	chr3	185514858	185515113	NM_198242	0	EIF4G1	eukaryotic translation initiation factor 4 gamma, 1
lys859	chr3	188061372	188061602	NM_004797	18217	ADIPOQ	adiponectin, C1Q and collagen domain containing
lys860	chr3	189198371	189198791				
lys861	chr3	189946578	189947158	NM_005578	533165	LPP	LIM domain containing preferred translocation partner in lipoma
lys862	chr3	194912577	194913090	NM_015560	118952	OPA1	optic atrophy 1 (autosomal dominant)
lys863	chr3	194944456	194944860				
lys864	chr3	195696988	195697373				
lys865	chr3	196307432	196307693	NM_152531	-165491	C3orf21	chromosome 3 open reading frame 21
lys866	chr3	197100826	197101229	NM_001010938	-5600	TNK2	tyrosine kinase, non-receptor, 2
lys867	chr3	198581980	198582416				
lys868	chr3	198676420	198676787				
lys869	chr4	677046	677373	NM_032219	4074	MFSD7	major facilitator superfamily domain containing 7
lys870	chr4	1748097	1748458	NM_000142	-16378	FGFR3	fibroblast growth factor receptor 3
lys871	chr4	1769392	1770048	NM_000142	4557	FGFR3	fibroblast growth factor receptor 3
lys872	chr4	3314063	3314258	NM_198229	28393	RGS12	regulator of G-protein signaling 12
lys873	chr4	3802560	3803051				
lys874	chr4	3808344	3808834				
lys875	chr4	4594952	4595179	NM_016930	277	STX18	syntaxin 18
lys876	chr4	6946236	6946581	NM_001113361	-15490	TBC1D14	TBC1 domain family, member 14
lys877	chr4	24923197	24923521	NM_024936	0	ZCCHC4	zinc finger, CCHC domain containing 4
lys878	chr4	37326068	37326465	NM_001085399	-37929	RELL1	RELT-like 1
lys879	chr4	38169110	38169471				
lys880	chr4	38341769	38342281	NM_016531	0	KLF3	Kruppel-like factor 3 (basic)
lys881	chr4	77446519	77446815	NM_003943	0	STBD1	starch binding domain 1
lys882	chr4	84150925	84151470	NM_001115008	0	LIN54	lin-54 homolog (C. elegans)
lys883	chr4	88418505	88419011				
lys884	chr4	104216003	104216301	NM_178833	-1318	NHEDC2	Na+/H+ exchanger domain containing 2
lys885	chr4	124619386	124619916				
lys886	chr4	159135493	159135804				
lys887	chr4	166252849	166253147	NM_001100389	-327	TMEM192	transmembrane protein 192
lys888	chr5	1231982	1232218				
lys889	chr5	1608379	1608919	NR_003263	-38727	SDHAP3	succinate dehydrogenase complex, subunit A, flavoprotein pseudogene 3
lys890	chr5	5414185	5414518				
lys891	chr5	30540810	30541163				
lys892	chr5	38185065	38185291				
lys893	chr5	71182498	71182587				
lys894	chr5	71182613	71182694				
lys895	chr5	72876015	72876762	NM_023039	-20492	ANKRA2	ankyrin repeat, family A (RFXANK-like), 2
lys896	chr5	74207602	74208429	NM_015566	9232	FAM169A	family with sequence similarity 169, member A
lys897	chr5	75735770	75736372	NM_006633	867	IQGAP2	IQ motif containing GTPase activating protein 2
lys898	chr5	90242666	90243061	NM_032119	352295	GPR98	G protein-coupled receptor 98
lys899	chr5	90246975	90247320	NM_032119	356604	GPR98	G protein-coupled receptor 98
lys900	chr5	95659416	95659752				
lys901	chr5	132022273	132022562	NM_002188	511	IL13	interleukin 13
lys902	chr5	132202127	132202500	NM_133456	12227	SHROOM1	shroom family member 1
lys903	chr5	133869722	133870355	NM_015288	-19341	PHF15	PHD finger protein 15
lys904	chr5	134497563	134497969				
lys905	chr5	134626718	134627278				
lys906	chr5	134693655	134694137	NM_001040158	-68690	H2AFY	H2A histone family, member Y
lys907	chr5	138561812	138562253	NM_001037633	0	SIL1	SIL1 homolog, endoplasmic reticulum chaperone (S. cerevisiae)
lys908	chr5	139002794	139003275	NM_016463	-5209	CXXC5	CXXC finger 5
lys909	chr5	140978672	140979165	NM_005219	0	DIAPH1	diaphanous homolog 1 (Drosophila)
lys910	chr5	141372639	141372974	NM_005471	0	GNPDA1	glucosamine-6-phosphate deaminase 1

lys911	chr5	143054162	143054489				
lys912	chr5	149882142	149882765	NM_001543	14277	NDST1	N-deacetylase/N-sulfotransferase (heparan glucosaminyl) 1
lys913	chr5	156650885	156651318	NM_014376	21947	CYFIP2	cytoplasmic FMR1 interacting protein 2
lys914	chr5	170748040	170748318	NM_001037738	639	NPM1	nucleophosmin (nucleolar phosphoprotein B23, numatrin)
lys915	chr5	172192303	172192613	NM_001031711	-1215	ERGIC1	endoplasmic reticulum-golgi intermediate compartment (ERGIC) 1
lys916	chr5	172212262	172212835	NM_001031711	18435	ERGIC1	endoplasmic reticulum-golgi intermediate compartment (ERGIC) 1
lys917	chr5	172218281	172218654	NM_001031711	24454	ERGIC1	endoplasmic reticulum-golgi intermediate compartment (ERGIC) 1
lys918	chr5	172218808	172219365	NM_001031711	24981	ERGIC1	endoplasmic reticulum-golgi intermediate compartment (ERGIC) 1
lys919	chr5	172973110	172973391	NM_001159651	-2881	BOD1	biorientation of chromosomes in cell division 1
lys920	chr5	172982967	172983227	NM_001159651	6696	BOD1	biorientation of chromosomes in cell division 1
lys921	chr5	173030728	173031073				
lys922	chr5	173162313	173162667				
lys923	chr5	173166294	173166583				
lys924	chr5	173328957	173329608	NM_001144954	-19159	C5orf47	chromosome 5 open reading frame 47
lys925	chr5	178624574	178624824	NM_014244	-80111	ADAMTS2	ADAM metallopeptidase with thrombospondin type 1 motif, 2
lys926	chr6	1209272	1209541				
lys927	chr6	2460163	2460549				
lys928	chr6	3084520	3084758	NM_001069	-18024	TUBB2A	tubulin, beta 2A
lys929	chr6	3858817	3859103				
lys930	chr6	5004648	5004932				
lys931	chr6	5151152	5151460	NM_020408	-54707	LYRM4	LYR motif containing 4
lys932	chr6	7164608	7164901	NM_001003699	111423	RREB1	ras responsive element binding protein 1
lys933	chr6	7488006	7488577	NM_001008844	1139	DSP	desmoplakin
lys934	chr6	7506515	7507166	NM_001008844	19648	DSP	desmoplakin
lys935	chr6	11467249	11467690	NM_001142393	-22877	NEDD9	neural precursor cell expressed, developmentally down-regulated 9
lys936	chr6	11500747	11501158	NM_001142393	10181	NEDD9	neural precursor cell expressed, developmentally down-regulated 9
lys937	chr6	11501265	11501603	NM_001142393	10699	NEDD9	neural precursor cell expressed, developmentally down-regulated 9
lys938	chr6	31977291	31977620	NM_181842	-128	ZBTB12	zinc finger and BTB domain containing 12
lys939	chr6	32003039	32003329	NM_000063	0	C2	complement component 2
lys940	chr6	33689018	33689402	NM_002224	-7736	ITPR3	inositol 1,4,5-triphosphate receptor, type 3
lys941	chr6	37203778	37204094				
lys942	chr6	37223788	37224166				
lys943	chr6	41595028	41595395				
lys944	chr6	41595511	41595879				
lys945	chr6	42227084	42227361	NM_000409	-3760	GUCA1A	guanylate cyclase activator 1A (retina)
lys946	chr6	43343396	43343809	NM_032538	24198	TTBK1	tau tubulin kinase 1
lys947	chr6	43345703	43346497	NM_032538	26505	TTBK1	tau tubulin kinase 1
lys948	chr6	44299336	44299786	NM_001078177	0	SLC29A1	solute carrier family 29 (nucleoside transporters), member 1
lys949	chr6	47148820	47149416				
lys950	chr6	47151514	47152171				
lys951	chr6	47152511	47152968				
lys952	chr6	47438656	47438917				
lys953	chr6	53107546	53108146	NM_003643	-13437	GCM1	glial cells missing homolog 1 (Drosophila)
lys954	chr6	86410168	86410460	NM_001159677	407	SYNCRIP	synaptotagmin binding, cytoplasmic RNA interacting protein
lys955	chr6	87921562	87922094	NM_015021	0	ZNF292	zinc finger protein 292
lys956	chr6	96359572	96360138				
lys957	chr6	96370046	96370353				
lys958	chr6	109810240	109810578	NM_006016	0	CD164	CD164 molecule, sialomucin
lys959	chr6	110982848	110983375				
lys960	chr6	111686983	111687389	NM_153369	0	KIAA1919	KIAA1919
lys961	chr6	126184615	126185030	NM_181782	30923	NCOA7	nuclear receptor coactivator 7
lys962	chr6	126440133	126440424				
lys963	chr6	129713203	129713525	NM_000426	467226	LAMA2	laminin, alpha 2
lys964	chr6	132339364	132339874				
lys965	chr6	132343496	132344141				
lys966	chr6	138086075	138086574				
lys967	chr6	138086868	138087234				
lys968	chr6	138585866	138586076	NM_021635	4547	PBOV1	prostate and breast cancer overexpressed 1
lys969	chr6	149395807	149396149	NM_005715	285845	UST	uronyl-2-sulfotransferase
lys970	chr6	151228978	151229465	NM_015440	596	MTHFD1L	methylenetetrahydrofolate dehydrogenase (NADP+ dependent) 1-like
lys971	chr6	155677839	155678050	NM_016020	522	TFB1M	transcription factor B1, mitochondrial
lys972	chr6	158573070	158573389				
lys973	chr6	158663743	158664259	NM_020245	10065	TULP4	tubby like protein 4
lys974	chr6	160103679	160103983	NM_005891	702	ACAT2	acetyl-CoA acetyltransferase 2
lys975	chr6	160478190	160478893	NM_153187	15339	SLC22A1	solute carrier family 22 (organic cation transporter), member 1
lys976	chr6	166694588	166694927	NM_145169	18608	SFT2D1	SFT2 domain containing 1
lys977	chr6	167731728	167732249	NM_004610	13741	TCP10	t-complex 10 homolog (mouse)
lys978	chr6	167811760	167812109				
lys979	chr6	167819570	167820038				
lys980	chr6	167826475	167827179				
lys981	chr6	167905808	167906215				
lys982	chr6	169941512	169942006	NR_026781	667	C6orf122	non-protein coding RNA 242
lys983	chr7	558898	559343	NM_002735	-159316	PRKAR1B	protein kinase, cAMP-dependent, regulatory, type I, beta
lys984	chr7	963997	964472	NM_006869	3183	ADAP1	ArfGAP with dual PH domains 1
lys985	chr7	966624	966910	NM_006869	5810	ADAP1	ArfGAP with dual PH domains 1
lys986	chr7	1719948	1720388				
lys987	chr7	1824067	1824146	NM_001013837	-414963	MAD1L1	MAD1 mitotic arrest deficient-like 1 (yeast)
lys988	chr7	2320462	2320950	NM_013321	0	SNX8	sorting nexin 8
lys989	chr7	2669294	2669557	NM_133463	-16131	AMZ1	archaelysin family metallopeptidase 1
lys990	chr7	3004835	3005047	NM_032415	-45058	CARD11	caspase recruitment domain family, member 11
lys991	chr7	4140933	4141208	NM_001079653	5092	SDK1	sidekick homolog 1, cell adhesion molecule (chicken)
lys992	chr7	4647739	4648437				
lys993	chr7	4748321	4748605	NM_001037165	59867	FOXK1	forkhead box K1
lys994	chr7	5540102	5540329	NM_001101	3345	ACTB	actin, beta
lys995	chr7	12646512	12646802	NM_033128	50841	SCIN	scinderin
lys996	chr7	12653918	12654216	NM_033128	58247	SCIN	scinderin
lys997	chr7	12947731	12948021				
lys998	chr7	17105681	17106068				
lys999	chr7	22860237	22860746	NR_003075	-2010	SNORD93	small nucleolar RNA, C/D box 93
lys1000	chr7	23340848	23341143	NM_006547	-135377	IGF2BP3	insulin-like growth factor 2 mRNA binding protein 3
lys1001	chr7	27699978	27700228				
lys1002	chr7	29412030	29412368	NM_004067	211386	CHN2	chimerin (chimaerin) 2

lys1003	chr7	36298169	36298481	NM_030636	138810	EEPD1	endonuclease/exonuclease/phosphatase family domain containing 1
lys1004	chr7	40761530	40761893	NM_024728	620432	C7orf10	chromosome 7 open reading frame 10
lys1005	chr7	41322554	41322853				
lys1006	chr7	47506310	47506676	NM_022748	-39048	TNS3	tensin 3
lys1007	chr7	47721907	47722234				
lys1008	chr7	51300850	51301092	NM_015198	-50917	COBL	cordon-bleu homolog (mouse)
lys1009	chr7	65245491	65245674	NM_001040647	28253	CRCP	CGRP receptor component
lys1010	chr7	72700114	72700649	NM_001077621	-19460	VPS37D	vacuolar protein sorting 37 homolog D (S. cerevisiae)
lys1011	chr7	73546555	73546864	NM_005685	40184	GTF2IRD1	GTF2I repeat domain containing 1
lys1012	chr7	75155789	75156312	NM_005338	-49903	HIP1	huntingtin interacting protein 1
lys1013	chr7	75320076	75320433				
lys1014	chr7	75387717	75388419	NM_000941	5363	POR	P450 (cytochrome) oxidoreductase
lys1015	chr7	75716635	75716994	NM_001110199	47485	FLJ37078	serine/arginine repetitive matrix 3
lys1016	chr7	87015872	87016159	NM_000927	-164341	ABCB1	ATP-binding cassette, sub-family B (MDR/TAP), member 1
lys1017	chr7	95789191	95789751	NM_014251	0	SLC25A13	solute carrier family 25, member 13 (citrin)
lys1018	chr7	95794743	95794960	NM_014251	5349	SLC25A13	solute carrier family 25, member 13 (citrin)
lys1019	chr7	99459652	99459947	NM_003439	8499	ZKSCAN1	zinc finger with KRAB and SCAN domains 1
lys1020	chr7	99472293	99472815	NM_145914	-12537	ZSCAN21	zinc finger and SCAN domain containing 21
lys1021	chr7	99879929	99880579	NM_145030	7900	C7orf47	chromosome 7 open reading frame 47
lys1022	chr7	99981254	99981636	NM_006076	6486	AGFG2	ArfGAP with FG repeats 2
lys1023	chr7	100003810	100004046	NM_002319	-17666	LRCH4	leucine-rich repeats and calponin homology (CH) domain containing 4
lys1024	chr7	100129935	100130263	NM_022574	5130	GIGYF1	GRB10 interacting GYF protein 1
lys1025	chr7	100556264	100556722	NM_000602	-376	SERPINE1	serpin peptidase inhibitor, clade E (nexin, plasminogen activator inhibitor type 1), member 1
lys1026	chr7	100556747	100557145	NM_000602	0	SERPINE1	serpin peptidase inhibitor, clade E (nexin, plasminogen activator inhibitor type 1), member 1
lys1027	chr7	101703671	101704284	NM_020979	-10840	SH2B2	SH2B adaptor protein 2
lys1028	chr7	130248464	130249118				
lys1029	chr7	130392405	130392812	NR_024153	-49517	FLJ43663	-
lys1030	chr7	137452211	137452455	NM_005989	40477	AKR1D1	aldo-keto reductase family 1, member D1 (delta 4-3-ketosteroid-5-beta-reductase)
lys1031	chr7	139575777	139576148				
lys1032	chr7	139576827	139577071				
lys1033	chr7	149696450	149697243	NM_013400	0	REPIN1	replication initiator 1
lys1034	chr7	150390306	150390728	NM_003040	2718	SLC4A2	solute carrier family 4, anion exchanger, member 2 (erythrocyte membrane protein band 3-like 1)
lys1035	chr7	155168004	155168330	NM_053043	38042	RBM33	RNA binding motif protein 33
lys1036	chr7	156759008	156759305	NM_014671	134594	UBE3C	ubiquitin protein ligase E3C
lys1037	chr7	157339693	157340112	NM_130843	-733131	PTPRN2	protein tyrosine phosphatase, receptor type, N polypeptide 2
lys1038	chr7	157749714	157750107	NM_130843	-323136	PTPRN2	protein tyrosine phosphatase, receptor type, N polypeptide 2
lys1039	chr7	157984219	157984543	NM_130843	-88700	PTPRN2	protein tyrosine phosphatase, receptor type, N polypeptide 2
lys1040	chr7	158067183	158067475	NM_130843	-5768	PTPRN2	protein tyrosine phosphatase, receptor type, N polypeptide 2
lys1041	chr7	158076122	158076567	NM_130843	2880	PTPRN2	protein tyrosine phosphatase, receptor type, N polypeptide 2
lys1042	chr7	158111882	158112076	NM_017760	-78205	NCAPG2	non-SMC condensin II complex, subunit G2
lys1043	chr8	570938	571215				
lys1044	chr8	8322641	8322914				
lys1045	chr8	8324292	8324650				
lys1046	chr8	9677377	9677690	NM_003747	226524	TNKS	tankyrase, TRF1-interacting ankyrin-related ADP-ribose polymerase
lys1047	chr8	10465210	10465483				
lys1048	chr8	11772051	11772280	NM_147781	8997	CTSB	cathepsin B
lys1049	chr8	17239827	17240140	NM_004686	-75271	MTMR7	myotubularin related protein 7
lys1050	chr8	17516557	17516881	NM_006207	37573	PDGFRL	platelet-derived growth factor receptor-like
lys1051	chr8	17690270	17690554	NM_001001925	-12152	MTUS1	microtubule associated tumor suppressor 1
lys1052	chr8	17797687	17798346	NM_201552	361	FGL1	fibrinogen-like 1
lys1053	chr8	17809078	17809394	NM_201552	11752	FGL1	fibrinogen-like 1
lys1054	chr8	21937461	21937749	NM_182795	-550	NPM2	nucleophosmin/nucleoplasmin 2
lys1055	chr8	22270055	22270370	NM_001135154	-10336	SLC39A14	solute carrier family 39 (zinc transporter), member 14
lys1056	chr8	22324902	22325252	NM_001135153	43909	SLC39A14	solute carrier family 39 (zinc transporter), member 14
lys1057	chr8	25113102	25113376	NM_024940	14900	DOCK5	dedicator of cytokinesis 5
lys1058	chr8	27213129	27213643	NM_171982	-11108	TRIM35	tripartite motif-containing 35
lys1059	chr8	28315273	28315778	NM_018660	15378	ZNF395	zinc finger protein 395
lys1060	chr8	29686887	29687200				
lys1061	chr8	33081969	33082399				
lys1062	chr8	37651913	37652351				
lys1063	chr8	37876115	37876579	NM_001002814	0	RAB11FIP1	RAB11 family interacting protein 1 (class I)
lys1064	chr8	42517364	42517872	NM_006749	1140	SLC20A2	solute carrier family 20 (phosphate transporter), member 2
lys1065	chr8	49117492	49117722	NM_003350	33946	UBE2V2	ubiquitin-conjugating enzyme E2 variant 2
lys1066	chr8	53770403	53770828	NM_014781	-18751	RB1CC1	RB1-inducible coiled-coil 1
lys1067	chr8	59247701	59248035				
lys1068	chr8	71477246	71477682	NM_006540	-892	NCOA2	nuclear receptor coactivator 2
lys1069	chr8	92189312	92189616	NM_001129890	5291	LRRC69	leucine rich repeat containing 69
lys1070	chr8	96182758	96183184				
lys1071	chr8	98244781	98245138	NM_016134	518108	PGCP	-
lys1072	chr8	103497952	103498230	NM_015902	4282	UBR5	ubiquitin protein ligase E3 component n-recognin 5
lys1073	chr8	103735201	103735815	NM_001032282	0	KLF10	Kruppel-like factor 10
lys1074	chr8	103737053	103737899	NM_005655	0	KLF10	Kruppel-like factor 10
lys1075	chr8	103887548	103888671	NM_015878	-56902	AZIN1	antizyme inhibitor 1
lys1076	chr8	103888785	103889220	NM_015878	-56353	AZIN1	antizyme inhibitor 1
lys1077	chr8	119062190	119062415	NM_000127	-130824	EXT1	exostosin 1
lys1078	chr8	121890950	121891481	NM_021021	-2009	SNTB1	syntrophin, beta 1 (dystrophin-associated protein A1, 59kDa, basic component 1)
lys1079	chr8	121909043	121909397	NM_021021	15554	SNTB1	syntrophin, beta 1 (dystrophin-associated protein A1, 59kDa, basic component 1)
lys1080	chr8	122430174	122430538				
lys1081	chr8	125712499	125712933	NM_014751	-96978	MTSS1	metastasis suppressor 1
lys1082	chr8	125805667	125806195	NM_014751	-3716	MTSS1	metastasis suppressor 1
lys1083	chr8	126493879	126494304	NM_025195	-17440	TRIB1	tribbles homolog 1 (Drosophila)
lys1084	chr8	126510575	126510829	NM_025195	-915	TRIB1	tribbles homolog 1 (Drosophila)
lys1085	chr8	126583016	126583561				
lys1086	chr8	129636257	129636942				
lys1087	chr8	129637924	129638462				
lys1088	chr8	134451329	134451654				

lys1089	chr8	134530106	134530448	NM_173344	-122917	ST3GAL1	ST3 beta-galactoside alpha-2,3-sialyltransferase 1
lys1090	chr8	141050068	141050376	NM_001160372	-486667	TRAPPC9	trafficking protein particle complex 9
lys1091	chr8	142307730	142308067	NM_001080431	0	SLC45A4	solute carrier family 45, member 4
lys1092	chr8	144672564	144673045	NM_015117	-21718	ZC3H3	zinc finger CCCH-type containing 3
lys1093	chr8	145048723	145049202	NM_201384	-36544	PLEC1	plectin
lys1094	chr9	6670936	6671471				
lys1095	chr9	6887871	6888129	NM_015061	140232	KDM4C	lysine (K)-specific demethylase 4C
lys1096	chr9	19151753	19152008				
lys1097	chr9	37371391	37371714				
lys1098	chr9	37956688	37956948	NM_003028	-102262	SHB	Src homology 2 domain containing adaptor protein B
lys1099	chr9	38371008	38371443	NM_000692	-11258	ALDH1B1	aldehyde dehydrogenase 1 family, member B1
lys1100	chr9	38378834	38379176	NM_000692	-3525	ALDH1B1	aldehyde dehydrogenase 1 family, member B1
lys1101	chr9	70621898	70622137	NM_003558	111464	PIP5K1B	phosphatidylinositol-4-phosphate 5-kinase, type I, beta
lys1102	chr9	72275206	72275492				
lys1103	chr9	78376476	78376706				
lys1104	chr9	92840102	92840386				
lys1105	chr9	92999254	92999680	NM_001698	-164347	AUH	AU RNA binding protein/enoyl-CoA hydratase
lys1106	chr9	92999896	93000295	NM_001698	-163732	AUH	AU RNA binding protein/enoyl-CoA hydratase
lys1107	chr9	94914523	94914881	NM_032310	16254	C9orf89	chromosome 9 open reading frame 89
lys1108	chr9	94935701	94935960	NM_004148	-431	NINJ1	ninjurin 1
lys1109	chr9	95003672	95004197	NM_006648	16641	WNK2	WNK lysine deficient protein kinase 2
lys1110	chr9	96773554	96773856	NM_032823	244741	C9orf3	chromosome 9 open reading frame 3
lys1111	chr9	96792008	96792398	NM_032823	263195	C9orf3	chromosome 9 open reading frame 3
lys1112	chr9	96833838	96834324	NM_032823	305025	C9orf3	chromosome 9 open reading frame 3
lys1113	chr9	97573658	97573941				
lys1114	chr9	98238541	98238869	NM_014282	-13365	HABP4	hyaluronan binding protein 4
lys1115	chr9	98414288	98414568	NM_033331	-7365	CDC14B	CDC14 cell division cycle 14 homolog B (S. cerevisiae)
lys1116	chr9	103125966	103126316	NM_017753	138830	PRG-3	-
lys1117	chr9	106667000	106667274	NM_005502	-62983	ABCA1	ATP-binding cassette, sub-family A (ABC1), member 1
lys1118	chr9	110696369	110696709	NM_003640	-39720	IKBKAP	inhibitor of kappa light polypeptide gene enhancer in B-cells, kinase complex-associated protein
lys1119	chr9	115925798	115926169				
lys1120	chr9	115965660	115965958	NM_032888	7610	COL27A1	collagen, type XXVII, alpha 1
lys1121	chr9	122373902	122374342	NM_018249	-7916	CDK5RAP2	CDK5 regulatory subunit associated protein 2
lys1122	chr9	125272319	125272737	NM_024820	-459501	DENND1A	DENN/MADD domain containing 1A
lys1123	chr9	128329448	128329956				
lys1124	chr9	128507452	128507708	NM_002316	90885	LMX1B	LIM homeobox transcription factor 1, beta
lys1125	chr9	129409613	129409850	NM_001032221	-4456	STXBP1	syntaxin binding protein 1
lys1126	chr9	129824217	129824603				
lys1127	chr9	129905079	129905489	NM_001006643	4424	SLC25A25	solute carrier family 25 (mitochondrial carrier; phosphate carrier), member 25
lys1128	chr9	129917320	129917716	NM_025072	-12579	PTGES2	prostaglandin E synthase 2
lys1129	chr9	131145864	131146262				
lys1130	chr9	131245136	131245374				
lys1131	chr9	131359528	131359930				
lys1132	chr9	131360853	131361500				
lys1133	chr9	132315670	132315976	NM_054012	5757	ASS1	argininosuccinate synthase 1
lys1134	chr9	132959923	132960185	NM_031426	-1547	AIF1L	allograft inflammatory factor 1-like
lys1135	chr9	133471406	133471787	NM_198679	-103263	RAPGEF1	Rap guanine nucleotide exchange factor (GEF) 1
lys1136	chr9	135913202	135913610	NM_007371	-9352	BRD3	bromodomain containing 3
lys1137	chr9	135996240	135996544	NM_052821	1507	WDR5	WD repeat domain 5
lys1138	chr9	136355381	136355665	NM_002957	-2471	RXRA	retinoid X receptor, alpha
lys1139	chr9	136370162	136370456	NM_002957	12027	RXRA	retinoid X receptor, alpha
lys1140	chr9	136401300	136401709	NM_002957	43165	RXRA	retinoid X receptor, alpha
lys1141	chr9	138755294	138755534	NM_001001712	-1698	LCN10	lipocalin 10
lys1142	chr9	139249937	139250233	NM_080877	4733	SLC34A3	solute carrier family 34 (sodium phosphate), member 3
lys1143	chr9	139469162	139469514	NM_015537	-4093	NELF	nasal embryonic LHRH factor
lys1144	chr9	139685157	139685459	NM_024757	51894	EHMT1	euchromatic histone-lysine N-methyltransferase 1
lys1145	chrX	20234768	20234968				
lys1146	chrX	38547747	38548363	NM_021242	0	MID1IP1	MID1 interacting protein 1 (gastrulation specific G12 homolog (zebrafish))
lys1147	chrX	87746509	87746882				
lys1148	chrX	108184307	108184493				
lys1149	chrX	108869926	108870261	NM_004458	6650	ACSL4	acyl-CoA synthetase long-chain family member 4
lys1150	chrX	117994037	117994427	NM_024778	1298	LONRF3	LON peptidase N-terminal domain and ring finger 3
lys1151	chrX	152541735	152541934	NM_001395	-19156	DUSP9	dual specificity phosphatase 9
lys1152	chrX	153850856	153851026	NM_000132	-53166	F8	coagulation factor VIII, procoagulant component
lys1153	chrX	153866131	153866276	NM_000132	-37916	F8	coagulation factor VIII, procoagulant component
