# Supplementary material for: Integrated Expression Profiling and Genome-Wide Analysis of ChREBP Targets Reveals the Dual Role for ChREBP in Glucose-Regulated Gene Expression
Source: PLoS One. 2011 Jul 21;6(7):e22544. doi: 10.1371/journal.pone.0022544 (PMC3141076; doi:10.1371/journal.pone.0022544)
Supplement: Table S2 — Functional annotation clustering of the ChREBP ChIP-seq dataset. To reduce redundancy, the newly developed functional annotation clustering report groups and displays similar annotations together to make the biology clearer and more focused for reading when compared to a traditional chart report. For annotation clustering, we used GOTERM_BP_ALL, GOTERM_MF_ALL, PANTHER_BP_ALL, and PANTHER_MF_ALL. (PDF) [file pone.0022544.s004.pdf]

Tablse S2. Functional annotation clustering of the ChREBP ChIP-seq dataset.

**Annotation Cluster 1**	**Enrichment Score: 2.62**				
Category	Term	Number	%	*p* -Value	FDR
GOTERM_MF_ALL	GO:0046983~protein dimerization activity	41	5.5	2.4E-04	0.4
GOTERM_MF_ALL	GO:0042803~protein homodimerization activity	26	3.5	2.8E-03	4.3
GOTERM_MF_ALL	GO:0046982~protein heterodimerization activity	18	2.4	5.4E-03	8.1

**Annotation Cluster 2**	**Enrichment Score: 2.54**				
Category	Term	Number	%	*p* -Value	FDR
GOTERM_BP_ALL	GO:0006066~alcohol metabolic process	39	5.2	2.5E-05	0.0
GOTERM_BP_ALL	GO:0044262~cellular carbohydrate metabolic process	34	4.5	1.8E-04	0.3
GOTERM_BP_ALL	GO:0005975~carbohydrate metabolic process	41	5.5	4.8E-04	0.9
GOTERM_BP_ALL	GO:0005996~monosaccharide metabolic process	22	2.9	7.0E-04	1.2
GOTERM_BP_ALL	GO:0006006~glucose metabolic process	16	2.1	2.7E-03	4.8
GOTERM_BP_ALL	GO:0044275~cellular carbohydrate catabolic process	11	1.5	3.7E-03	6.5
GOTERM_BP_ALL	GO:0019318~hexose metabolic process	18	2.4	4.3E-03	7.5

**Annotation Cluster 3**	**Enrichment Score: 2.52**				
Category	Term	Number	%	*p* -Value	FDR
GOTERM_BP_ALL	GO:0009790~embryonic development	42	5.6	1.0E-03	1.8
GOTERM_BP_ALL	GO:0043009~chordate embryonic development	27	3.6	2.7E-03	4.7
GOTERM_BP_ALL	GO:0009792~embryonic development ending in birth or egg hatching	27	3.6	3.0E-03	5.3

**Annotation Cluster 4**	**Enrichment Score: 2.16**				
Category	Term	Number	%	*p* -Value	FDR
PANTHER_BP_ALL	BP00019:Lipid, fatty acid and steroid metabolism	51	6.8	2.3E-04	0.3
GOTERM_BP_ALL	GO:0046486~glycerolipid metabolic process	18	2.4	6.9E-04	1.2

**Annotation Cluster 5**	**Enrichment Score: 2.07**				
Category	Term	Number	%	*p* -Value	FDR
GOTERM_MF_ALL	GO:0030234~enzyme regulator activity	58	7.8	1.2E-04	0.2
PANTHER_MF_ALL	MF00100:G-protein modulator	30	4.0	5.5E-04	0.7
GOTERM_MF_ALL	GO:0030695~GTPase regulator activity	30	4.0	2.5E-03	3.8
GOTERM_MF_ALL	GO:0008047~enzyme activator activity	26	3.5	2.9E-03	4.5
GOTERM_MF_ALL	GO:0060589~nucleoside-triphosphatase regulator activity	30	4.0	3.5E-03	5.3
GOTERM_MF_ALL	GO:0005083~small GTPase regulator activity	22	2.9	4.5E-03	6.8
GOTERM_BP_ALL	GO:0046578~regulation of Ras protein signal transduction	19	2.5	4.7E-03	8.2

**Annotation Cluster 6**	**Enrichment Score: 1.96**				
Category	Term	Number	%	*p* -Value	FDR
GOTERM_BP_ALL	GO:0006639~acylglycerol metabolic process	13	1.7	8.7E-07	0.0
GOTERM_BP_ALL	GO:0006638~neutral lipid metabolic process	13	1.7	1.1E-06	0.0
GOTERM_BP_ALL	GO:0006662~glycerol ether metabolic process	13	1.7	1.4E-06	0.0
GOTERM_BP_ALL	GO:0018904~organic ether metabolic process	13	1.7	2.1E-06	0.0
GOTERM_BP_ALL	GO:0033700~phospholipid efflux	6	0.8	3.2E-05	0.1
GOTERM_BP_ALL	GO:0034381~lipoprotein particle clearance	7	0.9	3.6E-05	0.1
GOTERM_BP_ALL	GO:0006641~triglyceride metabolic process	10	1.3	7.9E-05	0.1
GOTERM_BP_ALL	GO:0065005~protein-lipid complex assembly	6	0.8	9.4E-05	0.2
GOTERM_BP_ALL	GO:0034377~plasma lipoprotein particle assembly	6	0.8	9.4E-05	0.2
GOTERM_BP_ALL	GO:0044269~glycerol ether catabolic process	5	0.7	3.7E-04	0.7
GOTERM_BP_ALL	GO:0046464~acylglycerol catabolic process	5	0.7	3.7E-04	0.7
GOTERM_BP_ALL	GO:0046461~neutral lipid catabolic process	5	0.7	3.7E-04	0.7
GOTERM_BP_ALL	GO:0015914~phospholipid transport	8	1.1	4.5E-04	0.8
GOTERM_BP_ALL	GO:0046339~diacylglycerol metabolic process	5	0.7	6.0E-04	1.1
GOTERM_BP_ALL	GO:0046486~glycerolipid metabolic process	18	2.4	6.9E-04	1.2
GOTERM_BP_ALL	GO:0046503~glycerolipid catabolic process	5	0.7	1.3E-03	2.3
GOTERM_BP_ALL	GO:0046463~acylglycerol biosynthetic process	5	0.7	1.3E-03	2.3
GOTERM_BP_ALL	GO:0046460~neutral lipid biosynthetic process	5	0.7	1.3E-03	2.3
GOTERM_BP_ALL	GO:0034367~macromolecular complex remodeling	6	0.8	1.4E-03	2.4
GOTERM_BP_ALL	GO:0034368~protein-lipid complex remodeling	6	0.8	1.4E-03	2.4
GOTERM_BP_ALL	GO:0034369~plasma lipoprotein particle remodeling	6	0.8	1.4E-03	2.4

GOTERM_BP_ALL	GO:0046504~glycerol ether biosynthetic process	5	0.7	1.9E-03	3.3
GOTERM_BP_ALL	GO:0010873~positive regulation of cholesterol esterification	4	0.5	2.5E-03	4.4
GOTERM_MF_ALL	GO:0005543~phospholipid binding	17	2.3	2.6E-03	3.9
GOTERM_BP_ALL	GO:0019433~triglyceride catabolic process	4	0.5	3.9E-03	6.7
GOTERM_BP_ALL	GO:0010872~regulation of cholesterol esterification	4	0.5	3.9E-03	6.7
GOTERM_BP_ALL	GO:0043691~reverse cholesterol transport	5	0.7	4.2E-03	7.3
GOTERM_BP_ALL	GO:0006869~lipid transport	15	2.0	4.3E-03	7.4
GOTERM_BP_ALL	GO:0030301~cholesterol transport	7	0.9	4.9E-03	8.3
GOTERM_BP_ALL	GO:0015918~sterol transport	7	0.9	4.9E-03	8.3
GOTERM_MF_ALL	GO:0070653~high-density lipoprotein receptor binding	3	0.4	4.9E-03	7.4
GOTERM_BP_ALL	GO:0010903~negative regulation of very-low-density lipoprotein particle remodeling	3	0.4	5.5E-03	9.4
GOTERM_BP_ALL	GO:0042632~cholesterol homeostasis	7	0.9	5.6E-03	9.5
GOTERM_BP_ALL	GO:0055092~sterol homeostasis	7	0.9	5.6E-03	9.5
GOTERM_BP_ALL	GO:0034374~low-density lipoprotein particle remodeling	4	0.5	5.7E-03	9.6

**Annotation Cluster 7**	**Enrichment Score: 1.77**				
Category	Term	Number	%	*p*-Value	FDR
GOTERM_BP_ALL	GO:0032502~developmental process	167	22.3	2.7E-03	4.7
GOTERM_BP_ALL	GO:0009653~anatomical structure morphogenesis	73	9.8	2.9E-03	5.0
GOTERM_BP_ALL	GO:0007275~multicellular organismal development	152	20.3	4.6E-03	8.0
GOTERM_BP_ALL	GO:0048856~anatomical structure development	136	18.2	4.7E-03	8.1

**Annotation Cluster 8**	**Enrichment Score: 1.70**				
Category	Term	Number	%	*p*-Value	FDR
GOTERM_MF_ALL	GO:0008092~cytoskeletal protein binding	36	4.8	1.7E-03	2.6

**Annotation Cluster 9**	**Enrichment Score: 1.61**				
Category	Term	Number	%	*p*-Value	FDR
GOTERM_BP_ALL	GO:0005996~monosaccharide metabolic process	22	2.9	7.0E-04	1.2
GOTERM_BP_ALL	GO:0044275~cellular carbohydrate catabolic process	11	1.5	3.7E-03	6.5
GOTERM_BP_ALL	GO:0019318~hexose metabolic process	18	2.4	4.3E-03	7.5

**Annotation Cluster 10**	**Enrichment Score: 1.57**				
Category	Term	Number	%	*p*-Value	FDR
GOTERM_MF_ALL	GO:0043169~cation binding	203	27.1	3.2E-03	4.9
GOTERM_MF_ALL	GO:0043167~ion binding	205	27.4	3.9E-03	5.9
GOTERM_MF_ALL	GO:0046872~metal ion binding	199	26.6	6.0E-03	8.9

**Annotation Cluster 11**	**Enrichment Score: 1.56**				
Category	Term	Number	%	*p*-Value	FDR
GOTERM_BP_ALL	GO:0016043~cellular component organization	138	18.4	1.7E-03	3.0

**Annotation Cluster 12**	**Enrichment Score: 1.56**				
Category	Term	Number	%	*p*-Value	FDR
GOTERM_MF_ALL	GO:0015291~secondary active transmembrane transporter activity	19	2.5	2.2E-03	3.4
GOTERM_MF_ALL	GO:0015171~amino acid transmembrane transporter activity	9	1.2	2.7E-03	4.1
GOTERM_MF_ALL	GO:0015179~L-amino acid transmembrane transporter activity	7	0.9	4.2E-03	6.3
GOTERM_MF_ALL	GO:0022804~active transmembrane transporter activity	27	3.6	4.2E-03	6.4
GOTERM_BP_ALL	GO:0046942~carboxylic acid transport	15	2.0	4.9E-03	8.3
GOTERM_BP_ALL	GO:0015849~organic acid transport	15	2.0	5.2E-03	8.8

**Annotation Cluster 13**	**Enrichment Score: 1.44**				
Category	Term	Number	%	*p*-Value	FDR
GOTERM_BP_ALL	GO:0030201~heparan sulfate proteoglycan metabolic process	6	0.8	2.2E-04	0.4
GOTERM_BP_ALL	GO:0015012~heparan sulfate proteoglycan biosynthetic process	5	0.7	9.2E-04	1.6

**Annotation Cluster 14**	**Enrichment Score: 1.40**				
Category	Term	Number	%	*p*-Value	FDR
GOTERM_BP_ALL	GO:0034637~cellular carbohydrate biosynthetic process	10	1.3	2.9E-03	5.1
