# Supplementary material for: Integrated Expression Profiling and Genome-Wide Analysis of ChREBP Targets Reveals the Dual Role for ChREBP in Glucose-Regulated Gene Expression
Source: PLoS One. 2011 Jul 21;6(7):e22544. doi: 10.1371/journal.pone.0022544 (PMC3141076; doi:10.1371/journal.pone.0022544)
Supplement: Table S3 — Primer sequences for the measurement of human DNA or RNA using an ABI Prism 7900HT system. All primers are listed in the 5′ to 3′ orientation. (PDF) [file pone.0022544.s005.pdf]

Table S3. Primer sequences for qPCR.

qPCR primers for ChIP-seq validation		
**Symbol**	**Forward primers**	**Reverse primers**
**Cyclo**	TGCCATCGCCAAGGAGTAG	TGCACAGACGGTCACTCAAA
**PKLR**	GTGGGTTTTGGAGAGGAACG	GGCCAGAGTCCAGGAACCA
**PK-up**	ATCTACCCAACTGCTCAAGCCA	TGCATTTTGGAGAAGAGCCAG
**DGKD**	TCTTGACACCCCCACCCA	TTGACCTATGCACAGGCCC
**DNAH10**	TGTGTGTTTACAACACTCAGTGATTCA	AAGGCAGCTAAGCATGACGC
**EHD1**	AGTGCATGGGCTTTGGAGC	CCTAGGAGGCGGGTGCTT
**FTO**	GGGTGAATCAGGTGCTATCCC	CCTTCGGATTCCCTCTCCAC
**ITGAE**	CACAAACCCAAACTACATCCCC	TGTGTATTTGTCAAACAGCCAGC
**LIPC-1**	TGGACACTCTGGCTGGTCAA	GGGCAGTGGAGCTTAGGTACAT
**LIPC-2**	CTCCGCATCATCTCATTTTGG	TGGGAGGGATGTAAGCAATGATA
**RAD54L2**	TTTATAGTGGTGGTGTTCATAATTCATTC	ACGTGAGCCAGCTGTGAGC
**RASGRF1-1**	AATCATGTGCTCCCACCACC	TCGTGACAGCCCTGAGAACA
**RASGRF1-2**	CAGCTGCCGAGAGGAGTACTTC	AGGGAGGTTGACGGTCCC
**SCD1**	TCCAAAATTACAACCTGCCAGTC	GTTCAGGACCCGGGCG
**TANK**	AACTTCCCAGAGGGTCACGG	GTGAGGACTCTACCTAACGCCTG
**BCKDK**	CCAGATTCTGGCGCACAGA	TGGTAAAAGGGAGCGGGAA
**BHLHE40**	GAGTCAGACGCGGGCG	AGAAGTTGGTAACGTGGGCG
**COL5A2**	ATATTGACCGATGCAGCTACTCAC	CGACCGGGCAGAGAGGA
**DGAT2-1**	TGTGTAATATCCCCTCCCCCA	TTGCATCATTCTGCCTGCTT
**DGAT2-2**	AAGCGGGACCTTCGCATC	GTGCGGTGCAGGCAGAG
**ECE1**	GAACTCTGCGGTCTGGCCT	GAACTCTGCGGTCTGGCCT
**ELOVL6**	CCGCCTTGCTTTGAATGAAG	GCAGGTTTTACTATTTCCCAATCGT
**TRIB3**	GCAGAGCCCATGGCTGTC	GTCCTCTTTGCCCACCCAC
**TXNIP**	CCAGAGCGCAACAACCATTT	TGCCCGGTCCTTGTTTACC
**UPB1-1**	CTGCCCTTGTGCCAAGAAG	AGCAGGGCAGATGAAAGCAA
**UPB1-2**	TGCTTCTTAAATAGACCTTTGACCAG	TTATCCAAAGCTCAGGACCCAG
**UPB1-3**	ACGTGTCTGTGTCCCAGAAGTTC	CACAGATACTATCCTCATGGAGCTCA
**MTUS1-1**	GCCCAGGATTGGCGCTAT	TGGAAACTTTGTCCGTTGAATG
**MTUS1-2**	AATCACGTGTGGGAGGTTAAGTC	AAGCTTATTGTAGTGTCTTTTTTCAAACAT
**PDK2**	CCAGCAGCTGTGGATACGAG	GGGAGTGTGTGTAATTGTGTGCA
**G6PC**	ACTCTTGACTCCGGCCCTCT	CTGGCTGCACACTTTATCCTGT
**GPD1**	GTCTGCCAAGCCAGGAGGT	TGATCTGGAGAGTGGAGCAAGAG
**IL6R**	GCCAAGCCTGTGTCCTCG	TGCCGCAGCTCTTGCC
**INHBE-1**	CCCCTGACAGTCCTGATGGA	CGTGTTCCTGTGATGTCTGGG
**INHBE-2**	GCATCATGCGTGTCCACATT	TCCTCCTCCCCATCAGCTC
**FASN-1**	ACAAGGGCAAACTCTATCTCAAAAA	CTCACCCCAGCCACAGTGT
**FASN-2**	CAGGCGTGACTCCCTGTGA	GAGAGTGGTGGAGGCCAAGA
**GAPDH**	GGGCTCCGGCTCCAATT	CAGCAGGACACTAGGGAGTCAA
**KLF10-1**	ATGTGAACAAAGCGTGATCA	CCCGCAAGCTCAGGAAGTAG
**KLF10-2**	GGCCGGAGCCAAGAGATT	CAAATGCTTCAGAAATGCAG
**SREBP1**	TCCAAACAAGACCCTCGGG	TGACCCCTGATAGGCACACC
**SLC6A9**	AGTAAACATGGGTCCCAGCA	TCTGTACAAACATGGCTGACCTCT
**MID1IP1**	TTCCCCGACGGCTAATAATAAA	GAATGCACGTGCCTTGGTT
**qPCR primers for microarray validation**		
**Symbol**	**Forward primers**	**Reverse primers**
**Cyclo**	TGCCATCGCCAAGGAGTAG	TGCACAGACGGTCACTCAAA
**PKLR**	AGGAGATGATCAAGGCCGG	AGCATGGTACTCGTGGGAGC
**SREBP1c**	GCCTGGACGCCCCAA	TGATAAGCTGAAGCATGTCTTCGA
**DGAT2**	GCCCTCCTCGAAGATCACCT	GAGCTGACCTGGTTCCCATCTA
**PDK2**	CCGCTGTCCATGAAGCAGTT	GAGGAAGGTGAAGGAGGTTTTCTC
**SCD1**	TCAGAAACACATGCTGATCCTCAT	TGGGTGTTTGCGCACAAG
**GPDI**	CACCGCTTTGCTGCGTACT	GTTCCAGATTTGGTGATGTATTTCC
**TXNIP**	CAGAAGCTCCTCCCTGCTATATG	CAGAGGAGTGGTTGGGCTCTC
**INHBE**	GACTCAACAGACGGAGCAACTG	CGCATGCTCCTGGTGAATG
**SLC6A9**	GCTTCCTACAACAAGTTCCACAATAA	CATAGACGCTGGTGGCACAGT
**TRIB3**	GCAGAAGCCTGTGTGGAGTGT	TGATAAGCACCCAAGCAGGAA
